# What is “healthy” food? A cross-sectional evaluation of foods and beverages consumed by United States adults that satisfy the United States Food and Drug Administration’s updated “healthy” claim criteria

**DOI:** 10.1016/j.ajcnut.2025.11.011

**Published:** 2025-11-25

**Authors:** Anna C Tucker, Euridice Martínez-Steele, Laura E Caulfield, Casey M Rebholz, Julia A Wolfson

**Affiliations:** 1 Department of International Health, Johns Hopkins Bloomberg School of Public Health, Baltimore, MD, United States; 2 Department of Nutrition, School of Public Health and Center for Epidemiological Studies in Health and Nutrition, University of São Paulo, São Paulo, Brazil; 3 Welch Center for Prevention, Epidemiology, and Clinical Research, Johns Hopkins University, Baltimore, MD, United States; 4 Department of Epidemiology, Johns Hopkins Bloomberg School of Public Health, Baltimore, MD, United States; 5 Department of Health Policy and Management, Johns Hopkins Bloomberg School of Public Health, Baltimore, MD, United States

**Keywords:** food quality, food policy, nutrient profiling, food labeling regulation, nutrition labeling, Nova

## Abstract

**Background:**

The Food and Drug Administration (FDA) recently updated criteria for the “healthy” claim displayed on foods and beverages in the United States. However, it is unknown how updated criteria compare with existing methods for evaluating the healthfulness of foods and beverages.

**Objectives:**

To evaluate correlation between “healthy” criteria and 3 nutrient profiling models used to evaluate food and beverage healthfulness, and with Nova food processing classification. Exploratory analyses compare the nutritional profile of “healthy” items and items not meeting "healthy” criteria.

**Methods:**

In this cross-sectional analysis, we identified individual “healthy” items reported in the 2017–2018 National Health and Nutrition Examination Survey. We used descriptive statistics to characterize “healthy” items across food categories, nutrient profiling models (Food Compass 2.0, Nutri-Score, and Health Star Rating), and Nova. We used point-biserial correlation to evaluate correlation between FDA criteria and nutrient profiling models, and rank point-biserial correlation to evaluate correlation with Nova. We used t-tests to compare nutrient content of “healthy” items and items not meeting “healthy” criteria across food categories and Nova categories.

**Results:**

Overall, 14.9% of items qualified for the “healthy” claim. Although the majority of fruits (60.9%), vegetables (59.6%), and nuts and seeds (68.8%) qualified, few meat, poultry, and eggs (3.0%), grains (4.8%), or savory snacks and desserts (1.3%) met criteria. Criteria were moderately correlated with Food Compass 2.0 (r = 0.56), Nutri-Score (r = 0.46), Health Star Rating (r = 0.41), and Nova (0.49). “Healthy” items were lower in saturated fat and sodium and higher in fiber and vitamin C across nearly all food categories and Nova categories.

**Conclusions:**

Findings suggest few foods met “healthy” criteria. Moderate correlations between “healthy” criteria, Nova, and validated nutrient profiling models provide evidence of convergent validity, yet underscore the challenge of classifying foods by healthfulness, and highlight uncertainty about whether discrepancies reflect real differences in model performance and food healthfulness.

## Introduction

Poor dietary quality is a leading contributor to chronic disease morbidity and mortality in the United States [1]. The food environment inundates consumers with convenient, ready-to-heat/ready-to-eat foods high in added sugar, sodium, and saturated fat [2,3]. Meanwhile, limited food access [4], time [5,6], cost [7,8], cooking skills [9], and myriad other factors create barriers to choosing more nutrient-dense foods that form the basis of a healthy dietary pattern. Given this context, accurate, clear, nondeceptive food labeling may help consumers navigate a complex food environment full of persuasive marketing [10,11] and make more informed decisions about the healthfulness of foods and beverages.

Since 1994, the United States Food and Drug Administration (FDA) has regulated use of the “healthy” claim on foods and beverages [12]. Under the original rule, products using the “healthy” claim were required to meet limits on total fat, saturated fat, cholesterol, and sodium, and provide minimum amounts of nutrients to encourage, including vitamin A, vitamin C, and dietary fiber [12]. In 2024, the FDA released new criteria for the “healthy” claim. The new criteria require foods and beverages to provide a minimum amount of ≥1 food group (i.e., fruit, vegetables, dairy, whole grains, seafood, game meat, eggs, legumes, and nuts/seeds), and meet limits for added sugar, sodium, and saturated fat [13]. The “healthy” criteria apply to all foods and beverages sold in the United States, except infant foods and formula.

Updated criteria aim to improve alignment between the “healthy” claim and the Dietary Guidelines for Americans (DGA) and help consumers select foods that can form the foundation of a healthy dietary pattern that promotes health and prevents disease [13]. However, the “healthy” claim is only one of many methods for identifying foods and beverages that support a healthy dietary pattern. Other methods include Food Compass 2.0 [14], Nutri-Score [15], and Health Star Rating [16], which are nutrient profiling models that use various algorithms to assess and rank products according to healthfulness.

Additionally, Nova is a prominent food classification system used to identify ultraprocessed foods (UPFs) that categorizes foods and beverages according to the extent and purpose of processing [17]. Extensive observational [18] and some experimental evidence [19,20] links UPF intake to increased risk of cardiometabolic diseases. However, there is concern that Nova classifies potentially “healthy” foods (e.g., plant-based meat alternatives, flavored yogurts, whole grain breads and snacks, etc.) as UPFs that should be avoided [21]. Therefore, examining alignment between the FDA “healthy” criteria and Nova is important for determining the extent to which UPFs are included/excluded from the updated “healthy” claim.

Methods for evaluating the healthfulness of foods and beverages should undergo rigorous validation prior to implementation [[22], [23], [24]]. Food Compass, Nutri-Score, and Health Star Rating have all demonstrated evidence of criterion validity in United States or European cohorts, with dietary intake predicting lower risk of >1 diet-related diseases [25]. However, FDA “healthy” criteria have not yet been examined for validity. Therefore, the primary aim of this descriptive study is to evaluate the convergent validity of FDA “healthy” criteria by examining correlations between “healthy” criteria and existing nutrient profiling models and Nova. In secondary exploratory analyses, this study will also compare the nutritional profile of FDA-aligned and unaligned foods and beverages, overall and stratified by food category and Nova category.

## Methods

### Data source

Data were obtained from the 2017–2018 Food and Nutrient Database for Dietary Studies (FNDDS) [26] and the 2017–2018 Food Pattern Equivalents Database (FPED) [27]. FNDDS is a publicly available database that contains nutrient values for foods and beverages as they are consumed in the United States [26]. FPED is a corresponding database that converts foods and beverages in FNDDS to equivalent amounts of food pattern components (e.g., cup equivalents of fruits and vegetables) [27]. Foods and beverages in FNDDS and FPED are assigned a unique 8-digit food code. Food codes link to dietary intake data reported in the NHANES. This analysis was limited to the 5153 unique foods and beverages reported in 2017–2018 wave of NHANES. Because the FDA “healthy” criteria are not intended for infant foods and beverages, these items were dropped (n = 204) for an analytic sample of n = 4949 foods and beverages (Supplemental Figure 1 Flowchart). There were no missing data in this analysis. The National Center for Health Statistics Ethics Review Board approves all NHANES protocols.

### Measures

#### FDA criteria

To determine eligibility for the “healthy” claim, Food Group Equivalents (FGE) are calculated for each item based on the Reference Amount Customarily Consumed (RACC). FGE reflects the quantity of key food group(s) provided by the item; key food groups include vegetables, fruits, grains, dairy, and protein foods, including game meat; seafood; eggs; beans, peas, and lentils; and nuts, seeds, and soy [13]. To meet FDA criteria, items must provide a minimum FGE and meet category-specific limits for added sugar, sodium, and saturated fat per RACC [13]. Oils, oil-based spreads, and oil-based dressings may qualify for the “healthy” claim, but may not contribute to the FGE requirement for mixed products, main dish products, or meal products [13]. Table 1 further details the criteria, including nutrient limits specific to each food group and product category.

**TABLE 1:** Food group equivalent requirements and corresponding nutrient limits to qualify for FDA “Healthy” claim.

	Food group equivalent requirements	Added sugar limit	Sodium limit	Saturated fat limit
Individual foods	¾ oz grains	10% DV (5 g)	10% DV (230 mg)	5% DV (1 g)
	2/3 c dairy	5% DV (2.5 g)	10% DV (230 mg)	10% DV (2 g)
	½ c vegetables	2% DV (1 g)	10% DV (230 mg)	5% DV (1 g)
	½ c fruits	2% DV (1 g)	10% DV (230 mg)	5% DV (1 g)
	1 ½ oz game meat^1^	2% DV (1 g)	10% DV (230 mg)	10% DV (2 g)
	1 oz seafood	2% DV (1 g)	10% DV (230 mg)	5% DV (1 g)^2^
	1 oz eggs	2% DV (1 g)	10% DV (230 mg)	10% DV (2 g)
	1 oz beans, peas, lentils	2% DV (1 g)	10% DV (230 mg)	5% DV (1 g)
	1 oz nuts, seeds, and soy	2% DV (1 g)	10% DV (230 mg)	5% DV (1 g)^2^
	100% oils	0%	0%	20% of total fat
	Oil-based spreads	0%	10% DV (230 mg)	20% of total fat
	Oil-based dressing	2% DV (1 g)	10% DV (230 mg)	20% of total fat
Mixed product	At least 1 total FGE, including a minimum of ¼ FGE from ≥2 different food groups	10% DV (5 g)	15% DV (345 mg)	10% DV (2 g) 2
Main dish product	At least 2 total FGE, including a minimum of ½ FGE from ≥2 different food groups	15% DV (7.5 g)	20% DV (460 mg)	15% DV (3 g) 2
Meal product	At least 3 total FGE, including a minimum of ½ FGE from ≥3 different food groups	20% DV (10 g)	30% DV (690 mg)	20% DV (4 g) 2

Abbreviations: oz, ounce; c, cup; g, grams; mg, milligrams; DV, daily value; FGE, food group equivalent.

1 The game meat FGE was interpreted to exclude domestic meat and poultry products. Although domestic meat and poultry products did not contribute to an FGE, a product containing domestic meat or poultry was eligible to qualify for the “healthy” claim if it met other FGE requirements and nutrient limits.

2 Saturated fat limit excludes saturated fat inherent in seafood, nuts, seeds, and soybeans/soy products.

To identify standard serving sizes for items in FNDDS, we used the default serving size provided in the 2017–2018 Portions and Weights database used when quantities are not otherwise specified. We then combined the 2017–2018 FPED with the 2017–2018 Portions and Weights database to obtain food group amounts and nutrient content provided per serving size for all items in 2017–2018 FNDDS. We used the food group amount provided per serving (i.e., FGE) to classify each item into 1 of 4 meal categories: Individual Foods, Mixed Products, Main Dish Products, and Meal Products. Finally, using the nutrient limits specific to each meal category (Table 1), we generated a binary FDA-alignment variable (FDA-aligned/FDA-unaligned), where every item was classified as either meeting FDA “healthy” criteria (FDA-aligned) or not meeting FDA “healthy” criteria (FDA-unaligned). Importantly, we use the terminology FDA-unaligned or not meeting FDA “healthy” criteria because the FDA’s final rule explicitly states that items not meeting “healthy” criteria should not be considered unhealthy. For items with a RACC <50 g or 3 tablespoons, criteria are applied on a per 50 g basis, rather than per RACC. Additionally, any items consisting of a key food group (e.g., fruit, vegetables, low-fat or fat-free dairy) and no added ingredients except for water automatically qualify for the “healthy” claim.

#### Nutrition profiling models

Food Compass 2.0, Nutri-Score, and Health Star Rating are existing systems used to rank foods and beverages according to healthfulness. Food Compass 2.0 is a nutrient profiling model developed by researchers to score foods and beverages from 1–100 [14]. Scores are based on 54 factors, across 9 domains (nutrient ratios, vitamins, minerals, food-based ingredients, additives, processing, lipids, fiber and protein, and phytochemicals), all estimated per 100 kcal. Scores can be further categorized to identify “foods to encourage” (70–100), “foods to moderate” (31–69), and “foods to minimize” (1–30) [14]. Nutri-Score and Health Star Rating are front-of-pack labels, both based on the British Food Standards Agency nutrient profiling system. The underlying nutrient profiling system classifies foods based on favorable characteristics (protein, fiber, and fruits, vegetables, or legumes) and unfavorable characteristics (energy, sugar, saturated fat, and sodium), which are estimated per 100 g or 100 mL for foods and beverages, respectively. Nutri-Score categorizes foods into 1 of 5 groups (A, B, C, D, or E), where “A” has the highest nutritional quality and “E” has the lowest nutritional quality, and has been adopted as a front-of-pack label in several European countries [28]. Health Star Rating categorizes products into 1 of 10 groups, from 0.5 stars (least healthy) to 5 stars (most nutritious), and is used as a front-of-pack label used in Australia and New Zealand [16] We used scores provided in the Supplementary Material of Barrett et al. [14], to assign Food Compass, Nutri-Score, and Health Star Rating scores to each item in 2017–2018 FNDDS.

#### Nova classification

To describe items in FNDDS based on degree of processing, we used the Nova classification system. Nova classifies items into 1 of 4 groups according to the “extent and purpose of the industrial processing that they undergo”[17]. Nova accounts for the physical, biological, and chemical methods used to produce foods and beverages, including the use of food additives [29]. Nova categories include: Group 1 unprocessed/minimally processed foods; Group 2 processed culinary ingredients such as sugar, salt, and fats; Group 3 processed foods, which are produced by the food industry and combine Group 1 and Group 2 ingredients; and Group 4 UPFs, which are industrial formulations, “made mostly or entirely from substances derived from foods and cosmetic additives, with little if any intact Group 1 food”[17].

Full definitions and examples from each Nova category are presented in Supplemental Table 1. Briefly, minimally processed foods are food and beverages that have undergone no processing or minimal processing to improve storage time, food safety, digestibility, or palatability [17]. Examples include grinding, pasteurization, freezing, drying, roasting, and fermentation. Processed culinary ingredients include sugar, plant oils, animal fats, and salt that have been extracted from minimally processed foods or nature and are used in culinary preparations [17]. Processed foods are manufactured by the food industry and are combinations of minimally processed foods and culinary ingredients (e.g., bread, canned fruits and vegetables, cured meat, etc) [17]. Lastly, UPFs are industrial formulations of several ingredients with little to no whole foods. Formulations include processed culinary ingredients, but also contain additives such as artificial colors, emulsifiers, sweeteners, and other additives and substances not typically used in culinary preparations [17].

Methods for classifying foods and beverages in FNDDS according to Nova have been previously described [29]. Briefly, we used the “main food description” and “additional food description” to determine whether items were likely homemade/artisanal or purchased as ready-to-eat/heat. We classified ready-to-eat/heat at the food code level, whereas homemade/artisanal items were disaggregated into corresponding Standard Reference codes, which were then classified according to Nova. For the purpose of this analysis, items consisting of >1 Nova group were categorized into a single Nova group according to the Nova group contributing >50% of weight in grams.

#### Nutrients of interest

In secondary analyses, we aimed to compare the nutrient content of FDA-aligned and FDA-unaligned items overall, and by food category and Nova category. We did not examine nutrient content of FDA-aligned and unaligned items across nutrient profiling models because, unlike Nova, these approaches already account for nutritional profile. Though appendices include all macro and micronutrients for all food categories and Nova categories, analyses presented in the main tables focus on food categories with the greatest number of FDA-aligned items, as well as a prespecified list of macro and micronutrients of public health interest. Energy and core macronutrients (protein, fat, carbohydrate) were included to describe the basic macronutrient composition of items. Sodium, added sugar, and saturated fat were included because they are used to determine “healthy” claim eligibility [13]. We also examined nutrients that were used to determine “healthy” claim eligibility under the original 1994 rule, including fiber, vitamin A, vitamin C, calcium, and iron [12]. Monounsaturated and polyunsaturated fats were included because they are recommended in place of saturated fat to promote cardiometabolic health [30], and, unlike the original “healthy” criteria, updated criteria intend to capture foods that are a good source of healthy fats [13]. Remaining nutrients were included as nutrients of public health interest, given the potential for inadequate intake among the United States population: folate, vitamin D, vitamin E, vitamin K, choline, magnesium, and potassium [[31], [32], [33]].

### Statistical analysis

We described the distribution of FDA-aligned and FDA-unaligned items overall, by food category, nutrient profiling model scores, and Nova category. For each nutrient profiling model, we compared the score distributions between FDA-aligned and FDA-unaligned items, overall and by food category, and tested significance using Mann-Whitney U tests. To test convergent validity between FDA-alignment and Food Compass 2.0, Nutri-Score, and Health Star Rating, overall and by food category, we used point-biserial correlation because FDA-alignment is dichotomous, and nutrient profiling models are scored on an interval scale [34]. To test convergent validity between FDA-alignment and Nova, overall and by food category, we used rank point-biserial correlation because Nova categories do not follow an interval scale [35]. For all correlation coefficients, we obtained 95% confidence intervals and *P* values using a nonparametric bootstrap procedure that included 1000 resamples [36]. For secondary analyses, we used t-tests to compare mean log-transformed nutrient content of FDA-aligned versus FDA-unaligned items overall and across food categories and Nova categories. We report untransformed median and IQR nutrient values. Using Bonferroni corrected alpha, significance was considered at *P* < 0.0001. All analyses were performed using Stata version 18.0.

## Results

Foods and beverages are described overall and by FDA-alignment across food categories, nutrient profiling model scores, and Nova categories in Table 2 [14]. Of the 4949 items in FNDDS, 14.9% were FDA-aligned. Although 68.8% of nuts and seeds, 60.9% of fruits, and 59.6% of vegetables were FDA-aligned, 3.0% of meat, poultry, and eggs, 1.3% of snacks and desserts, and 4.8% of grains were FDA-aligned. By Food Compass 2.0, 50% of items categorized as “Foods to Encourage” were FDA-aligned. Likewise, 55.5% of foods in the highest Nutri-Score category, “A,” were FDA-aligned, whereas 74.4% and 57.5% of items receiving a 4.5 or 5, respectively, through Health Star Rating were FDA-aligned. Among minimally processed foods, 27.9% were classified as FDA-aligned, whereas 17.3%, 14.8%, and 2.1% of processed culinary ingredients, processed foods, and UPFs were considered FDA-aligned, respectively. Supplemental Table 2 lists the UPFs from FNDDS that qualified for the “healthy” claim, which were largely beverages or whole grain snack products.

**TABLE 2:** Distribution of FDA-aligned and FDA-unaligned foods and beverages consumed in the United States NHANES/FNDDS 2017 – 2018, overall and by food category, Nutrient Profiling Model, and Nova classification.

	Total items	FDA-aligned N (%)	FDA-unaligned N (%)
Overall	4949	735 (14.9)	4214 (85.2)
Food category			
Beverages	422 (8.5)	98 (23.2)	324 (76.8)
Grains	437 (8.8)	21 (4.8)	416 (95.2)
Vegetables	525 (10.6)	313 (59.6)	212 (40.4)
Fruits	110 (2.2)	67 (60.9)	43 (39.1)
Legumes	80 (1.6)	35 (43.8)	45 (56.3)
Nuts and Seeds	77 (1.6)	53 (68.8)	24 (31.2)
Meat, poultry, and eggs	472 (9.5)	14 (3.0)	458 (97.0)
Seafood	193 (3.9)	17 (8.8)	176 (91.2)
Dairy	196 (4.0)	22 (11.2)	174 (88.8)
Fats and oils	68 (1.4)	8 (11.8)	60 (88.2)
Mixed dishes	1510 (30.5)	69 (4.6)	1,441(95.4)
Sauces and condiments	179 (3.6)	9 (5.1)	169 (94.9)
Savory snacks and desserts	680 (13.7)	9 (1.3)	671 (98.7)
Food Compass 2.0^1^^,^^2^			
Foods to encourage	1166 (24.5)	594 (50.9)	572 (49.1)
Foods to moderate	2178 (45.8)	141 (6.5)	2037 (93.5)
Foods to minimize	1605 (33.7)	0	1605 (100.0)
Nutri-Score^1^^,^^3^			
A	808 (17.0)	448 (55.5)	(44.6)
B	730 (15.4)	118 (16.2)	612 (83.8)
C	1265 (26.6)	74 (5.9)	1191 (94.2)
D	1196 (25.2)	31 (2.6)	1165 (97.4)
E	757 (15.9)	18 (2.4)	739 (97.6)
Health Star Rating^1^^,^^4^			
5.0	273 (5.7)	203 (74.4)	70 (25.6)
4.5	320 (6.7)	184 (57.5)	136 (42.5)
4.0	912 (19.2)	185 (20.3)	727 (79.7)
3.5	1205 (25.3)	69 (5.7)	1136 (94.3)
3.0	579 (12.2)	20 (3.5)	559 (96.6)
2.5	289 (6.1)	13 (4.5)	276 (95.5)
2.0	423 (8.9)	5 (1.2)	418 (98.8)
1.5	337 (7.1)	5 (1.5)	332 (98.5)
1.0	199 (4.2)	5 (2.5)	194 (97.5)
0.5	219 (4.6)	0	219 (100.0)
Nova^5^			
Minimally processed foods	2257 (45.6)	629 (27.9)	1628 (72.1)
Processed culinary ingredients	52 (1.1)	9 (17.3)	43 (82.7)
Processed foods	325 (6.6)	48 (14.8)	277 (85.2)
Ultraprocessed foods	2167 (43.8)	46 (2.1)	2121 (97.9)
Other^6^	148 (3.0)	3 (2.0)	145 (98.0)

Abbreviations: FDA, Food and Drug Administration; FNDDS, Food and Nutrient Database for Dietary Studies.

1 Analyses restricted to 4756 items that were assigned a Food Compass Score in Barrett et al. [14]. This excluded infant formula, baby foods, specialized dietary foods, alcohol, and products providing <5 kcal per 100 g.

2 Foods and beverages were scored according to Food Compass 2.0 on a continuous scale from 0–100. Categories reflect the following: 0–30 are “foods to minimize,” 31–69 are “foods to moderate,” and > 70 are “foods to encourage.”

3 Foods and beverages were scored according to Nutri-Score on a scale from A–E, where E corresponds to least healthful and A corresponds to most healthful.

4 Foods and beverages were scored according to Health Star Rating using categories from 0.5–5, where items receiving a 0.5 were the lowest scored or least nutritious items, and items receiving 5 were the highest scored or most nutritious items.

5 Foods and beverages in FNDDS consisting of >1 Nova group were categorized into a single Nova group according to the Nova group contributing >50% of weight in grams.

6 “Other” includes foods and beverages with no single Nova group contributing >50% of weight in grams.

Correlations between FDA-alignment criteria and Food Compass 2.0, Nutri-Score, Health Star Rating, and Nova are presented in Table 3 [14], overall and by food category. Agreement was highest for FDA-alignment and Food Compass 2.0 (0.56; 95% CI: 0.54, 0.58; *P* < 0.001), followed by Nova (0.49; 95% CI: 0.47, 0.52, *P* < 0.001), Nutri-Score (0.46; 95% CI: 0.43, 0.48; *P* < 0.001), and Health Star Rating (0.41; 95% CI: 0.39, 0.43; *P* < 0.001). There was substantial variation in correlation across food categories. For Food Compass 2.0, correlations were highest for fats and oils (0.85; 95% CI: 0.75, 0.96; *P* < 0.001), fruits (0.80; 95% CI: 0.74, 0.86; *P* <0.001), and beverages (0.68; 95% CI: 0.61, 0.74; *P* < 0.001), and lowest for meat, poultry, and eggs (0.14; 95% CI: 0.09, 0.20; *P* < 0.001); seafood (0.19; 95% CI: 0.06, 0.32; *P* < 0.001), and savory snacks and desserts (0.27; 95% CI: 0.18, 0.36; *P* < 0.001). For Nutri-Score, the strongest correlation was observed for fats and oils (0.81; 95% CI: 0.69, 0.94; *P* < 0.001), with no significant correlation for beverages. Correlations for Health Star Rating ranged from 0.13 (95% CI: 0.07, 0.19; *P* < 0.001) for meat, poultry, and eggs to 0.67 (95% CI: 0.54, 0.80; *P* < 0.001) for nuts and seeds. For Nova, correlations were strongest for sauces and condiments (0.88; 95% CI: 0.82, 0.93; *P* < 0.001), with no significant correlations for grains, seafood, or savory snacks and desserts.

**TABLE 3:** Correlation between FDA-alignment and Food Compass 2.0, Nutri-Score, Health Star Rating, and Nova Overall and by food category for foods and beverages consumed in the United States NHANES/FNDDS 2017–2018 (n = 4756)^1^.

	Number of items (%)	FDA-alignment and Food Compass 2.0^2^		FDA-alignment and Nutri-Score 2		FDA-alignment and Health Star Rating 2		FDA-alignment and Nova 3	
		r (95% CI) 4	*P* value	r (95% CI)	*P* value	r (95% CI)	*P* value	r (95% CI)	*P* value
Overall	4756	0.56 (0.54, 0.58)	<0.001	0.46 (0.43, 0.48)	<0.001	0.41 (0.39, 0.43)	<0.001	0.49 (0.47, 0.52)	<0.001
Food category									
Beverages	254 (5.3)	0.68 (0.61, 0.74)	<0.001	−0.02 (−0.13, 0.09)	0.72	0.22 (0.11, 0.32)	<0.001	0.62 (0.53, 0.72)	<0.001
Grains	431 (9.1)	0.30 (0.23, 0.38)	<0.001	0.37 (0.28, 0.45)	<0.001	0.26 (0.19, 0.33)	<0.001	0.16 (−0.07, 0.38)	0.18
Vegetables	519 (10.9)	0.65 (0.60, 0.71)	<0.001	0.68 (0.62, 0.73)	<0.001	0.53 (0.48, 0.58)	<0.001	0.20 (0.14, 0.27)	<0.001
Fruits	110 (2.3)	0.80 (0.74, 0.86)	<0.001	0.27 (0.08, 0.45)	<0.01	0.62 (0.50, 0.74)	<0.001	0.71 (0.57, 0.85)	<0.001
Legumes	80 (1.7)	0.42 (0.28, 0.57)	<0.001	0.27 (0.13, 0.41)	<0.001	0.28 (0.13, 0.44)	<0.001	0.58 (0.41, 0.76)	<0.001
Nuts and seeds	77 (1.6)	0.60 (0.44, 0.75)	<0.001	0.72 (0.63, 0.82)	<0.001	0.67 (0.54, 0.80)	<0.001	0.38 (0.14, 0.63)	<0.01
Meat, poultry, and eggs	472 (9.9)	0.14 (0.09, 0.20)	<0.001	0.15 (0.08, 0.23)	0.02	0.13 (0.07, 0.19)	<0.001	0.26 (0.11, 0.41)	0.001
Seafood	193 (4.1)	0.19 (0.06, 0.32)	<0.01	0.23 (0.16, 0.30)	<0.001	0.18 (0.10, 0.27)	<0.001	0.08 (−0.05, 0.21)	0.22
Dairy	195 (4.1)	0.57 (0.47, 0.66)	<0.001	0.46 (0.32, 0.59)	<0.001	0.30 (0.20, 0.41)	<0.001	0.70 (0.61, 0.79)	<0.001
Fats and oils	68 (1.4)	0.85 (0.75, 0.96)	<0.001	0.81 (0.69, 0.94)	<0.001	0.59 (0.41, 0.76)	<0.001	0.83 (0.73, 0.94)	<0.001
Mixed dishes	1508 (31.7)	0.36 (0.32, 0.41)	<0.001	0.26 (0.22, 0.31)	<0.001	0.21 (0.17, 0.24)	<0.001	0.39 (0.31, 0.46)	<0.001
Sauces and condiments	174 (3.6)	0.33 (0.22, 0.45)	<0.001	0.50 (0.37, 0.64)	<0.001	0.30 (0.19, 0.41)	<0.001	0.88 (0.82, 0.93)	<0.001
Savory snacks and desserts	675 (14.2)	0.27 (0.18, 0.36)	<0.001	0.24 (0.14, 0.33)	<0.001	0.26 (0.17, 0.34)	<0.001	0.00 (−0.22, 0.22)	0.99

Abbreviations: FDA, Food and Drug Administration; FNDDS, Food and Nutrient Database for Dietary Studies.

1 Analyses restricted to 4756 items that were assigned a Food Compass Score in Barrett et al. [14]. This excluded infant formula, baby foods, specialized dietary foods, alcohol, and products providing <5 kcal per 100 g.

2 Point-biserial correlations (r) were used to assess correlations between FDA-alignment (0 = FDA-unaligned; 1 = FDA-aligned) and Food Compass 2.0 scores (1–100), Nutri-Score (1 = “E,” 2 = “D,” 3 = “C,” 4 = “B,” 5 = “A”), and Health Star Rating (0.5–5.0).

3 Rank point-biserial correlation (r) was used to assess correlation between FDA-alignment (0 = FDA-unaligned; 1 = FDA-aligned) and Nova food processing categories, which were reverse coded (1 = Group 4 ultraprocessed foods, 2 = Group 3 processed foods, 3 = Group 2 processed culinary ingredients, and 4 = Group 1 unprocessed/minimally processed foods) to facilitate clearer interpretation.

4 95% confidence intervals and *P* values were obtained using a nonparametric bootstrap procedure that included 1,000 resamples.

**FIGURE 1:**
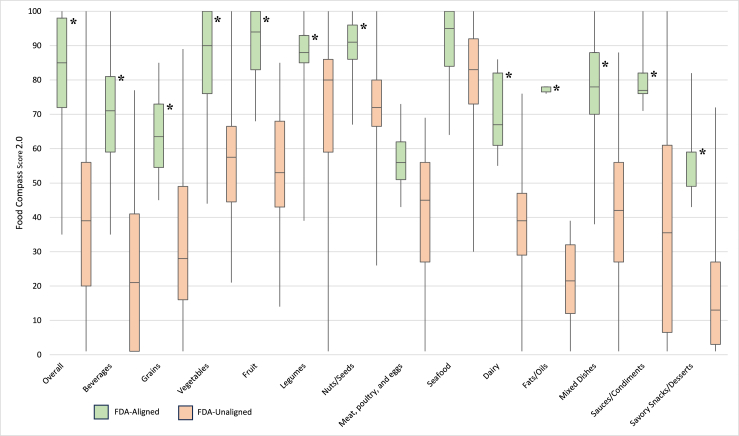
Food Compass 2.0 score distributions by FDA-alignment, overall, and stratified by food category consumed in the United States NHANES/FNDDS 2017–2018 (n = 4756).^1,2,3^ Abbreviations: FDA, Food and Drug Administration; FNDDS, Food and Nutrient Database for Dietary Studies. ^1^Analyses restricted to 4756 items that were assigned a Food Compass Score in Barrett et al. [14]. This excluded infant formula, baby foods, specialized dietary foods, alcohol, and products providing <5 kcal per 100 g. ^2^Food Compass 2.0 scores foods and beverages on a continuous scale from 0–100, where items scoring 0–30 are “foods to minimize,” items scoring 31–69 are “foods to moderate,” and items scoring > 70 are “foods to encourage.” ^3^Median, IQR, and minimum/maximum values for Food Compass 2.0 scores, presented overall and stratified by food category and FDA-alignment. ∗*P* < 0.0001 from Mann-Whitney U tests comparing Food Compass 2.0 score distributions between FDA-aligned and FDA-unaligned items.

**FIGURE 2:**
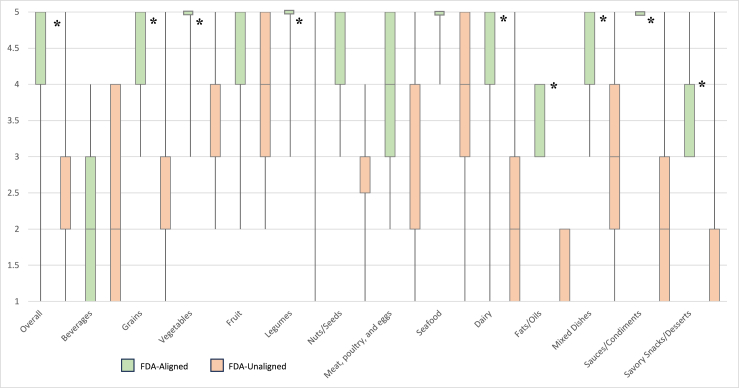
Nutri-Score distributions by FDA-alignment, overall, and stratified by food category consumed in the United States NHANES/FNDDS 2017–2018 (n = 4756).^1,2,3^ Abbreviations: FDA, Food and Drug Administration; FNDDS, Food and Nutrient Database for Dietary Studies. ^1^Analyses restricted to 4756 items that were assigned a Nutri-Score value in Barrett et al. [14]. This excluded infant formula, baby foods, specialized dietary foods, alcohol, and products providing <5 kcal per 100 g. ^2^Nutri-Score categories were converted to numerical scores: 1 = “E,” 2 = “D,” 3 = “C,” 4 = “B,” 5 = “A,” where E corresponds to least healthful and A corresponds to most healthful. ^3^Median, IQR, and minimum/maximum values for Nutri-Score, presented overall and stratified by food category and FDA-alignment. ∗*P* < 0.0001 from Mann-Whitney U tests comparing Nutri-Score distributions between FDA-aligned and FDA-unaligned items.

**FIGURE 3:**
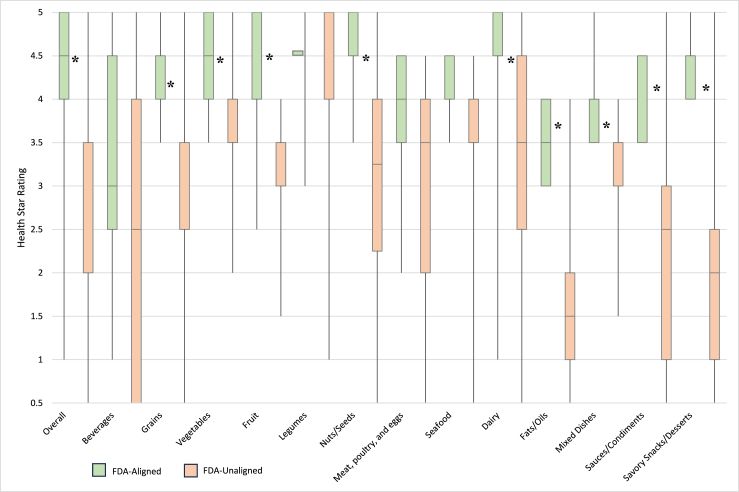
Health Star Rating distributions by FDA-alignment, overall, and stratified by food category consumed in the United States NHANES/FNDDS 2017–2018 (n = 4756).^1,2,3^ Abbreviations: FDA, Food and Drug Administration; FNDDS, Food and Nutrient Database for Dietary Studies. ^1^Analyses restricted to 4756 items that were assigned a Health Star Rating value in Barrett et al. [14]. This excluded infant formula, baby foods, specialized dietary foods, alcohol, and products providing <5 kcal per 100 g. ^2^Foods and beverages were scored according to Health Star Rating using categories from 0.5–5, where items receiving a 0.5 were the lowest scored or least nutritious items, and items receiving 5 were the highest scored or most nutritious items. ^3^Median, IQR, and minimum/maximum values for Health Star Rating, presented overall and stratified by food category and FDA-alignment. ∗*P* < 0.0001 from Mann-Whitney U tests comparing Health Star Rating distributions between FDA-aligned and FDA-unaligned items.

Distributions of Food Compass 2.0 scores for FDA-aligned and FDA-unaligned items are presented overall and across food categories in Figure 1 [14]. Overall, FDA-aligned items had higher median Food Compass 2.0 scores (85, IQR: 72, 98) than FDA-unaligned items (39, IQR: 20, 56), *P* < 0.0001. Food Compass 2.0 scores were significantly higher (*P* < 0.0001) among FDA-aligned items across all food categories except meat, poultry, and eggs, and seafood. Most FDA-aligned items across food categories had median Food Compass 2.0 scores >70, consistent with “foods to be encouraged.” Grains (63.5, IQR: 54.5, 73); dairy (67, IQR: 61, 82) savory snacks and desserts (59, IQR: 49, 59); and meat, poultry, and eggs (56, IQR: 51, 62) were the only food categories where FDA-aligned items had median Food Compass 2.0 scores <70, corresponding to “foods to be consumed in moderation.” Across FDA-unaligned food categories, there was more variation in Food Compass 2.0 scores, with median scores ranging from 83 (IQR: 73, 92) among FDA-unaligned seafood to 13 (IQR: 3, 27) among FDA-unaligned savory snacks and desserts. Distributions of Nutri-Score by FDA-alignment overall and across food categories are presented in Figure 2 [14], whereas distributions of Health Star Rating scores by FDA-alignment overall and across food categories are presented in Figure 3 [14]. Overall, FDA-aligned items had a higher median Nutri-Score (5: IQR: 4,5) compared with Nutri-Score for overall FDA-unaligned items (3: IQR: 2,3), *P* < 0.0001. FDA-aligned items also had a higher median Health Star Rating (4.5, IQR: 4,5), compared with overall Health Star Rating (3.5; IQR: 2, 3.5), *P* < 0.0001 for FDA-unaligned items.

Median values of macronutrients, vitamins, and minerals per 100 g of FDA-aligned and FDA-unaligned items are shown in TABLE 4, TABLE 5, TABLE 6. Results are presented overall, by Nova category, and by the 4 food categories with the greatest number of FDA-aligned items: fruits, vegetables, beverages, and mixed dishes. Compared to FDA-unaligned minimally processed foods, FDA-aligned minimally processed foods were lower in energy (58 kcal vs 154 kcal, *P* < 0.0001), saturated fat, choline, iron, and sodium (123 mg vs 337 mg, *P* < 0.0001) and higher in fiber (2.0 g vs 0.7 g, *P* < 0.0001), vitamin C, vitamin K, and potassium. Compared with FDA-unaligned UPFs, FDA-aligned UPFs were higher in fiber and lower in saturated fat (0.5 g vs 2.3 g, *P* < 0.0001) and sodium (163 mg vs 394 mg, *P* < 0.0001).

**TABLE 4:** Median macronutrients per 100 g of FDA-aligned and FDA-unaligned foods and beverages consumed in the United States NHANES/FNDDS 2017–2018, overall and stratified by Nova and food category.

	Overall median (IQR)	FDA-unaligned median (IQR)	FDA-aligned median (IQR)	*P* value^1^
Overall (N)	4949	4214	735	
Energy, kcal	182.0 (96.0–297.0)	202.0 (120.0–308.0)	63.0 (41.0–135.0)	<0.0001
Protein, g	6.4 (2.4–11.9)	7.1 (3.2–12.5)	2.4 (1.1–6.3)	<0.0001
Carbohydrate, g	15.1 (5.6–30.3)	16.5 (6.0–34.4)	9.5 (4.6–18.5)	<0.0001
Sugars, added, g	0.0 (0.0–3.9)	0.4 (0.0–5.8)	0.0	<0.0001
Fiber, total dietary, g	1.1 (0.2–2.4)	1.0 (0.1–2.2)	2.0 (1.0–3.8)	<0.0001
Saturated fat, g	1.6 (0.5–4.0)	2.0 (0.7–4.3)	0.5 (0.0–0.9)	<0.0001
Monounsaturated fat, g	2.1 (0.5–4.7)	2.5 (0.8–5.0)	0.6 (0.0–1.2)	<0.0001
Polyunsaturated fat, g	1.1 (0.3–2.8)	1.3 (0.4–3.0)	0.5 (0.1–1.3)	<0.0001
Minimally processed foods (N)	2257	1628	629	
Energy, kcal	133.0 (76.0–190.0)	154.0 (115.0–203.5)	58.0 (40.0–113.0)	<0.0001
Protein, g	5.9 (2.3–13.9)	8.1 (3.5–18.7)	2.2 (1.1–4.3)	<0.0001
Carbohydrate, g	10.0 (3.6–18.1)	10.9 (1.6–18.4)	9.0 (4.6–16.0)	0.13
Sugars, added, g	0.0 (0.0–0.0)	0.0 (0.0–0.5)	0.0 (0.0–0.0)	<0.001
Fiber, total dietary, g	1.0 (0.2–1.9)	0.7 (0.0–1.4)	2.0 (1.1–3.4)	<0.0001
Saturated fat, g	1.2 (0.5–2.7)	1.7 (0.9–3.3)	0.4 (0.0–0.8)	<0.0001
Monounsaturated fat, g	1.6 (0.5–3.4)	2.2 (1.0–4.1)	0.3 (0.0–1.1)	<0.0001
Polyunsaturated fat, g	1.0 (0.3–2.2)	1.3 (0.5–2.6)	0.4 (0.1–1.1)	<0.0001
Processed culinary ingredients (N)	52	43	9	
Energy, kcal	323.5 (188.0–717.0)	292.0 (176.0–474.0)	884.0 (884.0–884.0)	<0.01
Protein, g	0.7 (0.0–3.1)	0.9 (0.1–3.6)	0.0	—
Carbohydrate, g	10.6 (0.2–66.1)	23.5 (3.4–75.0)	0.0	—
Sugars, added, g	0.0 (0.0–40.3)	0.0 (0.0–68.0)	0.0	—
Fiber, total dietary, g	0.0 (0.0–0.2)	0.0 (0.0–0.2)	0.0	—
Saturated fat, g	7.0 (0.0–16.6)	4.6 (0.0–21.2)	10.3 (8.2–13.7)	0.99
Monounsaturated fat, g	3.3 (0.0–19.7)	2.3 (0.0–7.8)	39.7 (22.8–63.3)	0.03
Polyunsaturated fat, g	0.5 (0.0–3.2)	0.2 (0.0–1.7)	41.2 (17.4–54.7)	<0.001
Processed foods (N)	325	277	48	
Energy, kcal	173 (84.0–329.0)	170.0 (84.0–299.0)	182.0 (68.0–605.5)	0.05
Protein, g	8.7 (1.5–21.0)	8.7 (1.3–21.6)	8.7 (2.2–20.3)	0.61
Carbohydrate, g	7.9 (2.6–18.8)	5.9 (2.1–16.3)	14.3 (8.6–21.1)	<0.01
Sugars, added, g	0.0 (0.0–1.1)	0.0 (0.0–1.6)	0.0	0.03
Fiber, total dietary, g	0.8 (0.0–2.6)	0.4 (0.0–2.2)	4.9 (1.8–9.3)	<0.001
Saturated fat, g	1.6 (0.1–6.3)	1.6 (0.1–5.8)	2.2 (0.6–6.9)	0.52
Monounsaturated fat, g	2.1 (0.1–7.3)	2.1 (0.1–6.0)	2.6 (0.6–28.5)	0.02
Polyunsaturated fat, g	0.9 (0.2–2.7)	0.8 (0.1–2.3)	1.5 (0.5–13.6)	<0.001
Ultraprocessed foods (N)	2169	2123	46	
Energy, kcal	274.0 (136.0–379.0)	275.0 (138.0–379.0)	236.0 (53.0–353.0)	0.01
Protein, g	6.5 (3.0–10.8)	6.5 (3.0–10.8)	7.5 (0.6–12.4)	0.32
Carbohydrate, g	27.5 (11.8–55.9)	27.5 (11.9–55.9)	29.0 (6.9–65.8)	0.47
Sugars, added, g	2.6 (0.0–14.0)	2.7 (0.0–14.4)	0.0 (0.0–1.0)	0.01
Fiber, total dietary, g	1.5 (0.3–2.9)	1.4 (0.3–2.9)	3.2 (0.3–7.7)	<0.0001
Saturated fat, g	2.2 (0.6–5.0)	2.3 (0.6–5.1)	0.5 (0.1–0.9)	<0.0001
Monounsaturated fat, g	2.6 (0.6–5.6)	2.7 (0.6–5.7)	0.5 (0.0–1.6)	<0.0001
Polyunsaturated fat, g	1.4 (0.4–3.5)	1.4 (0.4–3.5)	0.9 (0.1–1.9)	0.23
Beverages (N)	422	324	98	
Energy, kcal	46.0 (21.0–84.0)	50.5 (26.5–90.0)	38.5 (1.0–53.0)	<0.0001
Protein, g	0.2 (0.0–1.2)	0.2 (0.0–1.5)	0.4 (0.1–0.7)	0.63
Carbohydrate, g	7.7 (1.9–12.7)	7.7 (2.6–13.1)	7.7 (0.2–12.2)	0.32
Sugars, added, g	0.4 (0.0–7.7)	4.4 (0.0–8.7)	0.0	<0.0001
Fiber, total dietary, g	0.0 (0.0–0.2)	0.0 (0.0–0.1)	0.2 (0.0–0.8)	0.10
Saturated fat, g	0.0 (0.0–0.1)	0.0 (0.0–0.2)	0.0	<0.0001
Monounsaturated fat, g	0.0 (0.0–0.1)	0.0 (0.0–0.2)	0.0	<0.0001
Polyunsaturated fat, g	0.0 (0.0–0.1)	0.0 (0.0–0.1)	0.0 (0.0–0.1)	<0.01
Vegetables (N)	525	212	313	
Energy, kcal	68.0 (43.0–123.0)	125.0 (93.0–191.5)	50.0 (35.0–74.0)	<0.0001
Protein, g	2.1 (1.4–3.1)	2.3 (1.9–3.8)	1.9 (1.3–3.0)	<0.0001
Carbohydrate, g	9.7 (5.0–18.4)	17.8 (12.0–20.9)	6.7 (4.4–11.6)	<0.0001
Sugars, added, g	0.0	0.0	0.0	0.11
Fiber, total dietary, g	2.1 (1.5–2.9)	1.8 (1.4–2.4)	2.5 (1.7–3.2)	<0.0001
Saturated fat, g	0.8 (0.1–1.2)	1.4 (0.9–2.5)	0.5 (0.1–0.8)	<0.0001
Monounsaturated fat, g	1.0 (0.1–1.5)	1.6 (1.0–3.9)	0.9 (0.0–1.0)	<0.0001
Polyunsaturated fat, g	0.9 (0.2–1.3)	1.3 (0.5–4.0)	0.8 (0.1–1.0)	<0.0001
Fruits (N)	110	43	67	
Energy, kcal	60.5 (48.0–150.0)	70.0 (53.0–159.0)	57.0 (43.0–126.0)	0.09
Protein, g	0.8 (0.5–1.4)	0.6 (0.4–1.3)	0.9 (0.7–1.5)	<0.01
Carbohydrate, g	15.0 (11.9–22.8)	16.2 (13.5–26.1)	13.9 (11.1–19.2)	0.43
Sugars, added, g	0.0 (0.0–3.3)	5.3 (1.6–8.0)	0.0	0.13
Fiber, total dietary, g	2.1 (1.4–3.6)	1.5 (1.0–2.4)	2.4 (1.6–4.5)	<0.001
Saturated fat, g	0.0 (0.0–0.1)	0.0 (0.0–0.6)	0.0 (0.0–0.1)	0.20
Monounsaturated fat, g	0.0 (0.0–0.1)	0.0 (0.0–0.4)	0.0 (0.0–0.1)	0.26
Polyunsaturated fat, g	0.1 (0.0–0.2)	0.1 (0.0–0.2)	0.1 (0.1–0.2)	0.79
Mixed dishes (N)	1510	1441	69	
Energy, kcal	165.0 (117.0–245.0)	170.0 (121.0–247.0)	114.0 (97.0–123.0)	<0.0001
Protein, g	8.8 (5.4–12.2)	9.0 (5.5–12.2)	6.0 (2.7–9.2)	<0.0001
Carbohydrate, g	16.4 (7.7–22.7)	16.4 (7.7–22.8)	18.5 (5.3–21.9)	<0.19
Sugars, added, g	0.0 (0.0–1.6)	0.0 (0.0–1.7)	0.0	0.30
Fiber, total dietary, g	1.1 (0.7–1.8)	1.1 (0.7–1.8)	1.8 (1.3–2.7)	<0.0001
Saturated fat, g	2.3 (1.0–4.2)	2.5 (1.0–4.3)	0.7 (0.4–1.0)	<0.0001
Monounsaturated fat, g	2.6 (1.3–4.4)	2.7 (1.4–4.4)	1.1 (0.9–2.4)	<0.0001
Polyunsaturated fat, g	1.4 (0.7–2.7)	1.5 (0.8–2.7)	1.1 (0.5–1.9)	0.10

Abbreviations: FDA, Food and Drug Administration; FNDDS, Food and Nutrient Database for Dietary Studies; kcal, kilocalorie; g, grams.

1 *P* values from t-tests comparing mean log-transformed nutrient values between FDA-aligned and FDA-unaligned groups.

**TABLE 5:** Median vitamins per 100 g of FDA-aligned and FDA-unaligned foods and beverages consumed in the United States NHANES/FNDDS 2017–2018, overall and stratified by Nova and Food category.

	Overall median (IQR)	FDA-unaligned median (IQR)	FDA-aligned median (IQR)	*P* value^1^
Overall (N)	4949	4214	735	
Vitamin A, mcg_RAE,	18.0 (1.0–62.0)	18.0 (1.0–62.0)	14.0 (1.0–64.0)	0.63
Folate, mcg_DFE	23.0 (7.0–61.0)	23.0 (7.0–63.0)	22.0 (10.0–53.0)	0.87
Vitamin C, mg	0.5 (0.0–4.3)	0.4 (0.0–2.9)	5.1 (0.8–18.8)	<0.0001
Vitamin D, mcg	0 (0.0–0.3)	0.1 (0.0–0.4)	0.0	0.09
Vitamin E, mg	0.5 (0.2–1.1)	0.5 (0.2–1.2)	0.5 (0.2–1.1)	0.24
Vitamin K, mcg	3.6 (0.8–9.7)	3.4 (0.7–8.6)	6.5 (1.1–25.6)	<0.0001
Choline, mg	19.5 (9.9–41.3)	20.8 (10.8–44.3)	14.4 (7.1–27.1)	<0.0001
Minimally processed foods (N)	2257	1628	629	
Vitamin A, mcg_RAE,	20.0 (4.0–65.0)	20.0 (5.0–64.0)	19.0 (1.0–80.0)	<0.01
Folate, mcg_DFE	21.0 (8.0–48.0)	20.5 (8.0–46.0)	21.0 (10.0–51.0)	<0.001
Vitamin C, mg	1.3 (0.0–7.4)	0.5 (0.0–4.1)	7.0 (1.1–22.4)	<0.0001
Vitamin D, mcg	0.0 (0.0–0.3)	0.1 (0.0–0.5)	0.0	0.13
Vitamin E, mg	0.5 (0.2–1.0)	0.5 (0.3–1.0)	0.5 (0.2–1.0)	0.34
Vitamin K, mcg	4.6 (0.8–12.3)	4.1 (0.8–10.0)	7.3 (1.7–28.8)	<0.0001
Choline, mg	22.6 (10.7–61.2)	32.0 (13.8–72.8)	13.4 (7.1–24.6)	<0.0001
Processed culinary ingredients (N)	52	43	9	
Vitamin A, mcg_RAE,	0.0 (0.0–119.0)	8.0 (0.0–131.0)	0.0	—
Folate, mcg_DFE	2.0 (0.0–7.0)	2.0 (0.0–8.0)	0.0	—
Vitamin C, mg	0.0 (0.0–0.9)	0.3 (0.0–1.2)	0.0	—
Vitamin D, mcg	0.0	0.0	0.0	—
Vitamin E, mg	0.2 (0.0–2.1)	0.1 (0.0–1.0)	14.3 (1.4–17.5)	<0.001
Vitamin K, mcg	1.0 (0.0–6.4)	0.6 (0.0–2.9)	13.6 (5.4–60.2)	<0.001
Choline, mg	8.8 (0.3–18.8)	13.3 (0.6–19.2)	0.2 (0.2–0.4)	<0.001
Processed foods (N)	325	277	48	
Vitamin A, mcg_RAE,	12.0 (0.0–53.0)	13.0 (0.0–61.0)	2.0 (0.0-–27.5)	0.04
Folate, mcg_DFE	12.0 (6.0–36.0)	11.0 (5.0–24.0)	37.5 (21.0–56.0)	<0.001
Vitamin C, mg	0.4 (0.0–3.1)	0.2 (0.0–3.5)	1.1 (0.0–3.0)	0.05
Vitamin D, mcg	0 (0.0–0.4)	0.0 (0.0–0.5)	0.0	0.60
Vitamin E, mg	0.4 (0.2–1.0)	0.4 (0.2–0.9)	1.1 (0.4–6.9)	<0.001
Vitamin K, mcg	1.8 (0.2–5.4)	1.6 (0.2–4.6)	3.0 (0.2–11.2)	0.16
Choline, mg	18.3 (10.6–50.7)	17.7 (10.2–43.4)	35.9 (17.5–53.6)	0.05
Ultraprocessed foods (N)	2169	2123	46	
Vitamin A, mcg_RAE,	13.0 (0.0–58.0)	14.0 (0.0–58.0)	0.0 (0.0–4.0)	0.12
Folate, mcg_DFE	32.0 (6.0–86.0)	32.0 (6.0–87.0)	30.0 (9.0–53.0)	0.47
Vitamin C, mg	0.3 (0.0–1.9)	0.3 (0.0–1.9)	0.1 (0.0–5.2)	0.16
Vitamin D, mcg	0.0 (0.0–0.2)	0.0 (0.0–0.2)	0.0	0.99
Vitamin E, mg	0.5 (0.2–1.3)	0.5 (0.2–1.3)	0.5 (0.2–1.5)	0.22
Vitamin K, mcg	3.1 (0.8–7.6)	3.1 (0.8–7.6)	1.6 (0.3–6.5)	0.95
Choline, mg	17.5 (8.7–30.1)	17.4 (8.8–30.1)	20.6 (4.3–27.1)	0.16
Beverages (N)	422	324	98	
Vitamin A, mcg_RAE,	0.0 (0.0–31.0)	0.0 (0.0–37.5)	0.0 (0.0–15.0)	<0.0001
Folate, mcg_DFE	1.0 (0.0–6.0)	1.0 (0.0–4.0)	4.0 (0.0–18.0)	0.04
Vitamin C, mg	0.1 (0.0–9.5)	0.0 (0.0–5.8)	5.5 (0.0–23.9)	<0.001
Vitamin D, mcg	0.0	0.0 (0.0–0.2)	0.0	<0.0001
Vitamin E, mg	0.0 (0.0–0.2)	0.0 (0.0–0.1)	0.0 (0.0–0.2)	0.16
Vitamin K, mcg	0.0 (0.0–0.4)	0.0 (0.0–0.2)	0.1 (0.0–1.2)	0.04
Choline, mg	1.6 (0.0–8.0)	0.9 (0.0–8.1)	3.3 (0.4–7.2)	0.10
Vegetables (N)	525	212	313	
Vitamin A, mcg_RAE,	42.0 (12.0–172.0)	38.5 (5.5–93.0)	47.0 (14.0–229.0)	0.16
Folate, mcg_DFE	27.0 (15.0–51.0)	18.0 (9.0–31.5)	34.0 (18.0–56.0)	<0.0001
Vitamin C, mg	10.0 (5.1–20.4)	9.1 (5.7–12.3)	12.2 (5.0–30.0)	<0.0001
Vitamin D, mcg	0.0	0.0 (0.0–0.1)	0.0	0.06
Vitamin E, mg	0.6 (0.3–1.2)	0.8 (0.4–1.5)	0.5 (0.3–1.0)	<0.01
Vitamin K, mcg	16.2 (4.8–44.7)	9.8 (3.5–26.7)	25.1 (6.7–74.3)	<0.0001
Choline, mg	14.7 (11.0–21.2)	14.9 (13.2–21.3)	14.4 (9.3–20.8)	0.03
Fruit (N)	110	43	67	
Vitamin A, mcg_RAE,	5.0 (2.0–28.0)	7.0 (2.0–27.0)	4.0 (2.0–32.0)	0.96
Folate, mcg_DFE	10.0 (4.0–19.0)	5.0 (2.0–17.0)	13.0 (6.0–19.0)	<0.001
Vitamin C, mg	8.9 (3.1–26.7)	6.3 (1.7–21.8)	10.2 (4.1–30.0)	0.02
Vitamin D, mcg	0.0	0.0	0.0	-
Vitamin E, mg	0.2 (0.1–0.6)	0.2 (0.1–0.4)	0.2 (0.1–0.7)	0.97
Vitamin K, mcg	2.6 (0.7–5.0)	1.6 (0.5–3.3)	3.0 (2.0–6.9)	0.06
Choline, mg	6.6 (4.5–9.8)	4.5 (3.8–9.2)	7.6 (5.7–10.1)	0.04
Mixed dishes (N)	1510	1441	69	
Vitamin A, mcg_RAE,	35.5 (11.0–73.0)	36.0 (11.0–72.0)	20.0 (6.0–80.0)	0.67
Folate, mcg_DFE	40.0 (16.0–66.0)	42.0 (16.0–66.0)	18.0 (14.0–30.0)	<0.001
Vitamin C, mg	1.1 (0.2–4.1)	1.0 (0.2–4.0)	3.6 (0.7–10.2)	<0.0001
Vitamin D, mcg	0.1 (0.0–0.4)	0.1 (0.0–0.4)	0.0 (0.0–0.2)	0.35
Vitamin E, mg	0.6 (0.3–1.0)	0.6 (0.3–1.0)	0.7 (0.4–1.2)	<0.01
Vitamin K, mcg	5.9 (3.1–11.3)	5.9 (3.0–11.2)	6.5 (3.9–18.0)	<0.001
Choline, mg	24.8 (14.7–41.8)	25.1 (15.0–41.9)	14.8 (9.0–34.5)	<0.0001

Abbreviations: FDA, Food and Drug Administration; FNDDS, Food and Nutrient Database for Dietary Studies; g, grams; mg, milligrams; mcg, microgram; RAE, retinol activity equivalent; DFE, dietary folate equivalent.

1 *P* values from t-tests comparing mean log-transformed nutrient values between FDA-aligned and FDA-unaligned groups.

**TABLE 6:** Median minerals per 100 g of FDA-aligned and FDA-unaligned foods and beverages consumed in the United States NHANES/FNDDS 2017–2018, overall and stratified by Nova and Food Category.

	Overall median (IQR)	FDA-unaligned median (IQR)	FDA-aligned median (IQR)	*P* value^1^
Overall (N)	4949	4214	735	
Calcium, mg	36.0 (14.0–100.0)	38.0 (14.0–104.0)	27.0 (12.0–62.0)	<0.0001
Magnesium, mg	20.0 (13.0–30.0)	20.0 (13.0–29.0)	21.0 (12.0–35.0)	<0.0001
Iron, mg	1.1 (0.5–2.0)	1.1 (0.5–2.0)	0.6 (0.3–1.6)	<0.0001
Potassium, mg	187 (120.0–274.0)	183.5 (115.0–262.0)	222.0 (142.0–330.0)	<0.0001
Sodium, mg	328 (147.0–467.0)	361 (204.0–07.0)	127.0 (8.0–190.0)	<0.0001
Minimally processed foods (N)	2257	1628	629	
Calcium, mg	25.0 (13.0–61.0)	25.0 (13.0–65.0)	27.0 (12.0–54.0)	0.35
Magnesium, mg	20.0 (13.0–27.0)	20.0 (14.0–25.0)	21.0 (12.0–33.0)	0.01
Iron, mg	0.9 (0.5–1.5)	1.0 (0.5–1.5)	0.6 (0.3–1.5)	<0.0001
Potassium, mg	207.0 (136.0–294.0)	203.0 (131.5–285.0)	222.0 (147.0–319.0)	<0.0001
Sodium, mg	253.0 (136.0–383.0)	337.0 (210.0–405.0)	123.0 (7.0–171.0)	<0.0001
Processed culinary ingredients (N)	52	43	9	
Calcium, mg	15.0 (1.0–78.0)	22.0 (5.0–91.0)	0.0 (0.0–0.0)	0.88
Magnesium, mg	2.5 (0.0–10.5)	5.0 (1.0–13.0)	0.0 (0.0–0.0)	–
Iron, mg	0.1 (0.0–0.4)	0.1 (0.1–0.5)	0.0 (0.0–0.2)	0.63
Potassium, mg	46.5 (1.5–134.5)	64.0 (21.0–153.0)	0.0 (0.0–0.0)	0.37
Sodium, mg	40.0 (2.0–174.5)	61.0 (4.0–243.0)	0.0 (0.0–0.0)	0.42
Processed foods (N)	325	277	48	
Calcium, mg	32.0 (11.0–121.0)	31.0 (11.0–123.0)	48.5 (22.0–83.0)	0.84
Magnesium, mg	22.0 (12.0–36.0)	20.0 (11.0–33.0)	35.5 (14.5–178.0)	<0.001
Iron, mg	0.9 (0.3–1.7)	0.9 (0.3–1.5)	1.6 (0.7–2.8)	<0.001
Potassium, mg	184.0 (116.0–341.0)	180.0 (108.0–309.0)	378.5 (141.5–632.5)	<0.001
Sodium, mg	361.0 (169.0–800.0)	414.0 (185.0–949.0)	202.0 (129.5–259.0)	0.03
Ultraprocessed foods (N)	2169	2123	46	
Calcium, mg	52.0 (16.0–126.0)	52.0 (16.0–126.0)	58.0 (11.0–138.0)	0.98
Magnesium, mg	21.0 (12.0–37.0)	21.0 (12.0–36.0)	53.5 (11.0–131.0)	<0.001
Iron, mg	1.4 (0.5–2.5)	1.4 (0.5–2.5)	1.5 (0.2–2.7)	0.96
Potassium, mg	179.0 (110.0–251.0)	177.0 (109.0–250.0)	251.5 (131.0–381.0)	0.42
Sodium, mg	391.0 (180.0–576.0)	394.0 (188.0–583.0)	162.5 (14.0–381.0)	<0.0001
Beverages (N)	422	324	98	
Calcium, mg	8.0 (2.0–60.0)	7.0 (3.0–62.5)	11.0 (2.0–42.0)	0.62
Magnesium, mg	5.0 (1.0–14.0)	4.0 (1.0–15.0)	7.5 (3.0–14.0)	0.62
Iron, mg	0.1 (0.0–0.3)	0.1 (0.0–0.3)	0.1 (0.0–0.3)	0.50
Potassium, mg	49.0 (11.0–138.0)	33.5 (8.0–116.5)	105.0 (37.0–180.0)	<0.0001
Sodium, mg	10.0 (4.0–39.0)	14.0 (5.0–47.0)	4.5 (2.0–19.0)	<0.0001
Vegetables (N)	525	212	313	
Calcium, mg	27.0 (16.0–45.0)	25.0 (13.5–54.5)	28.0 (18.0–44.0)	0.35
Magnesium, mg	21.0 (13.0–27.0)	22.0 (16.0–26.0)	20.0 (13.0–28.0)	0.60
Iron, mg	0.6 (0.4–1.0)	0.6 (0.3–1.0)	0.6 (0.5–1.1)	0.02
Potassium, mg	260.0 (177.0–363.0)	308.5 (212.5–405.0)	242.0 (168.0–318.0)	<0.001
Sodium, mg	165.0 (131.0–229.0)	246.0 (178.0–336.0)	139.0 (113.0–176.0)	<0.0001
Fruits (N)	110	43	67	
Calcium, mg	13.0 (7.0–25.0)	11.0 (5.0–18.0)	15.0 (9.0–33.0)	0.02
Magnesium, mg	11.0 (8.0–22.0)	8.0 (5.0–20.0)	12.0 (10.0–23.0)	<0.01
Iron, mg	0.3 (0.2–0.5)	0.3 (0.2–0.4)	0.4 (0.2–0.7)	0.16
Potassium, mg	155.0 (116.0–222.0)	123.0 (87.0–184.0)	168.0 (135.0–290.0)	<0.001
Sodium, mg	3.0 (1.0–7.0)	4.0 (3.0–7.0)	2.0 (1.0–9.0)	0.04
Mixed dishes (N)	1510	1441	69	
Calcium, mg	51.5 (18.0–124.0)	53.0 (19.0–129.0)	18.0 (9.0–46.0)	<0.0001
Magnesium, mg	19.0 (14.0–24.0)	18.0 (14.0–23.0)	32.0 (21.0–36.0)	<0.0001
Iron, mg	1.3 (0.8–1.8)	1.3 (0.8–1.9)	0.6 (0.5–1.3)	<0.0001
Potassium, mg	182.0 (131.0–232.0)	182.0 (131.0–232.0)	196.0 (126.0–241.0)	0.11
Sodium, mg	369.0 (290.0–493.0)	378.0 (302.0–503.0)	197.0 (164.0–257.0)	<0.0001

Abbreviations: FDA, Food and Drug Administration; FNDDS, Food and Nutrient Database for Dietary Studies; mg, milligrams.

1 *P* values from t-tests comparing mean log-transformed nutrient values between FDA-aligned and FDA-unaligned groups.

FDA-aligned beverages were lower in energy, added sugar (0 g vs 4.4 g, *P* < 0.0001), saturated fat, vitamin A, vitamin D, and sodium and higher in potassium (105 mg vs 33.5 g, *P* < 0.0001), compared to FDA-unaligned beverages. Compared to FDA-unaligned vegetables, FDA-aligned vegetables were lower in energy, saturated fat, and sodium and higher in fiber, folate [34.0 mcg dietary folate equivalent (DFE) vs 18.0 mcg DFE, *P* < 0.0001], vitamin C, and vitamin K (25.1 mcg vs 9.8 mcg, *P* < 0.0001). FDA-aligned fruits were not significantly different from FDA-unaligned fruits for any nutrients. Lastly, compared to FDA-unaligned mixed dishes, FDA-aligned mixed dishes were lower in energy (114.0 kcal vs 170.0 kcal, *P* < 0.0001), protein (6.0 g vs 9.0 g, *P* < 0.0001), saturated fat (0.7 g vs 2.5 g, *P* < 0.0001), monounsaturated fat (1.1 g vs 2.7 g, *P* < 0.0001), choline (14.8 mg vs 25.1 mg, *P* < 0.0001), calcium (18.0 mg vs 53.0 mg, *P* < 0.0001), iron (0.6 mg vs 1.3 mg, *P* < 0.0001), and sodium (197.0 mg vs 378.0 mg, *P* < 0.0001) and higher in fiber (1.8 g vs 1.1 g, *P* < 0.0001), vitamin C (3.6 mg vs 1.0 mg, *P* < 0.0001), and magnesium (32.0 mg vs 18.0 mg, *P* < 0.0001). Macronutrients, vitamins, and minerals for the remaining Nova categories and food categories are presented in Supplemental Tables 3–8, as are sensitivity analyses with median nutrients presented per 100 kcal (Supplemental Tables 9–14) and per RACC (Supplemental Tables 15–20).

## Discussion

In this study, we evaluated the convergent validity of FDA’s updated “healthy” claim, with nutrient profiling models and Nova, among foods and beverages in FNDDS. In exploratory analyses, we compared the nutritional profile of foods and beverages meeting “healthy” criteria to those that did not. Less than 15% of items met the “healthy” criteria, which excluded nearly all UPFs. Overall correlations with nutrient profiling models and Nova were moderate, yet correlations varied substantially by food category, with higher correlations generally observed among nuts and seeds and fats and oils, and lower correlations generally among savory snacks and desserts; meat, poultry, and eggs; and seafood. Additionally, “healthy” foods tended to be lower in saturated fat and sodium and higher in fiber and vitamin C overall and across most food categories and Nova categories.

This analysis was conducted using FNDDS, a database used to analyze NHANES dietary intake data. Given that NHANES has consistently shown that United States adults have poor dietary quality [30,37], our finding that < 15% of items qualified for the “healthy” claim is not surprising. Additionally, although FNDDS is not representative of all foods and beverages in the food supply, these findings are consistent with prior literature demonstrating misalignment between dietary recommendations and the availability of healthy products in the food supply [[38], [39], [40]]. Although it is encouraging that the majority of fruits, vegetables, and nuts and seeds in FNDDS met FDA’s “healthy” criteria and would be considered foods that could form the foundation of a healthy dietary pattern, there were few grains, dairy, and seafood that met these criteria. This is concerning as whole grains, dairy, and seafood are recommended by the DGA yet are underconsumed by United States adults. Thus, further research is needed to understand whether this finding is driven by poor availability of “healthy” grains, dairy, and seafood in the food supply, or whether “healthy” products are available but either not consumed or reported in NHANES in a way that meets the criteria.

Moderate correlations between the “healthy” criteria and other nutrient profiling models provide evidence of convergent validity for the “healthy” claim. These findings also align with prior research demonstrating moderate correlations between Food Compass 2.0, Nutri-Score, and Health Star Rating [14]. Given that different nutrient profiling models are estimated using different food characteristics (e.g., nutrients, additives, food groups) and units (e.g., serving size, per 100 g, or per 100 kcal), perfect correlation should not be expected. Nevertheless, correlations varied substantially by food category. For example, for both Food Compass 2.0 and Health Star Rating, correlations were highest for fruits and lowest for meat, poultry, and eggs. Low correlation for meat, poultry, and eggs is unsurprising, given that only eggs and game meats may qualify for the “healthy” claim in this category, whereas other nutrient profiling models may allow domestic meat and poultry to have higher scores if they are low in saturated fat and not classified as red meat (i.e., Food Compass 2.0 penalizes red meat).

Correlations were also inconsistent for a given food category. For beverages, there were strong correlations between “healthy” criteria and Food Compass 2.0 and Nova, but weaker correlations with Health Star Rating and Nutri-Score. Beverages qualifying for the “healthy” claim largely comprised minimally processed foods, including juices, smoothies, tea, coffee, and water, and most of these items were categorized as “Foods to Encourage” or “Foods to Moderate” by Food Compass 2.0. However, Health Star Rating and Nutri-Score tended to assign lower scores to juices (i.e., <3.0, and “E” or “D”), which likely explains weaker correlations between these nutrient profiling models and the “healthy” criteria among beverages.

Discrepancies between “healthy” criteria and nutrient profiling models have important implications for the specific foods and beverages that could be recommended to consumers as healthful choices. For example, “baked beans, reduced sodium” does not qualify for the “healthy” claim but would be considered a “Food to Encourage” using Food Compass 2.0. Meanwhile, “Apple juice, 100%” qualifies for the “healthy” claim, but receives a “D” using Nutri-Score. Given this and the inconsistent correlations observed across food categories, further research is needed to better inform national and global food policy efforts. Specifically, comparative criterion validity studies should build on these findings by 1) evaluating dietary intakes using different nutrient profiling models, 2) determining whether any model more effectively predicts lower risk of diet-related chronic diseases, and 3) examining whether effects vary across populations with diverse dietary patterns.

Using updated “healthy” criteria, only 2.1% of UPFs qualify for the claim. Similarly, prior studies have shown that the majority of UPFs have unfavorable nutritional profiles, which are at least partially responsible for the associations with poor health outcomes [21,41,42]. However, given that several studies have demonstrated protective associations between some UPF subgroups (e.g., dairy products, whole grain breads) [[43], [44], [45]] and health outcomes, it is plausible that reformulated “healthy” UPFs could be part of a recommended dietary pattern. Thus, investigating the health impacts of UPFs that provide key food groups (e.g., whole grains) and are low in added sugar, sodium, and saturated fat should be a top priority for future research [21].

In exploratory analyses comparing the nutrient content of “healthy” foods to foods not qualifying for the claim, we found that “healthy” foods were generally lower in saturated fat and sodium across food categories, but were not as consistently lower in added sugar. Although FDA-aligned beverages and minimally processed foods were lower in added sugar, other categories of FDA-aligned items (e.g., fruits, vegetables, mixed dishes) were not lower in added sugar, which was likely due to the wide range in the added sugar content of FDA-unaligned items in these categories. Additionally, “healthy” foods were consistently higher in fiber and vitamin C. However, there were some nutrients that were lower among “healthy” foods. For example, iron and folate are nutrients of public health interest, given that iron deficiency affects 14% of United States adults [46], and folate-fortified foods have proven critical for preventing neural tube defects [47]. Yet, “healthy” mixed dishes were lower in these nutrients compared with mixed dishes that did not meet “healthy” criteria.

Because FNDDS is not representative of the United States food supply, further research is needed to understand the availability of products in the marketplace qualifying for the “healthy” claim, as well as to assess the feasibility of using “healthy” foods as the foundation of a nutritionally adequate diet that promotes health and prevents disease. For example, in FNDDS, 86% of “healthy” foods were minimally processed foods. Given that diets primarily comprising minimally processed foods require more food preparation time than diets comprising UPF [48], future research should examine differences in the cost, availability, and meal preparation time required for a diet *founded* on foods qualifying for the “healthy” claim.

Lastly, foods that do not qualify for the “healthy” claim may still form the foundation of a healthy dietary pattern. For example, white rice does not qualify for the claim but can be the foundation of a “healthy” dietary pattern in some cultures because of the way it is consumed in tandem with seafood, vegetables, legumes, and other food groups [49,50]. Although FDA’s final rule asserts that “healthy” criteria include affordable, accessible, and culturally preferred foods across food categories [13], the small number of items in FNDDS that qualify as “healthy,” particularly in some food categories (e.g., dairy; seafood; and legumes), raises questions about the extent to which this goal can be achieved. For this reason, future research should explore whether more lenient criteria could identify a greater diversity of “healthy” foods that may still serve as the foundation of a healthy dietary pattern.

### Limitations

There are several limitations of this research. First, the nutritional profile and ingredients of packaged products in the United States food environment are continuously evolving. FNDDS is not representative of all foods and beverages in the marketplace, as it primarily includes foods as they are consumed rather than as they are purchased. Additionally, the nutritional profile for many items in FNDDS represents a composite of several other items. Accordingly, it is likely there are products (e.g., savory snacks and ready-to-heat/ready-to-eat items) that could qualify for the “healthy” claim that are not represented in FNDDS, or that do not qualify for the “healthy” claim because their nutritional profile in FNDDS is represented as a composite with other products. Therefore, generalizability to the United States marketplace may be limited, and this study should be replicated using datasets with up-to-date nutritional information for a larger range of packaged foods and beverages available in the United States marketplaces.

Additionally, a limitation of comparing FDA criteria with existing nutrient profiling models is that FDA criteria were only designed to identify “healthy” foods, whereas other nutrient profiling models rank foods across a spectrum of healthfulness. In fact, FDA criteria do not provide a binary “healthy” vs “unhealthy” classification, as FDA-unaligned foods are heterogeneous, with widely varying amounts of nutrients to limit and food groups to encourage. Given this, moderate correlations between FDA criteria and existing nutrient profiling models could be due to inherent differences in the goals of these systems. There is also the possibility of misclassifying foods according to Nova, due to the limited number of branded products, lack of ingredient lists for packaged foods, and lack of clarity in FNDDS regarding whether some mixed dishes are prepared from scratch ingredients or a ready-to-heat/ready-to-eat meal. Lastly, this study only examined the nutritional profile of individual foods and beverages in FNDDS, not dietary intake. Therefore, findings should not be generalized to draw conclusions about dietary intake of “healthy” foods in the United States. To more fully understand whether FDA “healthy” criteria can support efforts to promote health and prevent disease, it will be necessary to examine the associations between intake of “healthy” foods and dietary quality (i.e., Healthy Eating Index scores) and health outcomes.

In conclusion, <15% of foods and beverages meet FDA’s “healthy” criteria. There was evidence of convergent validity, with moderate correlations observed between “healthy” criteria and other nutrient profiling models. “Healthy” foods were also generally lower in sodium and saturated fat and higher in fiber and vitamin C compared with foods that do not qualify for the claim. Although few UPFs qualify for the “healthy” claim, industry reformulation could change this, highlighting the urgent need for research to understand whether UPFs should be excluded from the “healthy” claim. Finally, with no gold standard for assessing the healthfulness of individual foods and beverages, there is a need for validation studies to compare nutrient profiling models and determine if one is best for improving dietary quality and lowering risk of diet-related chronic disease among consumers in the United States and globally.

## Author contributions

The authors’ responsibilities were as follows – ACT designed the research and analyzed data; and ACT, LEC, CMR, and JAW wrote the article. ACT had primary responsibility for final content; all authors read and approved the final manuscript.

## Data availability

Data described in the manuscript, code book, and analytic code will be made available upon request.

## Funding

ACT was supported by Grant Number T32 HL007024 from the National Heart, Lung, and Blood Institute, National Institutes of Health, and by Grant Number T32DK062707 from the National Institute of Diabetes and Digestive and Kidney Diseases. The content is solely the responsibility of the authors and does not necessarily represent the official views of the National Institutes of Health. The funder had no role in the design, execution, or interpretation of the research, and did not have any restrictions regarding publication.

## Conflict of interest

ACT reports financial support was provided by National Heart, Lung, and Blood Institute, National Institutes of Health. The other authors report no conflict of interest.
