# Supplementary material for: What is “healthy” food? A cross-sectional evaluation of foods and beverages consumed by United States adults that satisfy the United States Food and Drug Administration’s updated “healthy” claim criteria
Source: Am J Clin Nutr. 2025 Nov 25;123(2):101121. doi: 10.1016/j.ajcnut.2025.11.011 (PMC12842601; doi:10.1016/j.ajcnut.2025.11.011)
Supplement: Multimedia component 1 [file mmc1.docx]

**Supplemental Figure 1.** Flowchart of 2017–2018 Food and Nutrient Database for Dietary Studies Foods and Beverages Included in Analytic Sample

Foods and beverages in the 2017 – 2018 FNDDS

(n = 7058)

Eligible foods and beverages included in the analytic sample (n=4949)

Human milk and infant foods and beverages not eligible for the FDA “healthy” claim

(n=204)

Foods and beverages not reported by any participants in the 2017 – 2018 NHANES wave

(n=1905)

Foods and beverages reported by at least one participant in the 2017 – 2018 NHANES wave (n=5153)

(n = 14,976)

**Supplemental Table 1. Nova Food Processing Classification Definitions and Examples** ^a^

| **Nova Classification** | **Definition** | **Examples** |
| --- | --- | --- |
| Group 1: Unprocessed or minimally processed foods | Includes foods that have undergone no processing or have only undergone minimal processing. Minimal processing includes grinding, roasting, pasteurization, freezing, drying, fermentation, roasting, or boiling. | - Fruits and vegetables, including fresh, frozen, dried, and juice forms - Grains, including whole and refined forms that have been subjected to grinding or parboiling - Legumes - Fungi - Meat, poultry, eggs, and seafood - Dairy, including pasteurized or fermented forms - Nuts and seeds - Herbs and spices - Tea, coffee, water |
| Group 2: Processed culinary ingredients | Includes substances extracted, pressed, refined, or centrifuged from Group 1 foods or other foods occurring in nature. They are used to  season and cook homemade or artisanal dishes from Group 1 foods. | - Table salt - Sugar, including table sugar, honey, and molasses - Oils from crushed seeds, nuts, or fruits - Animal fats, including butter and lard - Vinegar |
| Group 3: Processed foods | Foods that are manufactured using unprocessed or minimally processed foods (Group 1) with the addition of Group 2 processed culinary ingredients. This prolongs the durability of foods and modifies their palatability. Preservatives and stabilizers may be used in the preparation of these foods. | - Cheese, freshly made - Canned fruits and vegetables preserved in brine or syrup - Manufactured bread prepared from wheat flour and Group 2 ingredients (ie. salt, yeast, butter, sugar) - Salted, smoked, canned, or cured meat or fish - Nuts and seeds with added salt and/or sugar |
| Group 4: Ultra-processed foods | Foods that are formulations of several ingredients including Group 2 ingredients, and also food additives not used in culinary preparations. Additives include flavors, colors, sweeteners, emulsifiers, and other substances used to disguise undesirable qualities of the final product or imitate the sensorial qualities of Group 1 culinary preparations. | - Carbonated soft drinks, including diet versions - Mass-produced breads - Sweet or savory packaged snacks - Chocolate, pastries, cakes, and confectionary - Many ready-to-eat and ready-to-eat heat foods such as frozen or shelf-stable dishes, pies, pasta dishes, pizza, sandwiches, burgers - Plant-based meat alternatives - Reconstituted meat products such as chicken nuggets, sausage, hot dogs, and deli meats |

1. Adapted from: Monteiro, C. A., Cannon, G., Levy, R. B., Moubarac, J. C., Louzada, M. L., Rauber, F., Khandpur, N., Cediel, G., Neri, D., Martinez-Steele, E., Baraldi, L. G., & Jaime, P. C. (2019). Ultra-processed foods: what they are and how to identify them. Public health nutrition, 22(5), 936–941. https://doi.org/10.1017/S136898001800376

**Supplemental Table 2.** Item Descriptions and Nova Subcategories of FDA-Aligned Ultra-Processed Foods (n=46)

| **Food Category and Item Description** | **Nova Sub-Category** |
| --- | --- |
| Beverages |  |
| Carbonated water, sweetened, with low-calorie or no-calorie sweetener | 38 - Soft drinks, carbonated |
| Carbonated water, unsweetened | 38 - Soft drinks, carbonated |
| Fruit juice drink, citrus, carbonated | 38 - Soft drinks, carbonated |
| Fruit juice drink, noncitrus, carbonated | 38 - Soft drinks, carbonated |
| Fruit smoothie, bottled | 39 - Other sweetened beverages |
| Fruit smoothie juice drink, with dairy | 25 - Ice cream, ice pops and frozen yogurts |
| Fruit smoothie, with whole fruit, no dairy, added protein ^a^ | 39 - Other sweetened beverages |
| Fruit smoothie, with whole fruit, non-dairy ^a^ | 39 - Other sweetened beverages |
| Fruit and vegetable smoothie, bottled | 39 - Other sweetened beverages |
| Nutritional powder mix (EAS Whey Protein Powder) | 39 - Other sweetened beverages |
| Nutritional powder mix, protein, NFS | 39 - Other sweetened beverages |
| Nutritional powder mix, whey based, NFS | 39 - Other sweetened beverages |
| Tea, iced, bottled, black, diet | 39 - Other sweetened beverages |
| Tea, iced, bottled, black, decaffeinated, diet | 39 - Other sweetened beverages |
| Tea, iced, brewed, green, decaffeinated, pre-sweetened with low calorie sweetener | 39 - Other sweetened beverages |
| Water, bottled, flavored, sugar free (Glaceau Vitamin Water) | 39 - Other sweetened beverages |
| Water, bottled, flavored, sugar free (SoBe) | 39 - Other sweetened beverages |
| Water, bottled, sweetened, with low calorie sweetener | 39 - Other sweetened beverages |
| Grains |  |
| Bread, gluten free | 23 - Breads |
| Bread, multigrain | 23 - Breads |
| Bread, multigrain, toasted | 23 – Breads |
| Bread, multigrain, with raisins | 23 – Breads |
| Bread, whole wheat | 23 – Breads |
| Breadsticks, hard, gluten free | 23 – Breads |
| Cereal (General Mills Fiber One) | 27 - Breakfast cereals |
| Cereal (Kashi 7 Whole Grain Puffs) | 27 - Breakfast cereals |
| Cereal, muesli | 27 - Breakfast cereals |
| Cereal (Post Shredded Wheat) | 27 - Breakfast cereals |
| Cereal (Uncle Sam) | 27 - Breakfast cereals |
| Muffin, English, whole grain white | 23 – Breads |
| Oatmeal, instant, plain, made with non-dairy milk, no added fat ^a^ | 37 – Milk-based drinks |
| Mixed Dishes ^a^ |  |
| Egg substitute, omelet, scrambled, or fried, with vegetables | 30 - Frozen and shelf-stable plate meals |
| Fish sandwich, from school cafeteria | 23 - Breads |
| Pasta with sauce and meat, from school lunch | 30 - Frozen and shelf-stable plate meals |
| Pasta with sauce, meatless, school lunch | 30 - Frozen and shelf-stable plate meals |
| Peanut butter sandwich, with reduced fat peanut butter, on wheat bread | 23 - Breads |
| Peanut butter sandwich, with reduced fat peanut butter, on whole wheat bread | 23 – Breads |
| Savory Snacks |  |
| Chips, rice | 28 - Salty-snacks |
| Crackers, crispbread | 28 - Salty-snacks |
| Crackers, gluten free, flavored | 28 - Salty-snacks |
| Crackers, gluten free, plain | 28 - Salty-snacks |
| Popcorn cake | 28 - Salty-snacks |
| Rice cake | 28 - Salty-snacks |
| Vegetable chips | 28 - Salty-snacks |
| Vegetable mixture, dried | 28 - Salty-snacks |
| Vegetables |  |
| Potato, hash brown, from school lunch | 33 - French fries and other potato products |

1. Items consisting of more than one Nova group were categorized as an ultra-processed food if >50% of weight in grams came from ultra-processed foods.

**Supplemental Table 3.** Mean Macronutrients per 100g of FDA-Aligned and FDA-Unaligned Foods and Beverages Consumed in the United States NHANES/FNDDS 2017-2018, Overall and Stratified by Nova^1^

|  | **Overall**  **Median (IQR)** | **FDA Unaligned**  **Median (IQR)** | **FDA Aligned**  **Median (IQR)** | **p-value^2^** |
| --- | --- | --- | --- | --- |
| Overall (N) | 4949 | 4214 | 735 |  |
| Energy, kcal | 182.0 (96.0-297.0) | 202.0 (120.0-308.0) | 63.0 (41.0-135.0) | <0.001 |
| Protein, g | 6.4 (2.4-11.9) | 7.1 (3.2-12.5) | 2.4 (1.1-6.3) | <0.001 |
| Carbohydrate, g | 15.1 (5.6-30.3) | 16.5 (6.0-34.4) | 9.5 (4.6-18.5) | <0.001 |
| Sugars, added, g | 0.0 (0.0-3.9) | 0.4 (0.0-5.8) | 0.0 (0.0-0.0) | <0.001 |
| Fiber, total dietary, g | 1.1 (0.2-2.4) | 1.0 (0.1-2.2) | 2.0 (1.0-3.8) | <0.001 |
| Saturated fat, g | 1.6 (0.5-4.0) | 2.0 (0.7-4.3) | 0.5 (0.0-0.9) | <0.001 |
| Monounsaturated fat, g | 2.1 (0.5-4.7) | 2.5 (0.8-5.0) | 0.6 (0.0-1.2) | <0.001 |
| Polyunsaturated fat, g | 1.1 (0.3-2.8) | 1.3 (0.4-3.0) | 0.5 (0.1-1.3) | <0.001 |
| Minimally Processed Foods (N) | 2257 | 1628 | 629 |  |
| Energy, kcal | 133.0 (76.0-190.0) | 154.0 (115.0-203.5) | 58.0 (40.0-113.0) | <0.001 |
| Protein, g | 5.9 (2.3-13.9) | 8.1 (3.5-18.7) | 2.2 (1.1-4.3) | <0.001 |
| Carbohydrate, g | 10.0 (3.6-18.1) | 10.9(1.6-18.4) | 9.0 (4.6-16.0) | 0.13 |
| Sugars, added, g | 0.0 (0.0-0.0) | 0.0 (0.0-0.5) | 0.0 (0.0-0.0) | <0.001 |
| Fiber, total dietary, g | 1.0 (0.2-1.9) | 0.7 (0.0-1.4) | 2.0 (1.1-3.4) | <0.001 |
| Saturated fat, g | 1.2 (0.5-2.7) | 1.7 (0.9-3.3) | 0.4 (0.0-0.8) | <0.001 |
| Monounsaturated fat, g | 1.6 (0.5-3.4) | 2.2 (1.0-4.1) | 0.3 (0.0-1.1) | <0.001 |
| Polyunsaturated fat, g | 1.0 (0.3-2.2) | 1.3 (0.5-2.6) | 0.4 (0.1-1.1) | <0.001 |
| Processed Culinary Ingredients (N) | 52 | 43 | 9 |  |
| Energy, kcal | 323.5 (188.0-717.0) | 292.0 (176.0-474.0) | 884.0 (884.0-884.0) | <0.01 |
| Protein, g | 0.7 (0.0-3.1) | 0.9 (0.1-3.6) | 0.0 (0.0-0.0) | - |
| Carbohydrate, g | 10.6 (0.2-66.1) | 23.5 (3.4-75.0) | 0.0 (0.0-0.0) | - |
| Sugars, added, g | 0.0 (0.0-40.3) | 0.0 (0.0-68.0) | 0.0 (0.0-0.0) | - |
| Fiber, total dietary, g | 0.0 (0.0-0.2) | 0.0 (0.0-0.2) | 0.0 (0.0-0.0) | - |
| Saturated fat, g | 7.0 (0.0-16.6) | 4.6 (0.0-21.2) | 10.3 (8.2-13.7) | 0.99 |
| Monounsaturated fat, g | 3.3 (0.0-19.7) | 2.3 (0.0-7.8) | 39.7 (22.8-63.3) | 0.03 |
| Polyunsaturated fat, g | 0.5 (0.0-3.2) | 0.2 (0.0-1.7) | 41.2 (17.4-54.7) | <0.001 |
| Processed Foods (N) | 325 | 277 | 48 |  |
| Energy, kcal | 173 (84.0-329.0) | 170.0 (84.0-299.0) | 182.0 (68.0-605.5) | 0.05 |
| Protein, g | 8.7 (1.5-21.0) | 8.7 (1.3-21.6) | 8.7 (2.2-20.3) | 0.61 |
| Carbohydrate, g | 7.9 (2.6-18.8) | 5.9 (2.1-16.3) | 14.3 (8.6-21.1) | <0.01 |
| Sugars, added, g | 0.0 (0.0-1.1) | 0.0 (0.0-1.6) | 0.0 (0.0-0.0) | 0.03 |
| Fiber, total dietary, g | 0.8 (0.0-2.6) | 0.4 (0.0-2.2) | 4.9 (1.8-9.3) | <0.001 |
| Saturated fat, g | 1.6 (0.1-6.3) | 1.6 (0.1-5.8) | 2.2 (0.6-6.9) | 0.52 |
| Monounsaturated fat, g | 2.1 (0.1-7.3) | 2.1 (0.1-6.0) | 2.6 (0.6-28.5) | 0.02 |
| Polyunsaturated fat, g | 0.9 (0.2-2.7) | 0.8 (0.1-2.3) | 1.5 (0.5-13.6) | <0.001 |
| Ultra-Processed Foods (N) | 2169 | 2123 | 46 |  |
| Energy, kcal | 274.0 (136.0-379.0) | 275.0 (138.0-379.0) | 236.0 (53.0-353.0) | 0.01 |
| Protein, g | 6.5 (3.0-10.8) | 6.5 (3.0-10.8) | 7.5 (0.6-12.4) | 0.32 |
| Carbohydrate, g | 27.5 (11.8-55.9) | 27.5 (11.9-55.9) | 29.0 (6.9-65.8_ | 0.47 |
| Sugars, added, g | 2.6 (0.0-14.0) | 2.7 (0.0-14.4) | 0.0 (0.0-1.0) | 0.01 |
| Fiber, total dietary, g | 1.5 (0.3-2.9) | 1.4 (0.3-2.9) | 3.2 (0.3-7.7) | <0.001 |
| Saturated fat, g | 2.2 (0.6-5.0) | 2.3 (0.6-5.1) | 0.5 (0.1-0.9) | <0.001 |
| Monounsaturated fat, g | 2.6 (0.6-5.6) | 2.7 (0.6-5.7) | 0.5 (0.0-1.6) | <0.001 |
| Polyunsaturated fat, g | 1.4 (0.4-3.5) | 1.4 (0.4-3.5) | 0.9 (0.1-1.9) | 0.23 |

1. Abbreviations: kilocalorie (kcal), grams (g), interquartile range (IQR)
2. P-values from t-tests comparing mean log-transformed nutrient values between FDA-aligned and FDA-unaligned groups

**Supplemental Table 4.** Mean Macronutrients per 100g of FDA-Aligned and FDA-Unaligned Foods and Beverages Consumed in the United States NHANES/FNDDS 2017-2018, Overall and Stratified by Food Category^1^

|  | **Overall**  **Median (IQR)** | **FDA Unaligned**  **Median (IQR)** | **FDA Aligned**  **Median (IQR)** | **p-value^2^** |
| --- | --- | --- | --- | --- |
| Beverages (N) | 422 | 324 | 98 |  |
| Energy, kcal | 46.0 (21.0-84.0) | 50.5 (26.5-90.0) | 38.5 (1.0-53.0) | <0.001 |
| Protein, g | 0.2 (0.0-1.2) | 0.2 (0.0-1.5) | 0.4 (0.1-0.7) | 0.63 |
| Carbohydrate, g | 7.7 (1.9-12.7) | 7.7 (2.6-13.1) | 7.7 (0.2-12.2) | 0.32 |
| Sugars, added, g | 0.4 (0.0-7.7) | 4.4 (0.0-8.7) | 0.0 (0.0-0.0) | <0.001 |
| Fiber, total dietary, g | 0.0 (0.0-0.2) | 0.0 (0.0-0.1) | 0.2 (0.0-0.8) | 0.10 |
| Saturated fat, g | 0.0 (0.0-0.1) | 0.0 (0.0-0.2) | 0.0 (0.0-0.0) | <0.001 |
| Monounsaturated fat, g | 0.0 (0.0-0.1) | 0.0 (0.0-0.2) | 0.0 (0.0-0.0) | <0.001 |
| Polyunsaturated fat, g | 0.0 (0.0-0.1) | 0.0 (0.0-0.1) | 0.0 (0.0-0.1) | <0.01 |
| Grains (N) | 437 | 416 | 21 |  |
| Energy, kcal | 279.0 (140.0-370.0) | 285.0 (147.0-372.0) | 223.0 (68.0-291.0) | <0.01 |
| Protein, g | 6.9 (4.0-9.5) | 6.9 (4.1-9.5) | 6.6 (2.2-12.4) | 0.13 |
| Carbohydrate, g | 48.3 (27.2-76.1) | 48.9 (28.0-77.5) | 43.3 (13.2-65.8) | 0.02 |
| Sugars, added, g | 3.6 (0.0-16.8) | 4.0 (0.2-17.3) | 0.0 (0.0-2.8) | 0.15 |
| Fiber, total dietary, g | 2.7 (1.7-5.8) | 2.6 (1.7-5.7) | 4.6 (1.9-8.0) | 0.12 |
| Saturated fat, g | 0.8 (0.4-1.5) | 0.8 (0.4-1.5) | 0.4 (0.2-0.9) | <0.01 |
| Monounsaturated fat, g | 0.8 (0.4-1.9) | 0.9 (0.4-2.0) | 0.6 (0.3-0.8) | 0.01 |
| Polyunsaturated fat, g | 0.9 (0.5-1.8) | 0.9 (0.5-1.8) | 0.8 (0.4-1.6) | 0.15 |
| Vegetables (N) | 525 | 212 | 313 |  |
| Energy, kcal | 68.0 (43.0-123.0) | 125.0 (93.0-191.5) | 50.0 (35.0-74.0) | <0.001 |
| Protein, g | 2.1 (1.4-3.1) | 2.3 (1.9-3.8) | 1.9 (1.3-3.0) | <0.001 |
| Carbohydrate, g | 9.7 (5.0-18.4) | 17.8 (12.0-20.9) | 6.7 (4.4-11.6) | <0.001 |
| Sugars, added, g | 0.0 (0.0-0.0) | 0.0 (0.0-0.0) | 0.0 (0.0-0.0) | 0.11 |
| Fiber, total dietary, g | 2.1 (1.5-2.9) | 1.8 (1.4-2.4) | 2.5 (1.7-3.2) | <0.001 |
| Saturated fat, g | 0.8 (0.1-1.2) | 1.4 (0.9-2.5) | 0.5 (0.1-0.8) | <0.001 |
| Monounsaturated fat, g | 1.0 (0.1-1.5) | 1.6 (1.0-3.9) | 0.9 (0.0-1.0) | <0.001 |
| Polyunsaturated fat, g | 0.9 (0.2-1.3 | 1.3 (0.5-4.0) | 0.8 (0.1-1.0) | <0.001 |
| Fruits (N) | 110 | 43 | 67 |  |
| Energy, kcal | 60.5 (48.0-150.0) | 70.0 (53.0-159.0) | 57.0 (43.0-126.0) | 0.09 |
| Protein, g | 0.8 (0.5-1.4) | 0.6 (0.4-1.3) | 0.9 (0.7-1.5) | <0.01 |
| Carbohydrate, g | 15.0 (11.9-22.8) | 16.2 (13.5-26.1) | 13.9 (11.1-19.2) | 0.43 |
| Sugars, added, g | 0.0 (0.0-3.3) | 5.3 (1.6-8.0) | 0.0 (0.0-0.0) | 0.13 |
| Fiber, total dietary, g | 2.1 (1.4-3.6) | 1.5 (1.0-2.4) | 2.4 (1.6-4.5) | <0.001 |
| Saturated fat, g | 0.0 (0.0-0.1) | 0.0 (0.0-0.6) | 0.0 (0.0-0.1) | 0.20 |
| Monounsaturated fat, g | 0.0 (0.0-0.1) | 0.0 (0.0-0.4) | 0.0 (0.0-0.1) | 0.26 |
| Polyunsaturated fat, g | 0.1 (0.0-0.2) | 0.1 (0.0-0.2) | 0.1 (0.1-0.2) | 0.79 |
| Legumes (N) | 80 | 45 | 35 |  |
| Energy, kcal | 176.5 (139.5-191.0) | 177.0 (137.0-196.0) | 176.0 (155.0-188.0) | 0.43 |
| Protein, g | 8.4 (7.5-9.4) | 8.4 (7.4-10.7) | 8.4 (8.1-8.9) | 0.46 |
| Carbohydrate, g | 21.6 (18.7-24.3) | 20.6 (14.3-23.6) | 23.3 (19.4-25.4) | 0.01 |
| Sugars, added, g | 0.0 (0.0-0.0) | 0.0 (0.0-0.0) | 0.0 (0.0-0.0) | - |
| Fiber, total dietary, g | 7.1 (5.0-8.1) | 6.4 (4.3-7.7) | 7.3 (6.3-8.4) | 0.02 |
| Saturated fat, g | 1.0 (0.4-1.2) | 1.1 (0.5-1.4) | 1.0 (0.3-1.0) | 0.10 |
| Monounsaturated fat, g | 2.8 (0.6-2.8) | 2.3 (0.6-3.0) | 2.8 (0.6-2.8) | 0.80 |
| Polyunsaturated fat, g | 2.8 (0.5-2.9) | 2.6 (0.5-3.6) | 2.8 (0.5-2.9) | 0.79 |
| Nuts and Seeds (N) | 77 | 24 | 53 |  |
| Energy, kcal | 587.0 (574.0-609.0) | 569.5 (485.0-597.5) | 599.0 (579.0-614.0) | <0.01 |
| Protein, g | 19.3 (13.7-21.0) | 13.8 (10.8-21.5) | 19.6 (15.2-21.0) | 0.03 |
| Carbohydrate, g | 21.3 (18.8-30.3) | 30.6 (21.8-43.2) | 21.2 (15.3-24.1) | <0.001 |
| Sugars, added, g | 0.0 (0.0-2.9) | 6.4 (3.3-21.8) | 0.0 (0.0-0.0) | <0.01 |
| Fiber, total dietary, g | 7.8 (6.1-9.9) | 6.1 (4.8-8.1) | 8.4 (7.5-10.0) | <0.01 |
| Saturated fat, g | 7.0 (5.2-8.5) | 6.8 (5.0-10.1) | 7.0 (5.6-7.7) | 0.46 |
| Monounsaturated fat, g | 25.9 (15.7-28.4) | 17.6 (11.8-25.7) | 27.0 (23.9-30.0) | 0.02 |
| Polyunsaturated fat, g | 13.6 (9.5-19.6) | 12.4 (8.0-14.1) | 14.0 (12.3-21.6) | 0.04 |
| Meat (N) | 472 | 458 | 14 |  |
| Energy, kcal | 211.0 (175.0-269.0) | 213.0 (176.0-269.0) | 151.5 (143.0-190.0) | <0.0001 |
| Protein, g | 23.6 (18.6-27.2) | 23.6 (18.9-27.1) | 12.8 (12.5-28.3) | 0.01 |
| Carbohydrate, g | 0.1 (0.0-4.3) | 0.1 (0.0-4.6) | 0.7 (0.0-0.9) | 0.20 |
| Sugars, added, g | 0.0 (0.0-0.0) | 0.0 (0.0-0.0) | 0.0 (0.0-0.0) | - |
| Fiber, total dietary, g | 0.0 (0.0-0.0) | 0.0 (0.0-0.0) | 0.0 (0.0-0.0) | - |
| Saturated fat, g | 3.1 (2.2-5.2) | 3.1 (2.2-5.3) | 2.7 (2.0-3.1) | 0.41 |
| Monounsaturated fat, g | 4.8 (2.9-7.5) | 4.8 (2.9-7.5) | 3.2 (0.9-4.0) | <0.01 |
| Polyunsaturated fat, g | 2.0 (0.9-3.3) | 2.0 (1.0-3.4) | 1.4 (0.3-1.9) | <0.05 |
| Seafood (N) | 193 | 176 | 17 |  |
| Energy, kcal | 176.0 (127.0-218.0) | 180.5 (134.5-219.0) | 127.0 (91.0-143.0) | <0.001 |
| Protein, g | 19.9 (17.4-23.3) | 20.3 (17.7-23.7) | 17.5 (16.1-19.0) | 0.03 |
| Carbohydrate, g | 1.2 (0.1-10.2) | 0.2 (0.1-10.5) | 2.7 (0.1-4.4) | 0.50 |
| Sugars, added, g | 0.0 (0.0-0.5) | 0.0 (0.0-0.5) | 0.0 (0.0-0.0) | 0.95 |
| Fiber, total dietary, g | 0.0 (0.0-0.6) | 0.0 (0.0-0.6) | 0.0 (0.0-0.0) | 0.04 |
| Saturated fat, g | 1.4 (0.7-2.2) | 1.5 (0.8-2.2) | 0.7 (0.3-0.9) | <0.001 |
| Monounsaturated fat, g | 2.2 (0.8-4.0) | 2.3 (0.9-4.1) | 1.3 (0.2-1.7) | 0.01 |
| Polyunsaturated fat, g | 1.8 (0.8-3.4) | 1.9 (0.9-3.6) | 0.8 (0.4-1.7) | 0.02 |
| Dairy (N) | 196 | 174 | 22 |  |
| Energy, kcal | 95.0 (71.0-303.0) | 105.5 (73.0-316.0) | 58.5 (43.0-73.0) | <0.001 |
| Protein, g | 5.1 (3.2-16.1) | 4.9 (3.2-16.4) | 5.3 (3.4-10.0) | 0.73 |
| Carbohydrate, g | 8.5 (4.4-12.1) | 9.1 (4.4-12.3) | 5.2 (4.6-7.0) | 0.31 |
| Sugars, added, g | 0.0 (0.0-5.0) | 0.0 (0.0-5.3) | 0.0 (0.0-0.0) | - |
| Fiber, total dietary, g | 0.0 (0.0-0.0) | 0.0 (0.0-0.0) | 0.0 (0.0-0.0) |  |
| Saturated fat, g | 1.7 (0.8-11.4) | 1.9 (1.0-12.9) | 0.6 (0.1-1.0) | <0.001 |
| Monounsaturated fat, g | 0.7 (0.3-5.2) | 0.9 (0.4-6.0) | 0.2 (0.0-0.4) | <0.001 |
| Polyunsaturated fat, g | 0.1 (0.0-0.7) | 0.2 (0.1-0.7) | 0.0 (0.0-0.0) | <0.001 |
| Fats and Oils (N) | 68 | 60 | 8 |  |
| Energy, kcal | 430.0 (215.5-717.0) | 401.5 (201.0-537.5) | 884.0 (884.0-885.0) | <0.001 |
| Protein, g | 0.7 (0.3-1.2) | 0.9 (0.4-1.3) | 0.0 (0.0-0.0) | - |
| Carbohydrate, g | 5.3 (0.0-16.2) | 7.2 (0.8-18.7) | 0.0 (0.0-0.0) | - |
| Sugars, added, g | 3.0 (0.0-9.2) | 3.6 (0.0-10.4) | 0.0 (0.0-0.0) | - |
| Fiber, total dietary, g | 0.0 (0.0-0.3) | 0.0 (0.0-0.4) | 0.0 (0.0-0.0) |  |
| Saturated fat, g | 7.2 (2.5-13.3) | 6.1 (2.1-12.5) | 11.6 (8.7-13.7) | 0.22 |
| Monounsaturated fat, g | 10.4 (4.6-19.9) | 8.8 (4.1-19.3) | 40.8 (25.2-66.6) | <0.001 |
| Polyunsaturated fat, g | 10.7 (3.1-25.4) | 9.5 (2.9-22.9) | 41.4 (22.8-59.0) | 0.01 |
| Mixed Dishes (N) | 1510 | 1441 | 69 |  |
| Energy, kcal | 165.0 (117.0-245.0) | 170.0 (121.0-247.0) | 114.0 (97.0-123.0) | <0.0001 |
| Protein, g | 8.8 (5.4-12.2) | 9.0 (5.5-12.2) | 6.0 (2.7-9.2) | <0.0001 |
| Carbohydrate, g | 16.4 (7.7-22.7) | 16.4 (7.7-22.8) | 18.5 (5.3-21.9) | <0.19 |
| Sugars, added, g | 0.0 (0.0-1.6) | 0.0 (0.0-1.7) | 0.0 (0.0-0.0) | 0.30 |
| Fiber, total dietary, g | 1.1 (0.7-1.8) | 1.1 (0.7-1.8) | 1.8 (1.3-2.7) | <0.0001 |
| Saturated fat, g | 2.3 (1.0-4.2) | 2.5 (1.0-4.3) | 0.7 (0.4-1.0) | <0.0001 |
| Monounsaturated fat, g | 2.6 (1.3-4.4) | 2.7 (1.4-4.4) | 1.1 (0.9-2.4) | <0.0001 |
| Polyunsaturated fat, g | 1.4 (0.7-2.7) | 1.5 (0.8-2.7) | 1.1 (0.5-1.9) | 0.10 |
| Sauce and Condiments (N) | 178 | 169 | 9 |  |
| Energy, kcal | 147.0 (53.0-278.0) | 151.0 (56.0-280.0) | 25.0 (22.0-34.0) | <0.001 |
| Protein, g | 1.2 (0.5-3.7) | 1.2 (0.6-3.7) | 0.4 (0.4-1.4) | 0.07 |
| Carbohydrate, g | 10.8 (5.6-44.1) | 11.2 (5.3-45.6) | 7.7 (6.9-8.4) | 0.28 |
| Sugars, added, g | 0.0 (0.0-18.9) | 1.0 (0.0-26.0) | 0.0 (0.0-0.0) | - |
| Fiber, total dietary, g | 0.8 (0.2-1.8) | 0.8 (0.1-1.8) | 0.4 (0.4-2.1) | 0.58 |
| Saturated fat, g | 0.1 (0.0-2.4) | 0.1 (0.0-2.5) | 0.0 (0.0-0.1) | 0.01 |
| Monounsaturated fat, g | 0.1 (0.0-3.7) | 0.1 (0.0-3.7) | 0.0 (0.0-0.0) | <0.001 |
| Polyunsaturated fat, g | 0.1 (0.0-1.4) | 0.2 (0.0-1.4) | 0.0 (0.0-0.2) | 0.01 |
| Snack and Desserts (N) | 680 | 671 | 9 |  |
| Energy, kcal | 411.5 (331.5-471.5) | 412.0 (330.0-473.0) | 392.0 (384.0-453.0) | 0.32 |
| Protein, g | 5.4 (3.8-8.0) | 5.3 (3.7-8.0) | 9.7 (7.9-11.3) | 0.01 |
| Carbohydrate, g | 58.4 (43.5-69.7) | 58.1 (43.3-69.4) | 77.5 (66.3-81.1) | 0.02 |
| Sugars, added, g | 18.6 (3.3-32.7) | 19.0 (5.0-33.0) | 0.0 (0.0-0.0) | - |
| Fiber, total dietary, g | 2.2 (1.1-3.8) | 2.1 (1.0-3.8) | 10.2 (4.2-10.2) | <0.001 |
| Saturated fat, g | 4.0 (2.1-7.1) | 4.1 (2.1-7.3) | 0.9 (0.6-2.1) | <0.001 |
| Monounsaturated fat, g | 4.8 (2.4-7.8) | 4.8 (2.4-7.9) | 1.6 (0.9-7.0) | 0.14 |
| Polyunsaturated fat, g | 3.1 (1.0-5.5) | 3.1 (1.0-5.4) | 2.3 (1.4-6.0) | 0.81 |

1. Abbreviations: kilocalorie (kcal), grams (g), interquartile range (IQR)
2. P-values from t-tests comparing mean log-transformed nutrient values between FDA-aligned and FDA-unaligned groups

**Supplemental Table 5.** Mean Vitamins per 100g of FDA-Aligned and FDA-Unaligned Foods and Beverages Consumed in the United States NHANES/FNDDS 2017-2018, Overall and Stratified by Nova^1^

|  | **Overall**  **Median (IQR)** | **FDA Unaligned**  **Median (IQR)** | **FDA Aligned**  **Median (IQR)** | **p-value^2^** |
| --- | --- | --- | --- | --- |
| Overall (N) | 4949 | 4214 | 735 |  |
| Vitamin A, mcg_RAE, | 18.0 (1.0-62.0) | 18.0 (1.0-62.0) | 14.0 (1.0-64.0) | 0.63 |
| Thiamin, mg | 0.1 (0.0-0.2) | 0.1 (0.0-0.2) | 0.1 (0.0-0.1) | <0.001 |
| Riboflavin, mg | 0.1 (0.1-0.2) | 0.2 (0.1-0.2) | 0.1 (0.0-0.1) | <0.001 |
| Niacin, mg | 1.5 (0.5-3.6) | 1.8 (0.6-3.9) | 0.6 (0.3-1.2) | <0.001 |
| Vitamin B-6, mg | 0.1 (0.1-0.2) | 0.1 (0.0-0.2) | 0.1 (0.1-0.2) | 0.03 |
| Folate, mcg_DFE | 23.0 (7.0-61.0) | 23.0 (7.0-63.0) | 22.0 (10.0-53.0) | 0.87 |
| Vitamin B-12, mcg | 0.1 (0.0-0.5) | 0.2 (0.0-0.6) | 0.0 (0.0-0.0) | 0.16 |
| Vitamin C, mg | 0.5 (0.0-4.3) | 0.4 (0.0-2.9) | 5.1 (0.8-18.8) | <0.001 |
| Vitamin D, mcg | 0 (0.0-0.3) | 0.1 (0.0-0.4) | 0.0 (0.0-0.0) | 0.08 |
| Vitamin E, mg | 0.5 (0.2-1.1) | 0.5 (0.2-1.2) | 0.5 (0.2-1.1) | 0.24 |
| Vitamin K, mcg | 3.6 (0.8-9.7) | 3.4 (0.7-8.6) | 6.5 (1.1-25.6) | <0.001 |
| Choline, mg | 19.5 (9.9-41.3) | 20.8 (10.8-44.3) | 14.4 (7.1-27.1) | <0.001 |
| Minimally Processed Foods (N) | 2257 | 1628 | 629 |  |
| Vitamin A, mcg_RAE, | 20.0 (4.0-65.0) | 20.0 (5.0-64.0) | 19.0 (1.0-80.0) | <0.01 |
| Thiamin, mg | 0.1 (0.0-0.1) | 0.1 (0.1-0.2) | 0.1 (0.0-0.1) | <0.001 |
| Riboflavin, mg | 0.1 (0.1-0.2) | 0.1 (0.1-0.2) | 0.1 (0.0-0.1) | <0.001 |
| Niacin, mg | 1.3 (0.6-2.8) | 1.7 (0.9-3.7) | 0.6 (0.3-1.1) | <0.001 |
| Vitamin B-6, mg | 0.1 (0.1-0.2) | 0.2 (0.1-0.3) | 0.1 (0.1-0.2) | <0.001 |
| Folate, mcg_DFE | 21.0 (8.0-48.0) | 20.5 (8.0-46.0) | 21.0 (10.0-51.0) | <0.001 |
| Vitamin B-12, mcg | 0.1 (0.0-0.6) | 0.3 (0.1-0.7) | 0.0 (0.0-0.0) | 0.66 |
| Vitamin C, mg | 1.3 (0.0-7.4) | 0.5 (0.0-4.1) | 7.0 (1.1-22.4) | <0.001 |
| Vitamin D, mcg | 0.0 (0.0-0.3) | 0.1 (0.0-0.5) | 0.0 (0.0-0.0) | 0.13 |
| Vitamin E, mg | 0.5 (0.2-1.0) | 0.5 (0.3-1.0) | 0.5 (0.2-1.0) | 0.34 |
| Vitamin K, mcg | 4.6 (0.8-12.3) | 4.1 (0.8-10.0) | 7.3 (1.7-28.8) | <0.001 |
| Choline, mg | 22.6 (10.7-61.2) | 32.0 (13.8-72.8) | 13.4 (7.1-24.6) | <0.001 |
| Processed Culinary Ingredients (N) | 52 | 43 | 9 |  |
| Vitamin A, mcg_RAE, | 0.0 (0.0-119.0) | 8.0 (0.0-131.0) | 0.0 (0.0-0.0) | - |
| Thiamin, mg | 0.0 (0.0-0.0) | 0.0 (0.0-0.0) | 0.0 (0.0-0.0) | - |
| Riboflavin, mg | 0.0 (0.0-0.2) | 0.0 (0.0-0.2) | 0.0 (0.0-0.0) | - |
| Niacin, mg | 0.1 (0.0-0.2) | 0.1 (0.0-0.2) | 0.0 (0.0-0.0) | - |
| Vitamin B-6, mg | 0.0 (0.0-0.1) | 0.0 (0.0-0.1) | 0.0 (0.0-0.0) | - |
| Folate, mcg_DFE | 2.0 (0.0-7.0) | 2.0 (0.0-8.0) | 0.0 (0.0-0.0) | - |
| Vitamin B-12, mcg | 0.0 (0.0-0.2) | 0.1 (0.0-0.2) | 0.0 (0.0-0.0) | - |
| Vitamin C, mg | 0.0 (0.0-0.9) | 0.3 (0.0-1.2) | 0.0 (0.0-0.0) | - |
| Vitamin D, mcg | 0.0 (0.0-0.0) | 0.0 (0.0-0.0) | 0.0 (0.0-0.0) | - |
| Vitamin E, mg | 0.2 (0.0-2.1) | 0.1 (0.0-1.0) | 14.3 (1.4-17.5) | <0.001 |
| Vitamin K, mcg | 1.0 (0.0-6.4) | 0.6 (0.0-2.9) | 13.6 (5.4-60.2) | <0.001 |
| Choline, mg | 8.8 (0.3-18.8) | 13.3 (0.6-19.2) | 0.2 (0.2-0.4) | <0.001 |
| Processed Foods (N) | 325 | 277 | 48 |  |
| Vitamin A, mcg_RAE, | 12.0 (0.0-53.0) | 13.0 (0.0-61.0) | 2.0 (0.0-27.5) | 0.04 |
| Thiamin, mg | 0.1 (0.0-0.2) | 0.0 (0.0-0.2) | 0.1 (0.0-0.2) | 0.11 |
| Riboflavin, mg | 0.1 (0.1-0.2) | 0.1 (0.1-0.2) | 0.1 (0.1-0.3) | 0.18 |
| Niacin, mg | 0.8 (0.2-3.0) | 0.7 (0.1-2.4) | 1.0 (0.7-3.3) | 0.01 |
| Vitamin B-6, mg | 0.1 (0.1-0.2) | 0.1 (0.1-0.2) | 0.1 (0.1-0.3) | 0.04 |
| Folate, mcg_DFE | 12.0 (6.0-36.0) | 11.0 (5.0-24.0) | 37.5 (21.0-56.0) | <0.001 |
| Vitamin B-12, mcg | 0.0 (0.0-0.9) | 0.1 (0.0-1.1) | 0.0 (0.0-0.0) | 0.27 |
| Vitamin C, mg | 0.4 (0.0-3.1) | 0.2 (0.0-3.5) | 1.1 (0.0-3.0) | 0.05 |
| Vitamin D, mcg | 0 (0.0-0.4) | 0.0 (0.0-0.5) | 0.0 (0.0-0.0) | 0.60 |
| Vitamin E, mg | 0.4 (0.2-1.0) | 0.4 (0.2-0.9) | 1.1 (0.4-6.9) | <0.001 |
| Vitamin K, mcg | 1.8 (0.2-5.4) | 1.6 (0.2-4.6) | 3.0 (0.2-11.2) | 0.16 |
| Choline, mg | 18.3 (10.6-50.7) | 17.7 (10.2-43.4) | 35.9 (17.5-53.6) | 0.05 |
| Ultra-Processed Foods (N) | 2169 | 2123 | 46 |  |
| Vitamin A, mcg_RAE, | 13.0 (0.0-58.0) | 14.0 (0.0-58.0) | 0.0 (0.0-4.0) | 0.12 |
| Thiamin, mg | 0.2 (0.0-0.3) | 0.2 (0.0-0.3) | 0.1 (0.0-0.4) | 0.41 |
| Riboflavin, mg | 0.2 (0.1-0.3) | 0.2 (0.1-0.3) | 0.1 (0.0-0.2) | 0.66 |
| Niacin, mg | 2.2 (0.4-4.2) | 2.2 (0.4-4.2) | 3.3 (0.3-4.8) | 0.25 |
| Vitamin B-6, mg | 0.1 (0.0-0.2) | 0.1 (0.0-0.2) | 0.2 (0.1-0.4) | 0.01 |
| Folate, mcg_DFE | 32.0 (6.0-86.0) | 32.0 (6.0-87.0) | 30.0 (9.0-53.0) | 0.47 |
| Vitamin B-12, mcg | 0.2 (0.0-0.5) | 0.2 (0.0-0.5) | 0.0 (0.0-0.2) | 0.04 |
| Vitamin C, mg | 0.3 (0.0-1.9) | 0.3 (0.0-1.9) | 0.1 (0.0-5.2) | 0.16 |
| Vitamin D, mcg | 0.0 (0.0-0.2) | 0.0 (0.0-0.2) | 0.0 (0.0-0.0) | 0.99 |
| Vitamin E, mg | 0.5 (0.2-1.3) | 0.5 (0.2-1.3) | 0.5 (0.2-1.5) | 0.22 |
| Vitamin K, mcg | 3.1 (0.8-7.6) | 3.1 (0.8-7.6) | 1.6 (0.3-6.5) | 0.95 |
| Choline, mg | 17.5 (8.7-30.1) | 17.4 (8.8-30.1) | 20.6 (4.3-27.1) | 0.16 |

1. Abbreviations: grams (g), milligrams (mg), microgram (mcg), interquartile range (IQR), retinol activity equivalent (RAE), dietary folate equivalent (DFE)
2. P-values from t-tests comparing mean log-transformed nutrient values between FDA-aligned and FDA-unaligned groups

**Supplemental Table 6.** Mean Vitamins per 100g of FDA-Aligned and FDA-Unaligned Foods and Beverages Consumed in the United States NHANES/FNDDS 2017-2018, Overall and Stratified by Food Category^1^

|  | **Overall** | **FDA Unaligned** | **FDA Aligned** | **p-value^2^** |
| --- | --- | --- | --- | --- |
| Beverages (N) | 422 | 324 | 98 |  |
| Vitamin A, mcg_RAE, | 0.0 (0.0-31.0) | 0.0 (0.0-37.5) | 0.0 (0.0-15.0) | <0.001 |
| Thiamin, mg | 0.0 (0.0-0.0) | 0.0 (0.0-0.0) | 0.0 (0.0-0.0) | 0.02 |
| Riboflavin, mg | 0.0 (0.0-0.1) | 0.0 (0.0-0.1) | 0.0 (0.0-0.1) | 0.13 |
| Niacin, mg | 0.2 (0.0-0.8) | 0.1 (0.0-0.9) | 0.2 (0.0-0.4) | 0.95 |
| Vitamin B-6, mg | 0.0 (0.0-0.1) | 0.0 (0.0-0.0) | 0.0 (0.0-0.1) | 0.98 |
| Folate, mcg_DFE | 1.0 (0.0-6.0) | 1.0 (0.0-4.0) | 4.0 (0.0-18.0) | 0.04 |
| Vitamin B-12, mcg | 0.0 (0.0-0.2) | 0.0 (0.0-0.3) | 0.0 (0.0-0.0) | 0.79 |
| Vitamin C, mg | 0.1 (0.0-9.5) | 0.0 (0.0-5.8) | 5.5 (0.0-23.9) | <0.001 |
| Vitamin D, mcg | 0.0 (0.0-0.0) | 0.0 (0.0-0.2) | 0.0 (0.0-0.0) | <0.001 |
| Vitamin E, mg | 0.0 (0.0-0.2) | 0.0 (0.0-0.1) | 0.0 (0.0-0.2) | 0.16 |
| Vitamin K, mcg | 0.0 (0.0-0.4) | 0.0 (0.0-0.2) | 0.1 (0.0-1.2) | 0.04 |
| Choline, mg | 1.6 (0.0-8.0) | 0.9 (0.0-8.1) | 3.3 (0.4-7.2) | 0.10 |
| Grains (N) | 437 | 416 | 21 |  |
| Vitamin A, mcg_RAE, | 17.0 (0.0-199.0) | 19.0 (0.0-209.5) | 0.0 (0.0-0.0) | 0.82 |
| Thiamin, mg | 0.4 (0.2-0.7) | 0.4 (0.2-0.7) | 0.2 (0.1-0.4) | <0.01 |
| Riboflavin, mg | 0.3 (0.1-0.7) | 0.3 (0.1-0.7) | 0.1 (0.0-0.2 | <0.01 |
| Niacin, mg | 3.7 (1.5-8.6) | 3.8 (1.6-9.1) | 2.8 (0.3-4.4) | <0.001 |
| Vitamin B-6, mg | 0.1 (0.1-0.9) | 0.1 (0.1-0.9) | 0.1 (0.0-0.3) | 0.03 |
| Folate, mcg_DFE | 108.0 (40.0-334.0) | 114.5 (43.5-487.0) | 32.0 (6.0-68.0) | <0.001 |
| Vitamin B-12, mcg | 0.0 (0.0-0.5) | 0.1 (0.0-2.3) | 0.0 (0.0-0.0) | 0.44 |
| Vitamin C, mg | 0.0 (0.0-1.3) | 0.0 (0.0-1.3) | 0.0 (0.0-0.1) | 0.04 |
| Vitamin D, mcg | 0.0 (0.0-0.9) | 0.0 (0.0-1.1) | 0.0 (0.0-0.0) | 0.71 |
| Vitamin E, mg | 0.4 (0.2-0.6) | 0.4 (0.2-0.7) | 0.3 (0.1-0.5) | 0.39 |
| Vitamin K, mcg | 1.5 (0.6-3.4) | 1.5 (0.7-3.6) | 0.8 (0.3-1.6) | 0.03 |
| Choline, mg | 13.7 (7.5-20.5) | 13.7 (7.5-20.0) | 19.0 (6.8-23.1) | 0.22 |
| Vegetables (N) | 525 | 212 | 313 |  |
| Vitamin A, mcg_RAE, | 42.0 (12.0-172.0) | 38.5 (5.5-93.0) | 47.0 (14.0-229.0) | 0.16 |
| Thiamin, mg | 0.1 (0.0-0.1) | 0.1 (0.1-0.1) | 0.1 (0.0-0.1) | <0.001 |
| Riboflavin, mg | 0.1 (0.0-0.1) | 0.1 (0.0-0.1) | 0.1 (0.0-0.1) | 0.03 |
| Niacin, mg | 0.8 (0.5-1.3) | 1.1 (0.7-1.4) | 0.6 (0.4-1.0) | <0.001 |
| Vitamin B-6, mg | 0.1 (0.1-0.2) | 0.2 (0.1-0.3) | 0.1 (0.1-0.2) | <0.001 |
| Folate, mcg_DFE | 27.0 (15.0-51.0) | 18.0 (9.0-31.5) | 34.0 (18.0-56.0) | <0.001 |
| Vitamin B-12, mcg | 0.0 (0.0-0.0) | 0.0 (0.0-0.1) | 0.0 (0.0-0.0) | 0.99 |
| Vitamin C, mg | 10.0 (5.1-20.4) | 9.1 (5.7-12.3) | 12.2 (5.0-30.0) | <0.001 |
| Vitamin D, mcg | 0.0 (0.0-0.0) | 0.0 (0.0-0.1) | 0.0 (0.0-0.0) | 0.06 |
| Vitamin E, mg | 0.6 (0.3-1.2) | 0.8 (0.4-1.5) | 0.5 (0.3-1.0) | <0.01 |
| Vitamin K, mcg | 16.2 (4.8-44.7) | 9.8 (3.5-26.7) | 25.1 (6.7-74.3) | <0.001 |
| Choline, mg | 14.7 (11.0-21.2) | 14.9 (13.2-21.3) | 14.4 (9.3-20.8) | 0.04 |
| Fruit (N) | 110 | 43 | 67 |  |
| Vitamin A, mcg_RAE, | 5.0 (2.0-28.0) | 7.0 (2.0-27.0) | 4.0 (2.0-32.0) | 0.96 |
| Thiamin, mg | 0.0 (0.0-0.1) | 0.0 (0.0-0.1) | 0.0 (0.0-0.1) | 0.19 |
| Riboflavin, mg | 0.0 (0.0-0.1) | 0.0 (0.0-0.1) | 0.0 (0.0-0.1) | 0.03 |
| Niacin, mg | 0.4 (0.2-0.6) | 0.4 (0.1-0.5) | 0.4 (0.3-0.8) | <0.01 |
| Vitamin B-6, mg | 0.1 (0.0-0.1) | 0.0 (0.0-0.1) | 0.1 (0.0-0.1) | 0.02 |
| Folate, mcg_DFE | 10.0 (4.0-19.0) | 5.0 (2.0-17.0) | 13.0 (6.0-19.0) | <0.001 |
| Vitamin B-12, mcg | 0.0 (0.0-0.0) | 0.0 (0.0-0.0) | 0.0 (0.0-0.0) | - |
| Vitamin C, mg | 8.9 (3.1-26.7) | 6.3 (1.7-21.8) | 10.2 (4.1-30.0) | 0.02 |
| Vitamin D, mcg | 0.0 (0.0-0.0) | 0.0 (0.0-0.0) | 0.0 (0.0-0.0) | - |
| Vitamin E, mg | 0.2 (0.1-0.6) | 0.2 (0.1-0.4) | 0.2 (0.1-0.7) | 0.97 |
| Vitamin K, mcg | 2.6 (0.7-5.0) | 1.6 (0.5-3.3) | 3.0 (2.0-6.9) | 0.06 |
| Choline, mg | 6.6 (4.5-9.8) | 4.5 (3.8-9.2) | 7.6 (5.7-10.1) | 0.04 |
| Legumes (N) | 80 | 45 | 35 |  |
| Vitamin A, mcg_RAE, | 0.0 (0.0-1.0) | 0.0 (0.0-2.0) | 0.0 (0.0-1.0) | 0.02 |
| Thiamin, mg | 0.2 (0.1-0.2) | 0.1 (0.1-0.2) | 0.2 (0.1-0.2) | 0.72 |
| Riboflavin, mg | 0.1 (0.1-0.1) | 0.1 (0.1-0.2) | 0.1 (0.1-0.1) | 0.37 |
| Niacin, mg | 0.5 (0.4-0.9) | 0.6 (0.4-1.0) | 0.5 (0.3-0.6) | 0.05 |
| Vitamin B-6, mg | 0.1 (0.1-0.2) | 0.1 (0.1-0.2) | 0.1 (0.1-0.2) | 0.24 |
| Folate, mcg_DFE | 95.5 (43.0-144.0) | 56.0 (37.0-111.0) | 140.0 (73.0-157.0) | <0.001 |
| Vitamin B-12, mcg | 0.0 (0.0-0.0) | 0.0 (0.0-0.1) | 0.0 (0.0-0.0) | - |
| Vitamin C, mg | 0.6 (0.0-1.3) | 0.7 (0.0-1.7) | 0.5 (0.0-1.1) | 0.08 |
| Vitamin D, mcg | 0.0 (0.0-0.0) | 0.0 (0.0-0.0) | 0.0 (0.0-0.0) | - |
| Vitamin E, mg | 1.0 (0.7-1.6) | 1.1 (0.3-1.6) | 0.9 (0.8-1.6) | 0.32 |
| Vitamin K, mcg | 9.2 (3.3-10.9) | 5.3 (1.0-10.9) | 9.5 (4.0-10.9) | 0.11 |
| Choline, mg | 32.7 (29.9-39.8) | 34.1 (29.4-43.4) | 32.2 (29.9-34.4) | 0.93 |
| Nuts and Seeds (N) | 77 | 24 | 53 |  |
| Vitamin A, mcg_RAE, | 0.0 (0.0-1.0) | 0.0 (0.0-0.0) | 0.0 (0.0-1.0) | 0.02 |
| Thiamin, mg | 0.2 (0.1-0.3) | 0.1 (0.1-0.2) | 0.2 (0.1-0.6) | <0.001 |
| Riboflavin, mg | 0.2 (0.1-0.3) | 0.2 (0.1-0.2) | 0.2 (0.2-0.3) | 0.02 |
| Niacin, mg | 3.6 (1.4-7.5) | 5.3 (1.3-9.6) | 3.5 (1.4-7.5) | 0.64 |
| Vitamin B-6, mg | 0.3 (0.2-0.5) | 0.2 (0.1-0.4) | 0.4 (0.2-0.5) | 0.10 |
| Folate, mcg_DFE | 66.0 (51.0-97.0) | 59.5 (45.0-86.5) | 67.0 (53.0-97.0) | 0.71 |
| Vitamin B-12, mcg | 0.0 (0.0-0.0) | 0.0 (0.0-0.0) | 0.0 (0.0-0.0) | - |
| Vitamin C, mg | 0.4 (0.0-1.1) | 0.0 (0.0-0.9) | 0.6 (0.0-1.1) | 0.25 |
| Vitamin D, mcg | 0.0 (0.0-0.0) | 0.0 (0.0-0.0) | 0.0 (0.0-0.0) | - |
| Vitamin E, mg | 5.4 (1.7-9.7) | 5.4 (3.1-11.3) | 5.5 (1.4-9.7) | 0.27 |
| Vitamin K, mcg | 3.5 (0.2-11.7) | 2.7 (0.3-9.6) | 4.3 (0.0-13.2) | <0.001 |
| Choline, mg | 52.6 (40.5-61.0) | 42.3 (33.9-58.6) | 55.3 (50.3-61.0) | 0.13 |
| Meat (N) | 472 | 458 | 14 |  |
| Vitamin A, mcg_RAE, | 7.0 (1.0-14.0) | 7.0 (1.0-14.0) | 80.5 (0.0-160.0) | <0.01 |
| Thiamin, mg | 0.1 (0.1-0.3) | 0.1 (0.1-0.3) | 0.1 (0.0-0.2) | <0.01 |
| Riboflavin, mg | 0.2 (0.2-0.3) | 0.2 (0.2-0.3) | 0.5 (0.4-0.7) | <0.0001 |
| Niacin, mg | 5.7 (4.4-7.6) | 5.7 (4.5-7.6) | 0.1 (0.1-7.4) | <0.0001 |
| Vitamin B-6, mg | 0.4 (0.2-0.5) | 0.4 (0.2-0.5) | 0.2 (0.1-0.5) | <0.0001 |
| Folate, mcg_DFE | 7.0 (4.0-14.5) | 7.0 (4.0-13.0) | 31.0 (11.0-44.0) | <0.01 |
| Vitamin B-12, mcg | 0.6 (0.4-1.2) | 0.6 (0.4-1.2) | 1.2 (0.7-2.9) | 0.12 |
| Vitamin C, mg | 0.0 (0.0-0.0) | 0.0 (0.0-0.0) | 0.0 (0.0-0.0) | - |
| Vitamin D, mcg | 0.2 (0.1-0.5) | 0.2 (0.1-0.5) | 0.8 (0.0-2.0) | <0.0001 |
| Vitamin E, mg | 0.4 (0.2-0.8) | 0.4 (0.2-0.8) | 1.0 (0.8-1.1) | <0.001 |
| Vitamin K, mcg | 1.5 (0.0-4.9) | 1.6 (0.0-5.1) | 0.3 (0.3-1.5) | <0.0001 |
| Choline, mg | 74.2 (62.3-93.0) | 73.5 (62.3-92.3) | 171.8 (114.3-235.0) | 0.15 |
| Seafood (N) | 193 | 176 | 17 |  |
| Vitamin A, mcg_RAE, | 27.0 (15.0-46.0) | 27.0 (13.5-44.0) | 32.0 (16.0-83.0) | 0.28 |
| Thiamin, mg | 0.1 (0.0-0.1) | 0.1 (0.0-0.2) | 0.0 (0.0-0.1) | <0.01 |
| Riboflavin, mg | 0.1 (0.1-0.2) | 0.1 (0.1-0.2) | 0.1 (0.0-0.1) | 0.27 |
| Niacin, mg | 3.1 (1.9-5.5) | 3.2 (1.9-5.6) | 2.2 (1.1-2.5) | 0.02 |
| Vitamin B-6, mg | 0.2 (0.1-0.3) | 0.2 (0.1-0.3) | 0.1 (0.0-0.2) | <0.001 |
| Folate, mcg_DFE | 18.0 (8.0-29.0) | 20.0 (9.0-29.0) | 7.0 (5.0-22.0) | 0.03 |
| Vitamin B-12, mcg | 2.1 (1.4-3.7) | 2.0 (1.4-3.6) | 2.1 (1.3-8.6) | 0.46 |
| Vitamin C, mg | 0.5 (0.0-0.50 | 0.5 (0.0-0.5) | 0.0 (0.0-0.5) | 0.45 |
| Vitamin D, mcg | 1.7 (0.2-5.6) | 2.1 (0.3-6.3) | 0.1 (0.0-0.4) | 0.02 |
| Vitamin E, mg | 1.2 (0.8-1.8) | 1.2 (0.8-1.8) | 1.2 (0.7-1.8) | 0.51 |
| Vitamin K, mcg | 4.2 (0.4-7.5) | 4.2 (0.4-7.5) | 1.0 (0.2-4.6) | 0.34 |
| Choline, mg | 80.9 (75.0-88.5) | 80.9 (75.3-88.8) | 77.5 (65.0-83.8) | 0.38 |
| Dairy (N) | 196 | 174 | 22 |  |
| Vitamin A, mcg_RAE, | 64.0 (29.0-145.5) | 68.5 (30.0-165.0) | 58.0 (14.0-90.0) | 0.06 |
| Thiamin, mg | 0.0 (0.0-0.1) | 0.0 (0.0-0.1) | 0.0 (0.0-0.1) | 0.15 |
| Riboflavin, mg | 0.2 (0.1-0.3) | 0.2 (0.1-0.3) | 0.2 (0.1-0.2) | 0.97 |
| Niacin, mg | 0.1 (0.1-0.2) | 0.1 (0.1-0.2) | 0.1 (0.1-0.2) | 0.76 |
| Vitamin B-6, mg | 0.1 (0.0-0.1) | 0.1 (0.0-0.8) | 0.1 (0.0-0.1) | 0.86 |
| Folate, mcg_DFE | 6.5 (2.0-11.5) | 6.0 (2.0-11.0) | 8.0 (2.0-12.0) | 0.87 |
| Vitamin B-12, mcg | 0.5 (0.4-0.8) | 0.5 (0.4-0.8) | 0.6 (0.5-0.6) | 0.98 |
| Vitamin C, mg | 0.0 (0.0-0.8) | 0.0 (0.0-0.8) | 0.8 (0.0-0.8) | 0.87 |
| Vitamin D, mcg | 0.9 (0.1-1.1) | 0.6 (0.1-1.1) | 1.1 (0.1-1.2) | 0.13 |
| Vitamin E, mg | 0.1 (0.0-0.3) | 0.1 (0.0-0.3) | 0.0 (0.0-0.0) | <0.001 |
| Vitamin K, mcg | 0.3 (0.2-1.8) | 0.4 (0.2-2.1) | 0.1 (0.0-0.2) | <0.001 |
| Choline, mg | 15.6 (14.6-18.2) | 15.7 (14.4-18.2) | 15.2 (15.2-17.4) | 0.43 |
| Fats and Oils (N) | 68 | 60 | 8 |  |
| Vitamin A, mcg_RAE, | 11.0 (1.0-373.5) | 14.0 (2.0-574.0) | 0.0 (0.0-0.0) | - |
| Thiamin, mg | 0.0 (0.0-0.0) | 0.0 (0.0-0.0) | 0.0 (0.0-0.0) | - |
| Riboflavin, mg | 0.0 (0.0-0.1) | 0.0 (0.0-0.1) | 0.0 (0.0-0.0) | - |
| Niacin, mg | 0.0 (0.0-0.1) | 0.0 (0.0-0.1) | 0.0 (0.0-0.0) | - |
| Vitamin B-6, mg | 0.0 (0.0-0.0) | 0.0 (0.0-0.0) | 0.0 (0.0-0.0) | - |
| Folate, mcg_DFE | 2.0 (0.0-4.5) | 3.0 (1.0-5.0) | 0.0 (0.0-0.0) | - |
| Vitamin B-12, mcg | 0.0 (0.0-0.1) | 0.1 (0.0-0.1) | 0.0 (0.0-0.0) | - |
| Vitamin C, mg | 0.0 (0.0-0.3) | 0.0 (0.0-0.3) | 0.0 (0.0-0.0) | - |
| Vitamin D, mcg | 0.0 (0.0-0.1) | 0.0 (0.0-0.1) | 0.0 (0.0-0.0) | - |
| Vitamin E, mg | 2.3 (1.2-4.9) | 2.2 (1.0-4.3) | 14.3 (6.6-28.3) | 0.01 |
| Vitamin K, mcg | 22.1 (4.6-73.0) | 26.2 (4.2-74.6) | 14.3 (6.2-65.8) | 0.89 |
| Choline, mg | 11.6 (4.3-16.0) | 12.9 (5.8-17.2) | 0.2 (0.2-0.4) | <0.001 |
| Mixed Dishes (N) | 1510 | 1441 | 69 |  |
| Vitamin A, mcg_RAE, | 35.5 (11.0-73.0) | 36.0 (11.0-72.0) | 20.0 (6.0-80.0) | 0.67 |
| Thiamin, mg | 0.1 (0.1-0.2) | 0.1 (0.1-0.2) | 0.1 (0.0-0.2) | <0.01 |
| Riboflavin, mg | 0.1 (0.1-0.2) | 0.1 (0.1-0.2) | 0.1 (0.1-0.1) | <0.001 |
| Niacin, mg | 2.1 (1.2-3.2) | 2.1 (1.2-3.2) | 1.9 (0.8-2.2) | <0.01 |
| Vitamin B-6, mg | 0.1 (0.1-0.2) | 0.1 (0.1-0.2) | 0.1 (0.1-0.2) | 0.31 |
| Folate, mcg_DFE | 40.0 (16.0-66.0) | 42.0 (16.0-66.0) | 18.0 (14.0-30.0) | <0.001 |
| Vitamin B-12, mcg | 0.31 (0.1-0.6) | 0.3 (0.1-0.6) | 0.1 (0.0-0.3) | 0.10 |
| Vitamin C, mg | 1.1 (0.2-4.1) | 1.0 (0.2-4.0) | 3.6 (0.7-10.2) | <0.0001 |
| Vitamin D, mcg | 0.1 (0.0-0.4) | 0.1 (0.0-0.4) | 0.0 (0.0-0.0) | 0.35 |
| Vitamin E, mg | 0.6 (0.3-1.0) | 0.6 (0.3-1.0) | 0.7 (0.4-1.2) | <0.01 |
| Vitamin K, mcg | 5.9 (3.1-11.3) | 5.9 (3.0-11.2) | 6.5 (3.9-18.0) | <0.001 |
| Choline, mg | 24.8 (14.7-41.8) | 25.1 (15.0-41.9) | 14.8 (9.0-34.5) | <0.0001 |
| Sauces and Condiments (N) | 178 | 169 | 9 |  |
| Vitamin A, mcg_RAE, | 6.0 (0.0-38.0) | 8.0 (0.0-40.0) | 2.0 (0.0-2.0) | 0.01 |
| Thiamin, mg | 0.0 (0.0-0.0) | 0.0 (0.0-0.0) | 0.0 (0.0-0.1) | 0.23 |
| Riboflavin, mg | 0.1 (0.0-0.1) | 0.1 (0.0-0.1) | 0.0 (0.0-0.1) | 0.04 |
| Niacin, mg | 0.2 (0.1-0.7) | 0.2 (0.1-0.7) | 0.2 (0.1-0.7) | 0.84 |
| Vitamin B-6, mg | 0.0 (0.0-0.1) | 0.0 (0.0-0.1) | 0.0 (0.0-0.1) | 0.26 |
| Folate, mcg_DFE | 8.0 (2.0-16.0) | 7.0 (2.0-15.0) | 10.0 (9.0-20.0) | 0.39 |
| Vitamin B-12, mcg | 0.0 (0.0-0.1) | 0.0 (0.0-0.1) | 0.0 (0.0-0.0) | - |
| Vitamin C, mg | 0.9 (0.0-7.4) | 0.9 (0.0-4.5) | 30.0 (14.3-38.7) | <0.001 |
| Vitamin D, mcg | 0.0 (0.0-0.0) | 0.0 (0.0-0.0) | 0.0 (0.0-0.0) | - |
| Vitamin E, mg | 0.3 (0.0-1.4) | 0.4 (0.0-1.4) | 0.2 (0.2-0.2) | 0.33 |
| Vitamin K, mcg | 1.8 (0.2-13.0) | 2.2 (0.2-13.0) | 0.6 (0.1-1.7) | 0.13 |
| Choline, mg | 9.0 (3.1-17.3) | 9.5 (3.0-17.4) | 5.1 (5.1-9.2) | 0.57 |
| Snacks and Desserts (N) | 680 | 671 | 9 |  |
| Vitamin A, mcg_RAE, | 8.0 (0.0-48.0) | 8.0 (0.0-49.0) | 0.0 (0.0-4.0) | 0.63 |
| Thiamin, mg | 0.2 (0.1-0.3) | 0.2 (0.1-0.3) | 0.2 (0.1-0.2) | 0.96 |
| Riboflavin, mg | 0.2 (0.1-0.3) | 0.2 (0.1-0.3) | 0.2 (0.1-0.2) | 0.73 |
| Niacin, mg | 1.6 (0.6-3.4) | 1.5 (0.6-3.3) | 4.8 (2.3-6.0) | 0.01 |
| Vitamin B-6, mg | 0.1 (0.0-0.1) | 0.1 (0.0-0.1) | 0.2 (0.2-0.4) | <0.01 |
| Folate, mcg_DFE | 35.0 (15.0-85.5) | 36.0 (14.0-87.0) | 35.0 (21.0-35.0) | 0.90 |
| Vitamin B-12, mcg | 0.0 (0.0-0.2) | 0.0 (0.0-0.2) | 0.0 (0.0-0.0) | - |
| Vitamin C, mg | 0.2 (0.0-1.0) | 0.2 (0.0-1.0) | 0.0 (0.0-0.0) | - |
| Vitamin D, mcg | 0.0 (0.0-0.1) | 0.0 (0.1) | 0.0 (0.0-0.0) | - |
| Vitamin E, mg | 1.0 (0.4-2.2) | 1.0 (0.4-2.2) | 1.5 (0.8-1.5) | 0.46 |
| Vitamin K, mcg | 4.3 (1.5-9.8) | 4.2 (1.5-9.7) | 6.5 (1.9-13.7) | 0.40 |
| Choline, mg | 19.1 (10.2-29.4) | 18.7 (10.0-29.5) | 24.3 (21.1-27.1) | 0.23 |

1. Abbreviations: grams (g), milligrams (mg), microgram (mcg), interquartile range (IQR), retinol activity equivalent (RAE), dietary folate equivalent (DFE)
2. P-values from t-tests comparing mean log-transformed nutrient values between FDA-aligned and FDA-unaligned groups

**Supplemental Table 7.** Mean Minerals per 100g of FDA-Aligned and FDA-Unaligned Foods and Beverages Consumed in the United States NHANES/FNDDS 2017-2018, Overall and Stratified by Nova^1^

|  | **Overall**  **Median (IQR)** | **FDA Unaligned**  **Median (IQR)** | **FDA Aligned**  **Median (IQR)** | **p-value^2^** |
| --- | --- | --- | --- | --- |
| Overall (N) | 4949 | 4214 | 735 |  |
| Calcium, mg | 36.0 (14.0-100.0) | 38.0 (14.0-104.0) | 27.0 (12.0-62.0) | <0.001 |
| Phosphorus, mg | 106.0 (51.0-193.0) | 118.0 (62.0-198.0) | 51.0 (25.0-107.0) | <0.001 |
| Magnesium, mg | 20.0 (13.0-30.0) | 20.0 (13.0-29.0) | 21.0 (12.0-35.0) | <0.001 |
| Iron, mg | 1.1 (0.5-2.0) | 1.1 (0.5-2.0) | 0.6 (0.3-1.6) | <0.001 |
| Zinc, mg | 0.7 (0.4-1.4) | 0.8 (0.4-1.5) | 0.4 (0.2-0.8) | <0.001 |
| Copper, mg | 0.1 (0.0-0.1) | 0.1 (0.1-0.1) | 0.1 (0.0-0.2) | 0.02 |
| Selenium, mcg | 8.1 (1.7-20.0) | 10.1 (2.8-21.8) | 0.9 (0.4-4.4) | <0.001 |
| Potassium, mg | 187 (120.0-274.0) | 183.5 (115.0-262.0) | 222.0 (142.0-330.0) | <0.001 |
| Sodium, mg | 328 (147.0-467.0) | 361 (204.0-507.0) | 127.0 (8.0-190.0) | <0.001 |
| Minimally Processed Foods (N) | 2257 | 1628 | 629 |  |
| Calcium, mg | 25.0 (13.0-61.0) | 25.0 (13.0-65.0) | 27.0 (12.0-54.0) | 0.35 |
| Phosphorus, mg | 95.0 (49.0-185.0) | 115.0 (66.0-201.5) | 49.0 (25.0-95.0) | <0.001 |
| Magnesium, mg | 20.0 (13.0-27.0) | 20.0 (14.0-25.0) | 21.0 (12.0-33.0) | 0.01 |
| Iron, mg | 0.9 (0.5-1.5) | 1.0 (0.5-1.5) | 0.6 (0.3-1.5) | <0.001 |
| Zinc, mg | 0.6 (0.4-1.3) | 0.8 (0.4-1.5) | 0.4 (0.2-0.7) | <0.001 |
| Copper, mg | 0.1 (0.1-0.1) | 0.1 (0.1-0.1) | 0.1 (0.0-0.2) | 0.14 |
| Selenium, mcg | 7.5 (1.2-22.3) | 12.6 (4.8-26.1) | 0.7 (0.4-3.3) | <0.001 |
| Potassium, mg | 207.0 (136.0-294.0) | 203.0 (131.5-285.0) | 222.0 (147.0-319.0) | <0.001 |
| Sodium, mg | 253.0 (136.0-383.0) | 337.0 (210.0-405.0) | 123.0 (7.0-171.0) | <0.001 |
| Processed Culinary Ingredients (N) | 52 | 43 | 9 |  |
| Calcium, mg | 15.0 (1.0-78.0) | 22.0 (5.0-91.0) | 0.0 (0.0-0.0) | 0.88 |
| Phosphorus, mg | 12.5 (0.0-93.5) | 24.0 (4.0-95.0) | 0.0 (0.0-0.0) | - |
| Magnesium, mg | 2.5 (0.0-10.5) | 5.0 (1.0-13.0) | 0.0 (0.0-0.0) | - |
| Iron, mg | 0.1 (0.0-0.4) | 0.1 (0.1-0.5) | 0.0 (0.0-0.2) | 0.63 |
| Zinc, mg | 0.1 (0.0-0.4) | 0.2 (0.0-0.5) | 0.0 (0.0-0.0) | - |
| Copper, mg | 0.0 (0.0-0.0) | 0.0 (0.0-0.0) | 0.0 (0.0-0.0) | - |
| Selenium, mcg | 1.0 (0.1-4.0) | 1.8 (0.6-5.6) | 0.0 (0.0-0.0) | - |
| Potassium, mg | 46.5 (1.5-134.5) | 64.0 (21.0-153.0) | 0.0 (0.0-0.0) | 0.37 |
| Sodium, mg | 40.0 (2.0-174.5) | 61.0 (4.0-243.0) | 0.0 (0.0-0.0) | 0.42 |
| Processed Foods (N) | 325 | 277 | 48 |  |
| Calcium, mg | 32.0 (11.0-121.0) | 31.0 (11.0-123.0) | 48.5 (22.0-83.0) | 0.84 |
| Phosphorus, mg | 145.0 (34.0-337.0) | 142.0 (28.0-310.0) | 170.5 (45.0-397.0) | 0.17 |
| Magnesium, mg | 22.0 (12.0-36.0) | 20.0 (11.0-33.0) | 35.5 (14.5-178.0) | <0.001 |
| Iron, mg | 0.9 (0.3-1.7) | 0.9 (0.3-1.5) | 1.6 (0.7-2.8) | <0.001 |
| Zinc, mg | 0.9 (0.3-2.6) | 0.9 (0.2-2.5) | 1.0 (0.3-3.3) | 0.04 |
| Copper, mg | 0.1 (0.0-0.2) | 0.1 (0.0-0.1) | 0.2 (0.1-0.9) | <0.001 |
| Selenium, mcg | 6.1 (0.7-20.3) | 7.9 (0.7-25.1) | 1.9 (0.7-9.3) | 0.04 |
| Potassium, mg | 184.0 (116.0-341.0) | 180.0 (108.0-309.0) | 378.5 (141.5-632.5) | <0.001 |
| Sodium, mg | 361.0 (169.0-800.0) | 414.0 (185.0-949.0) | 202.0 (129.5-259.0) | 0.03 |
| Ultra-Processed Foods (N) | 2169 | 2123 | 46 |  |
| Calcium, mg | 52.0 (16.0-126.0) | 52.0 (16.0-126.0) | 58.0 (11.0-138.0) | 0.98 |
| Phosphorus, mg | 120.0 (60.0-195.0) | 120.0 (61.0-193.0) | 182.0 (18.0-277.0) | 0.08 |
| Magnesium, mg | 21.0 (12.0-37.0) | 21.0 (12.0-36.0) | 53.5 (11.0-131.0) | <0.001 |
| Iron, mg | 1.4 (0.5-2.5) | 1.4 (0.5-2.5) | 1.5 (0.2-2.7) | 0.96 |
| Zinc, mg | 0.9 (0.4-1.5) | 0.8 (0.4-1.5) | 1.3 (0.1-2.6) | 0.39 |
| Copper, mg | 0.1 (0.0-0.2) | 0.1 (0.0-0.2) | 0.1 (0.0-0.3) | 0.07 |
| Selenium, mcg | 8.6 (2.0-18.9) | 8.5 (2.1-18.7) | 15.2 (0.4-25.8) | 0.15 |
| Potassium, mg | 179.0 (110.0-251.0) | 177.0 (109.0-250.0) | 251.5 (131.0-381.0) | 0.42 |
| Sodium, mg | 391.0 (180.0-576.0) | 394.0 (188.0-583.0) | 162.5 (14.0-381.0) | <0.001 |

1. Abbreviations: milligrams (mg), microgram (mcg), interquartile range (IQR)
2. P-values from t-tests comparing mean log-transformed nutrient values between FDA-aligned and FDA-unaligned groups

**Supplemental Table 8.** Mean Minerals per 100g of FDA-Aligned and FDA-Unaligned Foods and Beverages Consumed in the United States NHANES/FNDDS 2017-2018, Overall and Stratified by Food Category^1^

|  | **Overall** | **FDA Unaligned** | **FDA Aligned** | **p-value^2^** |
| --- | --- | --- | --- | --- |
| Beverages (N) | 422 | 324 | 98 |  |
| Calcium, mg | 8.0 (2.0-60.0) | 7.0 (3.0-62.5) | 11.0 (2.0-42.0) | 0.62 |
| Phosphorus, mg | 9.0 (2.0-39.0) | 9.0 (2.0-49.0) | 11.5 (1.0-27.0) | 0.96 |
| Magnesium, mg | 5.0 (1.0-14.0) | 4.0 (1.0-15.0) | 7.5 (3.0-14.0) | 0.62 |
| Iron, mg | 0.1 (0.0-0.3) | 0.1 (0.0-0.3) | 0.1 (0.0-0.3) | 0.50 |
| Zinc, mg | 0.1 (0.0-0.2) | 0.1 (0.0-0.2) | 0.1 (0.0-0.2) | 0.67 |
| Copper, mg | 0.0 (0.0-0.0) | 0.0 (0.0-0.0) | 0.0 (0.0-0.0) | 0.88 |
| Selenium, mcg | 0.1 (0.0-0.9) | 0.2 (0.0-1.0) | 0.1 (0.0-0.4) | 0.21 |
| Potassium, mg | 49.0 (11.0-138.0) | 33.5 (8.0-116.5) | 105.0 (37.0-180.0) | <0.001 |
| Sodium, mg | 10.0 (4.0-39.0) | 14.0 (5.0-47.0) | 4.5 (2.0-19.0) | <0.001 |
| Grains (N) | 437 | 416 | 21 |  |
| Calcium, mg | 72.0 (23.0-162.0) | 72.5 (23.5-162.5) | 69.0 (12.0-138.0) | 0.80 |
| Phosphorus, mg | 125.0 (80.0-191.0) | 125.0 (80.0-185.0) | 163.0 (65.0-228.0) | 0.87 |
| Magnesium, mg | 30.0 (21.0-54.0) | 30.0 (21.0-53.0) | 37.0 (24.0-78.0) | 0.18 |
| Iron, mg | 3.0 (2.0-7.1) | 3.0 (2.0-7.5) | 2.6 (0.7-4.3) | 0.05 |
| Zinc, mg | 1.0 (0.6-1.9) | 1.0 (0.6-1.8) | 1.1 (0.6-2.6) | 0.73 |
| Copper, mg | 0.1 (0.1-0.2) | 0.1 (0.1-0.2) | 0.1 (0.1-0.3) | 0.41 |
| Selenium, mcg | 14.0 (5.6-26.1) | 14.1 (5.7-26.1) | 9.0 (4.8-29.2) | 0.49 |
| Potassium, mg | 148.0 (98.0-214.0) | 146.0 (98.5-211.5) | 196.0 (58.0-281.0) | 0.74 |
| Sodium, mg | 424.0 (213.0-534.0) | 439.0 (230.5-544.0) | 73.0 (70.0-353.0) | <0.001 |
| Vegetables (N) | 525 | 212 | 313 |  |
| Calcium, mg | 27.0 (16.0-45.0) | 25.0 (13.5-54.5) | 28.0 (18.0-44.0) | 0.35 |
| Phosphorus, mg | 49.0 (33.0-67.0) | 56.0 (47.0-79.5) | 42.0 (29.0-58.0) | <0.001 |
| Magnesium, mg | 21.0 (13.0-27.0) | 22.0 (16.0-26.0) | 20.0 (13.0-28.0) | 0.60 |
| Iron, mg | 0.6 (0.4-1.0) | 0.6 (0.3-1.0) | 0.6 (0.5-1.1) | 0.02 |
| Zinc, mg | 0.3 (0.2-0.5) | 0.3 (0.3-0.5) | 0.3 (0.2-0.5) | 0.01 |
| Copper, mg | 0.1 (0.1-0.1) | 0.1 (0.1-0.2) | 0.1 (0.0-0.1) | <0.001 |
| Selenium, mcg | 0.7 (0.4-1.4) | 0.8 (0.4-2.8) | 0.6 (0.4-1.0) | <0.001 |
| Potassium, mg | 260.0 (177.0-363.0) | 308.5 (212.5-405.0) | 242.0 (168.0-318.0) | <0.001 |
| Sodium, mg | 165.0 (131.0-229.0) | 246.0 (178.0-336.0) | 139.0 (113.0-176.0) | <0.001 |
| Fruits (N) | 110 | 43 | 67 |  |
| Calcium, mg | 13.0 (7.0-25.0) | 11.0 (5.0-18.0) | 15.0 (9.0-33.0) | 0.02 |
| Phosphorus, mg | 17.0 (11.0-36.0) | 12.0 (8.0-36.0) | 21.0 (14.0-37.0) | <0.01 |
| Magnesium, mg | 11.0 (8.0-22.0) | 8.0 (5.0-20.0) | 12.0 (10.0-23.0) | 0.01 |
| Iron, mg | 0.3 (0.2-0.5) | 0.3 (0.2-0.4) | 0.4 (0.2-0.7) | 0.16 |
| Zinc, mg | 0.1 (0.1-0.3) | 0.1 (0.1-0.3) | 0.1 (0.1-0.3) | 0.36 |
| Copper, mg | 0.1 (0.1-0.2) | 0.1 (0.0-0.1) | 0.1 (0.1-0.2) | 0.04 |
| Selenium, mcg | 0.5 (0.2-0.7) | 0.4 (0.3-0.9) | 0.5 (0.1-0.7) | 0.33 |
| Potassium, mg | 155.0 (116.0-222.0) | 123.0 (87.0-184.0) | 168.0 (135.0-290.0) | <0.001 |
| Sodium, mg | 3.0 (1.0-7.0) | 4.0 (3.0-7.0) | 2.0 (1.0-9.0) | 0.04 |
| Legumes (N) | 80 | 45 | 35 |  |
| Calcium, mg | 42.5 (26.5-62.0) | 50.0 (33.0-75.0) | 39.0 (25.0-49.0) | 0.05 |
| Phosphorus, mg | 144.5 (110.5-167.0) | 148.0 (106.0-170.0) | 139.0 (116.0-156.0) | 0.83 |
| Magnesium, mg | 46.0 (36.0-56.5) | 43.0 (32.0-55.0) | 48.0 (42.0-59.0) | 0.32 |
| Iron, mg | 2.2 (1.9-2.7) | 2.1 (1.6-2.5) | 2.3 (2.0-3.1) | 0.14 |
| Zinc, mg | 1.0 (0.9-1.3) | 1.0 (0.8-1.3) | 1.0 (0.9-1.3) | 0.11 |
| Copper, mg | 0.2 (0.2-0.3) | 0.2 (0.2-0.3) | 0.2 (0.2-0.3) | 0.16 |
| Selenium, mcg | 4.0 (1.8-6.0) | 5.6 (2.6-7.4) | 2.4 (1.3-4.6) | 0.02 |
| Potassium, mg | 367.5 (279.5-436.0) | 355.0 (231.0-436.0) | 375.0 (319.0-436.0) | 0.05 |
| Sodium, mg | 254.0 (218.0-349.0) | 335.0 (299.0-391.0) | 218.0 (217.0-227.0) | <0.01 |
| Nuts, Seeds (N) | 77 | 24 | 53 |  |
| Calcium, mg | 70.0 (52.0-104.0) | 53.5 (39.0-69.0) | 88.0 (60.0-134.0) | <0.01 |
| Phosphorus, mg | 391.0 (319.0-481.0) | 321.5 (221.0-368.5) | 453.0 (363.0-508.0) | <0.01 |
| Magnesium, mg | 176.0 (121.0-251.0) | 140.5 (104.0-172.5) | 197.0 (129.0-260.0) | 0.01 |
| Iron, mg | 3.2 (2.2-4.2) | 2.2 (1.7-3.6) | 3.6 (2.7-4.7) | 0.01 |
| Zinc, mg | 3.2 (2.5-4.4) | 2.5 (2.0-3.5) | 3.3 (3.1-4.5) | 0.02 |
| Copper, mg | 1.1 (0.6-1.5) | 0.7 (0.4-1.2) | 1.2 (1.0-1.6) | <0.001 |
| Selenium, mcg | 9.3 (3.8-34.4) | 7.5 (3.8-13.1) | 9.7 (3.8-36.0) | 0.20 |
| Potassium, mg | 615.0 (480.0-726.0) | 480.5 (358.5-668.0) | 634.0 (565.0-748.0) | <0.001 |
| Sodium, mg | 160.0 (7.0-383.0) | 385.0 (257.5-453.0) | 18.0 (6.0-252.0) | <0.001 |
| Meat (N) | 472 | 458 | 14 |  |
| Calcium, mg | 14.5 (10.0-24.0) | 14.0 (10.0-22.0) | 45.0 (7.0-56.0) | 0.08 |
| Phosphorus, mg | 212.0 (183.0-242.0) | 212.0 (183.0-240.0) | 203.0 (195.0-260.0) | 0.01 |
| Magnesium, mg | 22.0 (19.0-25.0) | 22.0 (19.0-25.0) | 12.0 (11.0-28.0) | <0.01 |
| Iron, mg | 1.1 (0.8-1.8) | 1.1 (0.8-1.7) | 2.2 (1.7-3.9) | 0.06 |
| Zinc, mg | 2.0 (1.5-3.1) | 2.0 (1.5-3.1) | 1.4 (1.3-3.7) | 0.03 |
| Copper, mg | 0.1 (0.1-0.1) | 0.1 (0.1-0.1) | 0.1 (0.1-0.2) | 0.94 |
| Selenium, mcg | 26.5 (22.3-31.6) | 26.5 (22.3-31.6) | 30.5 (17.6-30.7) | 0.93 |
| Potassium, mg | 279.0 (237.0-352.0) | 280.0 (240.0-359.0) | 150.5 (136.0-311.0) | <0.001 |
| Sodium, mg | 429.0 (381.0-697.0) | 440.0 (384.0-705.0) | 255.5 (166.0-267.0) | <0.0001 |
| Seafood (N) | 193 | 176 | 17 |  |
| Calcium, mg | 40.0 (16.0-70.0) | 40.0 (14.0-70.5) | 47.0 (32.0-66.0) | 0.91 |
| Phosphorus, mg | 255.0 (220.0-292.0) | 256.5 (221.0-295.5) | 235.0 (215.0-261.0) | 0.01 |
| Magnesium, mg | 30.0 (25.0-36.0) | 31.0 (26.0-37.0) | 24.0 (23.0-33.0) | 0.01 |
| Iron, mg | 1.0 (0.5-1.3) | 1.0 (0.5-1.3) | 0.9 (0.7-1.9) | 0.35 |
| Zinc, mg | 0.7 (0.5-1.2) | 0.7 (0.5-1.2) | 1.1 (0.6-1.4) | 0.49 |
| Copper, mg | 0.1 (0.1-0.2) | 0.1 (0.1-0.2) | 0.2 (0.1-0.3) | 0.05 |
| Selenium, mcg | 36.3 (28.4-45.9) | 36.3 (28.4-46.0) | 36.5 (30.6-42.6) | 0.92 |
| Potassium, mg | 312.0 (241.0-382.0) | 321.0 (249.0-391.0) | 176.0 (101.0-294.0) | <0.001 |
| Sodium, mg | 405.0 (379.0-447.0) | 408.0 (384.5-451.5) | 347.0 (85.0-372.0) | <0.001 |
| Dairy (N) | 196 | 174 | 22 |  |
| Calcium, mg | 132.0 (105.0-528.5) | 131.5 (102.0-557.0) | 132.0 (115.0-183.0) | 0.64 |
| Phosphorus, mg | 129.0 (98.0-361.0) | 129.0 (97.0-387.0) | 136.5 (103.0-144.0) | 0.43 |
| Magnesium, mg | 15.0 (11.0-26.0) | 15.5 (10.0-27.0) | 12.0 (11.0-17.0) | 0.78 |
| Iron, mg | 0.1 (0.0-0.3) | 0.2 (0.0-0.4) | 0.0 (0.0-0.1) | <0.001 |
| Zinc, mg | 0.6 (0.4-2.2) | 0.6 (0.4-2.4) | 0.6 (0.4-0.9) | 0.95 |
| Copper, mg | 0.0 (0.0-0.0) | 0.0 (0.0-0.0) | 0.0 (0.0-0.0) | <0.001 |
| Selenium, mcg | 3.3 (2.0-14.5) | 3.2 (1.9-14.5) | 3.3 (2.1-9.9) | 0.47 |
| Potassium, mg | 157.5 (125.0-209.0) | 155.0 (121.0-203.0) | 162.5 (159.0-234.0) | 0.13 |
| Sodium, mg | 72.0 (45.0-532.5) | 79.0 (49.0-600.0) | 51.0 (39.0-70.0) | <0.01 |
| Fats and Oils (N) | 68 | 60 | 8 |  |
| Calcium, mg | 15.0 (6.0-26.5) | 20.0 (7.5-27.5) | 0.0 (0.0-0.0) | - |
| Phosphorus, mg | 18.0 (9.5-29.0) | 20.0 (13.0-32.5) | 0.0 (0.0-0.0) | - |
| Magnesium, mg | 2.0 (1.0-5.0) | 2.0 (2.0-5.0) | 0.0 (0.0-0.0) | - |
| Iron, mg | 0.2 (0.0-0.3) | 0.2 (0.1-0.3) | 0.0 (0.0-0.1) | 0.54 |
| Zinc, mg | 0.1 (0.0-0.2) | 0.1 (0.1-0.2) | 0.0 (0.0-0.0) | - |
| Copper, mg | 0.0 (0.0-0.0) | 0.0 (0.0-0.0) | 0.0 (0.0-0.0) | - |
| Selenium, mcg | 1.4 (0.0-1.9) | 1.6 (0.2-2.0) | 0.0 (0.0-0.0) | - |
| Potassium, mg | 36.0 (23.5-70.0) | 41.0 (29.5-77.5) | 0.0 (0.0-0.0) | - |
| Sodium, mg | 674.0 (583.0-897.0) | 750.5 (625.0-901.0) | 0.0 (0.0-0.0) | - |
| Mixed Dishes (N) | 1510 | 1441 | 69 |  |
| Calcium, mg | 51.5 (18.0-124.0) | 53.0 (19.0-129.0) | 18.0 (9.0-46.0) | <0.0001 |
| Phosphorus, mg | 118.0 (72.0-184.0) | 122.0 (72.0-186.0) | 93.0 (76.0-119.0) | <0.001 |
| Magnesium, mg | 19.0 (14.0-24.0) | 18.0 (14.0-23.0) | 32.0 (21.0-36.0) | <0.0001 |
| Iron, mg | 1.3 (0.8-1.8) | 1.3 (0.8-1.9) | 0.6 (0.5-1.3) | <0.0001 |
| Zinc, mg | 0.9 (0.6-1.5) | 1.0 (0.6-1.5) | 0.7 (0.5-0.9) | <0.0001 |
| Copper, mg | 0.1 (0.1-0.1) | 0.1 (0.1-0.1) | 0.1 (0.1-0.1) | 0.01 |
| Selenium, mcg | 14.1 (7.2-20.6) | 14.3 (7.5-20.8) | 5.8 (4.6-18.7) | <0.0001 |
| Potassium, mg | 182.0 (131.0-232.0) | 182.0 (131.0-232.0) | 196.0 (126.0-241.0) | 0.11 |
| Sodium, mg | 369.0 (290.0-493.0) | 378.0 (302.0-503.0) | 197.0 (164.0-257.0) | <0.0001 |
| Sauces and Condiments (N) | 178 | 169 | 9 |  |
| Calcium, mg | 25.5 (10.0-60.0) | 26.0 (10.0-60.0) | 13.0 (10.0-14.0) | 0.39 |
| Phosphorus, mg | 30.0 (9.0-60.0) | 30.0 (10.0-60.0) | 14.0 (9.0-34.0) | 0.33 |
| Magnesium, mg | 9.5 (4.0-19.0) | 10.0 (4.0-19.0) | 8.0 (7.0-19.0) | 0.91 |
| Iron, mg | 0.4 (0.1-0.9) | 0.4 (0.2-0.9) | 0.1 (0.1-0.6) | 0.13 |
| Zinc, mg | 0.2 (0.1-0.5) | 0.2 (0.1-0.5) | 0.1 (0.1-0.2) | 0.42 |
| Copper, mg | 0.0 (0.0-0.1) | 0.0 (0.0-0.1) | 0.0 (0.0-0.1) | 0.71 |
| Selenium, mcg | 0.9 (0.5-3.0) | 0.9 (0.5-3.0) | 0.1 (0.1-0.4) | <0.01 |
| Potassium, mg | 113.5 (54.0-225.0) | 113.0 (52.0-225.0) | 117.0 (103.0-265.0) | 0.27 |
| Sodium, mg | 476.0 (80.0-808.0) | 498.0 (150.0-835.0) | 6.0 (2.0-17.0) | <0.001 |
| Snacks and Desserts (N) | 680 | 671 | 9 |  |
| Calcium, mg | 48.0 (21.0-104.5) | 48.0 (21.0-104.0) | 31.0 (11.0-238.0) | 0.60 |
| Phosphorus, mg | 114.0 (77.0-187.0) | 113.0 (77.0-184.0) | 360.0 (277.0-374.0) | <0.001 |
| Magnesium, mg | 26.0 (14.0-58.0) | 26.0 (14.0-55.0) | 143.0 (131.0-164.0) | <0.001 |
| Iron, mg | 1.6 (0.9-2.7) | 1.6 (0.9-2.7) | 2.4 (1.6-2.8) | 0.19 |
| Zinc, mg | 0.7 (0.4-1.2) | 0.7 (0.4-1.2) | 2.6 (2.6-3.0) | <0.001 |
| Copper, mg | 0.1 (0.1-0.2) | 0.1 (0.1-0.2) | 0.4 (0.3-0.5) | <0.001 |
| Selenium, mcg | 6.5 (2.9-11.0) | 6.3 (2.9-10.8) | 17.4 (9.9-24.6) | 0.01 |
| Potassium, mg | 171.0 (110.0-253.0) | 170.0 (108.0-250.0) | 381.0 (327.0-381.0) | <0.01 |
| Sodium, mg | 275.0 (148.5-445.0) | 273.0 (145.0-448.0) | 329.0 (288.0-438.0) | 0.52 |

1. Abbreviations: milligrams (mg), microgram (mcg), interquartile range (IQR)
2. P-values from t-tests comparing mean log-transformed nutrient values between FDA-aligned and FDA-unaligned groups

**Supplemental Table 9.** Mean Macronutrients per 100kcal of FDA-Aligned and FDA-Unaligned Foods and Beverages Consumed in the United States NHANES/FNDDS 2017-2018, Overall and Stratified by Nova^1^

|  | **Overall**  **Median (IQR)** | **FDA Unaligned**  **Median (IQR)** | **FDA Aligned**  **Median (IQR)** | **p-value^2^** |
| --- | --- | --- | --- | --- |
| Overall (N) |  |  |  |  |
| Protein, g | 3.7 (1.7, 6.4) | 3.7 (1.7, 6.5) | 3.5 (2.0, 6.0) | 0.14 |
| Carbohydrate, g | 11.6 (5.8, 17.1) | 11.0 (5.4, 16.3) | 15.0 (9.6, 21.6) | <0.001 |
| Sugars, added, g | 0.0 (0.0, 2.2) | 0.2 (0.0, 3.4) | 0.0 (0.0, 0.0) | <0.001 |
| Fiber, total dietary, g | 0.6 (0.1, 1.4) | 0.5 (0.1, 1.1) | 2.8 (1.3, 5.1) | <0.001 |
| Saturated fat, g | 1.0 (0.4, 1.7) | 1.1 (0.5, 1.9) | 0.4 (0.1, 1.1) | <0.001 |
| Monounsaturated fat, g | 1.3 (0.5, 2.0) | 1.3 (0.6, 2.0) | 0.7 (0.1, 1.7) | <0.001 |
| Polyunsaturated fat, g | 0.7 (0.3, 1.4) | 0.7 (0.3, 1.3) | 0.7 (0.2, 1.6) | 0.08 |
| Minimally Processed Foods (N) |  |  |  |  |
| Protein, g | 4.9 (2.6, 8.7) | 5.6 (2.9, 9.4) | 3.7 (2.0, 6.3) | <0.001 |
| Carbohydrate, g | 10.3 (3.6, 16.2) | 8.5 (1.2, 13.4) | 15.6 (10.3, 21.8) | <0.001 |
| Sugars, added, g | 0.0 (0.0, 0.0) | 0.0 (0.0, 0.3) | 0.0 (0.0, 0.0) | 0.06 |
| Fiber, total dietary, g | 0.7 (0.1, 1.9) | 0.4 (0.0, 1.0) | 3.3 (1.4, 5.4) | <0.001 |
| Saturated fat, g | 1.0 (0.5, 1.6) | 1.2 (0.7, 1.8) | 0.4 (0.1, 1.1) | <0.001 |
| Monounsaturated fat, g | 1.4 (0.6, 2.1) | 1.5 (0.9, 2.2) | 0.5 (0.1, 1.7) | <0.001 |
| Polyunsaturated fat, g | 0.8 (0.3, 1.5) | 0.8 (0.4, 1.5) | 0.6 (0.2, 1.6) | <0.001 |
| Processed Culinary Ingredients (N) |  |  |  |  |
| Protein, g | 0.1 (0.0, 1.6) | 0.3 (0.1, 1.7) | 0.0 (0.0, 0.0) | - |
| Carbohydrate, g | 6.8 (0.0, 23.5) | 9.5 (1.9, 24.6) | 0.0 (0.0, 0.0) | - |
| Sugars, added, g | 0.0 (0.0, 14.3) | 0.0 (0.0, 22.4) | 0.0 (0.0, 0.0) | - |
| Fiber, total dietary, g | 0.0 (0.0, 0.0) | 0.0 (0.0,0.1) | 0.0 (0.0, 0.0) | - |
| Saturated fat, g | 1.6 (0.0, 5.4) | 3.4 (0.0, 5.7) | 1.2 (0.9, 1.5) | 0.32 |
| Monounsaturated fat, g | 1.7 (0.0, 2.9) | 1.3 (0.0, 2.5) | 4.5 (2.6, 7.2) | 0.13 |
| Polyunsaturated fat, g | 0.2 (0.0, 0.5) | 0.2 (0.0, 0.4) | 4.6 (2.0, 6.1) | <0.01 |
| Processed Foods (N) |  |  |  |  |
| Protein, g | 4.7 (1.7, 7.3) | 5.4 (1.6, 8.0) | 3.3 (2.5, 4.6) | 0.52 |
| Carbohydrate, g | 5.4 (1.2, 15.3) | 5.7 (1.0, 14.8) | 5.1 (3.1, 15.8) | 0.31 |
| Sugars, added, g | 0.0 (0.0, 0.6) | 0.0 (0.0, 0.9) | 0.0 (0.0, 0.0) | <0.001 |
| Fiber, total dietary, g | 0.6 (0.0, 2.2) | 0.3 (0.0, 1.9) | 1.7 (1.3, 3.3) | 0.01 |
| Saturated fat, g | 1.1 (0.2, 2.0) | 1.2 (0.1, 2.4) | 1.1 (0.5, 1.4) | 0.93 |
| Monounsaturated fat, g | 1.5 (0.1, 2.3) | 1.4 (0.1, 2.2) | 2.2 (0.7, 4.7) | 0.01 |
| Polyunsaturated fat, g | 0.5 (0.2, 1.4) | 0.4 (0.1, 1.2) | 1.5 (0.6, 2.3) | <0.001 |
| Ultra-Processed Foods (N) |  |  |  |  |
| Protein, g | 2.6 (1.2, 4.5) | 2.6 (1.2, 4.4) | 3.2 (1.7, 4.8) | 0.03 |
| Carbohydrate, g | 13.3 (9.1, 18.2) | 13.2 (9.1, 18.0) | 17.6 (14.6, 22.0) | 0.02 |
| Sugars, added, g | 1.1 (0.0, 6.6) | 1.2 (0.0, 6.7) | 0.0 (0.0, 1.1) | 0.04 |
| Fiber, total dietary, g | 0.6 (0.2, 1.1) | 0.6 (0.2, 1.1) | 1.7 (1.1, 2.4) | <0.001 |
| Saturated fat, g | 1.0 (0.3, 1.7) | 1.0 (0.3, 1.8) | 0.2 (0.1, 0.4) | <0.001 |
| Monounsaturated fat, g | 1.1 (0.4, 1.9) | 1.1 (0.4, 1.9) | 0.3 (0.1, 0.8) | <0.001 |
| Polyunsaturated fat, g | 0.6 (0.2, 1.2) | 0.6 (0.2, 1.2) | 0.4 (0.2, 0.9) | 0.31 |

1. Abbreviations: grams (g), interquartile range (IQR)
2. P-values from t-tests comparing mean log-transformed nutrient values between FDA-aligned and FDA-unaligned groups

**Supplemental Table 10.** Mean Macronutrients per 100kcal of FDA-Aligned and FDA-Unaligned Foods and Beverages Consumed in the United States NHANES/FNDDS 2017-2018, Overall and Stratified by Food Category ^1^

|  | **Overall**  **Median (IQR)** | **FDA Unaligned**  **Median (IQR)** | **FDA Aligned**  **Median (IQR)** | **p-value^2^** |
| --- | --- | --- | --- | --- |
| Beverages (N) | 422 | 324 | 98 |  |
| Protein, g | 0.7 (0.0, 3.1) | 0.6 (0.0, 2.9) | 1.4 (0.6, 4.9) | <0.01 |
| Carbohydrate, g | 19.1 (11.8, 24.6) | 17.7 (10.4, 24.7) | 23.2 (17.0, 24.6) | <0.01 |
| Sugars, added, g | 2.3 (0.0, 13.6) | 7.7 (0.0, 16.5) | 0.0 (0.0, 0.0) | <0.001 |
| Fiber, total dietary, g | 0.0 (0.0, 0.5) | 0.0 (0.0, 0.2) | 0.4 (0.0, 1.4) | <0.001 |
| Saturated fat, g | 0.0 (0.0, 0.3) | 0.0 (0.0, 0.3) | 0.1 (0.0, 0.2) | 0.15 |
| Monounsaturated fat, g | 0.0 (0.0, 0.3) | 0.0 (0.0, 0.4) | 0.0 (0.0, 0.1) | 0.08 |
| Polyunsaturated fat, g | 0.0 (0.0, 0.1) | 0.0 (0.0, 0.1) | 0.1 (0.1, 0.2) | 0.18 |
| Grains (N) | 437 | 416 | 21 |  |
| Protein, g | 2.6 (1.8, 3.5) | 2.5 (1.8, 3.5) | 3.5 (3.3, 4.6) | 0.09 |
| Carbohydrate, g | 19.0 (16.8, 21.5) | 19.0 (16.8, 21.5) | 18.5 (17.6, 21.8) | 0.10 |
| Sugars, added, g | 1.7 (0.0, 5.2) | 1.8 (0.1, 5.4) | 0.0 (0.0, 1.7) | 0.31 |
| Fiber, total dietary, g | 1.4 (0.7, 2.2) | 1.3 (0.7, 2.2) | 2.7 (2.2, 2.8) | <0.001 |
| Saturated fat, g | 0.3 (0.1, 0.8) | 0.3 (0.1, 0.8) | 0.3 (0.1, 0.3) | 0.06 |
| Monounsaturated fat, g | 0.4 (0.2, 0.8) | 0.4 (0.2, 0.8) | 0.3 (0.2, 0.5) | 0.22 |
| Polyunsaturated fat, g | 0.4 (0.3, 0.7) | 0.4 (0.3, 0.7) | 0.6 (0.4, 0.6) | 0.79 |
| Vegetables (N) | 525 | 212 | 313 |  |
| Protein, g | 2.9 (1.8, 5.2) | 1.9 (1.4, 3.0) | 4.1 (2.5, 6.1) | <0.001 |
| Carbohydrate, g | 13.9 (10.3, 18.1) | 12.4 (9.4, 16.1) | 15.0 (11.6, 19.8) | <0.001 |
| Sugars, added, g | 0.0 (0.0, 0.0) | 0.0 (0.0, 0.0) | 0.0 (0.0, 0.0) | 0.13 |
| Fiber, total dietary, g | 3.6 (1.5, 5.8) | 1.4 (0.9, 2.4) | 4.9 (3.3, 7.0) | <0.001 |
| Saturated fat, g | 0.9 (0.4, 1.6) | 1.0 (0.8, 2.0) | 0.7 (0.2, 1.4) | <0.001 |
| Monounsaturated fat, g | 1.3 (0.2. 1.9) | 1.5 (1.1, 2.1) | 1.1 (0.1, 1.8) | <0.001 |
| Polyunsaturated fat, g | 1.1 (0.4, 1.8) | 1.3 (0.6, 2.1) | 1.0 (0.4, 1.7) | 0.06 |
| Fruits (N) | 110 | 43 | 67 |  |
| Protein, g | 1.1 (0.8, 1.6) | 0.8 (0.5, 1.0) | 1.4 (1.0, 2.0) | <0.001 |
| Carbohydrate, g | 25.4 (24.1, 26.1) | 25.4 (23.1, 25.9) | 25.4 (24.1, 26.2) | 0.02 |
| Sugars, added, g | 0.0 (0.0, 2.5) | 7.8 (1.2, 11.6) | 0.0 (0.0, 0.0) | 0.04 |
| Fiber, total dietary, g | 2.6 (1.7, 3.8) | 1.8 (1.3, 2.3) | 3.4 (2.6, 5.2) | <0.001 |
| Saturated fat, g | 0.0 (0.0, 0.1) | 0.0 (0.0, 0.5) | 0.1 (0.0, 0.1) | 0.43 |
| Monounsaturated fat, g | 0.1 (0.0, 0.1) | 0.0 (0.0, 0.2) | 0.1 (0.0, 0.1) | 0.60 |
| Polyunsaturated fat, g | 0.1 (0.1, 0.2) | 0.1 (0.0, 0.1) | 0.1 (0.1, 0.2) | 0.25 |
| Legumes (N) | 80 | 45 | 35 |  |
| Protein, g | 4.8 (4.3, 6.3) | 5.5 (4.3, 6.5) | 4.6 (4.4, 5.4) | 0.22 |
| Carbohydrate, g | 12.4 (11.3, 15.8) | 12.2 (10.6, 15.4) | 12.5 (11.8, 16.7) | 0.05 |
| Sugars, added, g | 0.0 (0.0, 0.0) | 0.0 (0.0, 0.0) | 0.0 (0.0, 0.0) | - |
| Fiber, total dietary, g | 4.0 (3.2, 4.6) | 3.9 (2.8, 4.4) | 4.4 (3.4, 4.6) | 0.04 |
| Saturated fat, g | 0.6 (0.3, 0.7) | 0.6 (0.3, 0.8) | 0.5 (0.2, 0.6) | 0.03 |
| Monounsaturated fat, g | 1.5 (0.4, 1.7) | 1.4 (0.5, 1.7) | 1.5 (0.4, 1.7) | 0.64 |
| Polyunsaturated fat, g | 1.5 (0.4, 1.8) | 1.4 (0.4, 2.0) | 1.5 (0.6, 1.7) | 0.57 |
| Nuts and Seeds (N) | 77 | 24 | 53 |  |
| Protein, g | 3.2 (2.4, 3.7) | 2.7 (2.0, 3.7) | 3.3 (2.5, 3.5) | 0.18 |
| Carbohydrate, g | 3.7 (3.1, 5.4) | 5.2 (3.9, 8.2) | 3.5 (2.6, 4.1) | <0.001 |
| Sugars, added, g | 0.0 (0.0, 0.5) | 1.2 (0.6, 3.8) | 0.0 (0.0, 0.0) | 0.01 |
| Fiber, total dietary, g | 1.3 (1.0, 1.7) | 1.0 (0.8, 1.5) | 1.4 (1.2, 1.7) | 0.06 |
| Saturated fat, g | 1.2 (0.9, 1.5) | 1.2 (0.9, 1.7) | 1.2 (0.9, 1.3) | 0.08 |
| Monounsaturated fat, g | 4.3 (2.9, 4.8) | 3.7 (2.7, 4.2) | 4.5 (3.8, 4.9) | 0.03 |
| Polyunsaturated fat, g | 2.3 (1.7, 3.1) | 2.1 (1.7, 2.3) | 2.4 (2.1, 3.2) | 0.09 |
| Meat (N) | 472 | 458 | 14 |  |
| Protein, g | 10.7 (7.2, 14.2) | 10.7 (7.2, 14.1) | 9.9 (8.3, 19.0) | 0.15 |
| Carbohydrate, g | 0.1 (0.0, 1.8) | 0.1 (0.0, 1.9) | 0.5 (0.0, 1.1) | 0.77 |
| Sugars, added, g | 0.0 (0.0, 0.0) | 0.0 (0.0, 0.0) | 0.0 (0.0, 0.0) | - |
| Fiber, total dietary, g | 0.0 (0.0, 0.0,) | 0.0 (0.0, 0.0) | 0.0 (0.0, 0.0) | - |
| Saturated fat, g | 1.5 (1.1, 2.1) | 1.5 (1.1, 2.2) | 1.6 (0.9, 2.2) | 0.66 |
| Monounsaturated fat, g | 2.3 (1.6, 2.9) | 2.3 (1.7, 2.9) | 1.9 (0.5, 2.6) | 0.01 |
| Polyunsaturated fat, g | 0.9 (0.5, 1.3) | 0.9 (0.5, 1.3) | 0.9 (0.1, 1.3) | 0.20 |
| Seafood (N) | 193 | 176 | 17 |  |
| Protein, g | 13.0 (8.8, 16.4) | 12.7 (8.8, 16.1) | 16.1 (11.2, 17.1) | 0.12 |
| Carbohydrate, g | 0.9 (0.1, 5.0) | 0.1 (0.1, 5.2) | 2.6 (0.1, 4.2) | 0.21 |
| Sugars, added, g | 0.0 (0.0, 0.2) | 0.0 (0.0, 0.3) | 0.0 (0.0, 0.0) | 0.99 |
| Fiber, total dietary, g | 0.0 (0.0, 0.3) | 0.0 (0.0, 0.3) | 0.0 (0.0, 0.0) | 0.06 |
| Saturated fat, g | 0.8 (0.5, 1.0) | 0.8 (0.6, 1.1) | 0.5 (0.4, 0.7) | 0.01 |
| Monounsaturated fat, g | 1.4 (0.6, 1.9) | 1.4 (0.7, 1.9) | 1.1 (0.3, 1.2) | 0.06 |
| Polyunsaturated fat, g | 1.1 (0.5, 1.6) | 1.2 (0.5, 1.7) | 0.8 (0.5, 1.3) | 0.18 |
| Dairy (N) | 196 | 174 | 22 |  |
| Protein, g | 5.7 (4.1, 7.9) | 5.4 (3.9, 7.0) | 9.9 (8.3, 10.2) | <0.001 |
| Carbohydrate, g | 10.0 (3.0, 14.4) | 8.9 (2.9, 14.2) | 12.1 (11.2, 14.4) | 0.02 |
| Sugars, added, g | 0.0 (0.0, 6.2) | 0.0 (0.0, 6.9) | 0.0 (0.0, 0.0) | - |
| Fiber, total dietary, g | 0.0 (0.0, 0.0) | 0.0 (0.0, 0.0) | 0.0 (0.0, 0.0) | - |
| Saturated fat, g | 1.7 (1.0, 4.1) | 2.2 (1.3, 4.3) | 1.2 (0.2, 1.6) | <0.001 |
| Monounsaturated fat, g | 0.8 (0.5, 1.9) | 1.0 (0.5, 2.0) | 0.5 (0.1, 0.7) | <0.001 |
| Polyunsaturated fat, g | 0.1 (0.1, 0.2) | 0.1 (0.1, 0.2) | 0.1 (0.0, 0.1) | <0.001 |
| Fats and Oils (N) | 68 | 60 | 8 |  |
| Protein, g | 0.2 (0.1, 0.4) | 0.3 (0.1, 0.4) | 0.0 (0.0, 0.0) | - |
| Carbohydrate, g | 1.4 (0.0, 6.6) | 3.4 (0.1, 9.4) | 0.0 (0.0, 0.0) | - |
| Sugars, added, g | 0.8 (0.0, 4.0) | 1.6 (0.0, 4.4) | 0.0 (0.0, 0.0) | - |
| Fiber, total dietary, g | 0.0 (0.0, 0.1) | 0.0 (0.0, 0.1) | 0.0 (0.0, 0.0) | - |
| Saturated fat, g | 1.5 (1.0, 2.4) | 1.5 (1.0, 2.6) | 1.3 (1.0, 1.6) | 0.44 |
| Monounsaturated fat, g | 2.5 (1.9, 3.3) | 2.2 (1.8, 3.2) | 4.6 (2.8, 7.5) | 0.01 |
| Polyunsaturated fat, g | 3.9 (1.2, 5.0) | 3.5 (0.5, 5.0) | 4.7 (2.6, 6.6) | 0.13 |
| Mixed Dishes (N) | 1510 | 1441 | 69 |  |
| Protein, g | 4.7 (3.7, 6.6) | 4.7 (3.7, 6.5) | 4.7 (2.3, 7.5) | 0.21 |
| Carbohydrate, g | 9.6 (6.7, 12.5) | 9.5 (6.8, 12.2) | 14.0 (5.5, 18.0) | 0.06 |
| Sugars, added, g | 0.0 (0.0, 0.7) | 0.0 (0.0, 0.8) | 0.0 (0.0, 0.0) | 0.70 |
| Fiber, total dietary, g | 0.8 (0.4, 1.2) | 0.7 (0.4, 1.1) | 1.6 (1.3, 2.2) | <0.0001 |
| Saturated fat, g | 1.4 (0.8, 2.0) | 1.4 (0.8, 2.0) | 0.5 (0.4, 0.9) | <0.0001 |
| Monounsaturated fat, g | 1.5 (1.1, 2.1) | 1.5 (1.1, 2.1) | 0.9 (0.6, 2.0) | <0.0001 |
| Polyunsaturated fat, g | 0.9 (0.5, 1.4) | 0.8 (0.5, 1.4) | 0.9 (0.5, 1.6) | 0.17 |
| Sauce and Condiments (N) | 178 | 169 | 9 |  |
| Protein, g | 1.5 (0.3, 3.5) | 1.3 (0.3, 3.6) | 1.7 (1.6, 2.6) | 0.33 |
| Carbohydrate, g | 17.2 (7.2, 24.6) | 16.7 (7.0, 24.5) | 31.4 (22.5, 33.1) | 0.02 |
| Sugars, added, g | 0.0 (0.0, 12.9) | 1.5 (0.0, 14.1) | 0.0 (0.0, 0.0) | - |
| Fiber, total dietary, g | 0.7 (0.1, 2.3) | 0.6 (0.1, 2.3) | 1.6 (1.4, 2.5) | 0.08 |
| Saturated fat, g | 0.3 (0.0, 1.6) | 0.3 (0.0, 1.7) | 0.2 (0.1, 0.2) | 0.12 |
| Monounsaturated fat, g | 0.2 (0.0, 2.0) | 0.2 (0.0, 2.0) | 0.0 (0.0, 0.1) | 0.01 |
| Polyunsaturated fat, g | 0.3 (0.0, 1.1) | 0.3 (0.0, 1.1) | 0.1 (0.1, 0.3) | 0.23 |
| Snack and Desserts (N) | 680 | 671 | 9 |  |
| Protein, g | 1.4 (1.1, 2.0) | 1.4 (1.1, 2.0) | 2.5 (2.1, 2.5) | 0.03 |
| Carbohydrate, g | 14.5 (12.0, 17.4) | 14.5 (11.9, 17.2) | 20.1 (14.6, 20.9) | 0.04 |
| Sugars, added, g | 5.7 (0.9, 8.9) | 5.8 (1.3, 8.9) | 0.0 (0.0, 0.0) | - |
| Fiber, total dietary, g | 0.6 (0.3, 0.9) | 0.6 (0.3, 0.8) | 2.3 (1.1, 2.3) | <0.001 |
| Saturated fat, g | 1.0 (0.6, 1.9) | 1.1 (0.6, 2.0) | 0.2 (0.2, 0.5) | <0.001 |
| Monounsaturated fat, g | 1.3 (0.7, 1.9) | 1.3 (0.7, 1.9) | 0.4 (0.2, 1.5) | 0.03 |
| Polyunsaturated fat, g | 0.8 (0.3, 1.4) | 0.8 (0.3, 1.4) | 0.6 (0.4, 1.3) | 0.96 |

1. Abbreviations: grams (g), interquartile range (IQR)
2. P-values from t-tests comparing mean log-transformed nutrient values between FDA-aligned and FDA-unaligned groups

**Supplemental Table 11.** Mean Vitamins per 100kcal of FDA-Aligned and FDA-Unaligned Foods and Beverages Consumed in the United States NHANES/FNDDS 2017-2018, Overall and Stratified by Nova^1^

|  | **Overall**  **Median (IQR)** | **FDA Unaligned**  **Median (IQR)** | **FDA Aligned**  **Median (IQR)** | **p-value^2^** |
| --- | --- | --- | --- | --- |
| Overall (N) | 4949 | 4214 | 735 |  |
| Vitamin A, mcg_RAE, | 11.0 (0.7, 46.5) | 10.0 (0.7, 40.2) | 22.4 (0.4, 118.2) | <0.001 |
| Thiamin, mg | 0.1 (0.0, 0.1) | 0.1 (0.0, 0.1) | 0.1 (0.0, 0.2) | <0.001 |
| Riboflavin, mg | 0.1 (0.0, 0.1) | 0.1 (0.0, 0.1) | 0.1 (0.0, 0.2) | <0.001 |
| Niacin, mg | 1.0 (0.4, 1.8) | 1.0 (0.4, 1.8) | 1.0 (0.4, 1.7) | 0.01 |
| Vitamin B-6, mg | 0.1 (0.0, 0.2) | 0.1 (0.0, 0.1) | 0.1 (0.1, 0.3) | <0.001 |
| Folate, mcg_DFE | 15.0 (4.8, 34.0) | 13.3 (4.1, 29.4) | 30.2 (12.0, 75.5) | <0.001 |
| Vitamin B-12, mcg | 0.1 (0.0, 0.3) | 0.1 (0.0, 0.4) | 0.0 (0.0, 0.0) | <0.001 |
| Vitamin C, mg | 0.3 (0.0, 3.7) | 0.2 (0.0, 2.0) | 8.6 (0.4, 38.3) | <0.001 |
| Vitamin D, mcg | 0.0 (0.0, 0.1) | 0.0 (0.0, 0.2) | 0.0 (0.0, 0.0) | <0.001 |
| Vitamin E, mg | 0.3 (0.1, 0.6) | 0.3 (0.1, 0.6) | 0.7 (0.2, 1.4) | <0.001 |
| Vitamin K, mcg | 1.8 (0.4, 5.4) | 1.6 (0.4, 4.2) | 6.1 (1.0, 36.0) | <0.001 |
| Choline, mg | 12.4 (5.6, 26.9) | 11.3 (5.1, 25.4) | 19.2 (10.0, 35.9) | <0.001 |
| Minimally Processed Foods (N) | 2257 | 1628 | 629 |  |
| Vitamin A, mcg_RAE, | 16.4 (3.3, 64.2) | 14.2 (3.5, 47.8) | 31.9 (2.5, 134.9) | <0.001 |
| Thiamin, mg | 0.1 (0.0, 0.1) | 0.1 (0.0, 0.1) | 0.1 (0.1, 0.2) | <0.001 |
| Riboflavin, mg | 0.1 (0.1, 0.1) | 0.1 (0.1 0.1) | 0.1 (0.1, 0.2) | <0.001 |
| Niacin, mg | 1.2 (0.6, 2.1) | 1.2 (0.6, 2.3) | 1.0 (0.5, 1.7) | <0.001 |
| Vitamin B-6, mg | 0.1 (0.1, 0.2) | 0.1 (0.1, 0.2) | 0.1 (0.1, 0.3) | <0.001 |
| Folate, mcg_DFE | 16.5 (6.5, 39.7) | 13.4 (5.2, 27.9) | 36.0 (13.5, 83.3) | <0.001 |
| Vitamin B-12, mcg | 0.1 (0.0, 0.4) | 0.2 (0.0, 0.5) | 0.0 (0.0, 0.0) | <0.001 |
| Vitamin C, mg | 0.9 (0.0, 7.5) | 0.4 (0.0, 3.0) | 10.7 (1.2, 49.4) | <0.001 |
| Vitamin D, mcg | 0.0 (0.0,0.2) | 0.1 (0.0, 0.3) | 0.0 (0.0, 0.0) | <0.001 |
| Vitamin E, mg | 0.4 (0.2, 0.8) | 0.4 (0.2, 0.6) | 0.7 (0.3, 1.5) | <0.001 |
| Vitamin K, mcg | 3.0 (0.6, 9.0) | 2.4 (0.5, 5.7) | 7.1 (1.7, 43.3) | <0.001 |
| Choline, mg | 22.2 (10.7, 38.5) | 22.5 (10.6, 38.6) | 21.2 (11.0, 38.5) | 0.76 |
| Processed Culinary Ingredients (N) | 52 | 43 | 9 |  |
| Vitamin A, mcg_RAE, | 0.0 (0.0, 74.0) | 7.1 (0.0, 74.0) | 0 (0.0, 0.0) | - |
| Thiamin, mg | 0.0 (0.0, 0.0) | 0.0 (0.0, 0.0) | 0 (0.0, 0.0) | - |
| Riboflavin, mg | 0.0 (0.0, 0.1) | 0.0 (0.0, 0.1) | 0 (0.0, 0.0) | - |
| Niacin, mg | 0.0 (0.0, 0.1) | 0.0 (0.0, 0.1) | 0 (0.0, 0.0) | - |
| Vitamin B-6, mg | 0.0 (0.0, 0.0) | 0.0 (0.0, 0.0) | 0 (0.0, 0.0) | - |
| Folate, mcg_DFE | 0.5 (0.0, 3.4) | 0.7 (0.0, 5.1) | 0 (0.0, 0.0) | - |
| Vitamin B-12, mcg | 0.0 (0.0, 0.0) | 0.0 (0.0, 0.1) | 0 (0.0, 0.0) | - |
| Vitamin C, mg | 0.0 (0.0, 0.5) | 0.1 (0.0, 0.6) | 0 (0.0, 0.0) | - |
| Vitamin D, mcg | 0.0 (0.0, 0.0) | 0.0 (0.0, 0.0) | 0 (0.0, 0.0) | - |
| Vitamin E, mg | 0.1 (0.0, 0.3) | 0.1 (0.0, 0.2) | 1.6 (0.2, 2.0) | <0.001 |
| Vitamin K, mcg | 0.4 (0.0, 1.0) | 0.4 (0.0, 1.0) | 1.5 (0.6, 6.8) | 0.02 |
| Choline, mg | 2.6 (0.0, 7.1) | 2.7 (0.6, 8.9) | 0.0 (0.0, 0.0) | <0.001 |
| Processed Foods (N) | 325 | 277 | 48 |  |
| Vitamin A, mcg_RAE, | 7.4 (0.0, 47.2) | 10.0 (0.0, 48.4) | 2.2 (0.0, 26.1) | 0.25 |
| Thiamin, mg | 0.0 (0.0, 0.1) | 0.0 (0.0, 0.1) | 0.0 (0.0, 0.1) | 0.71 |
| Riboflavin, mg | 0.1 (0.0, 0.1) | 0.1 (0.0, 0.1) | 0.1 (0.0, 0.1) | 0.85 |
| Niacin, mg | 0.6 (0.1, 1.7) | 0.6 (0.1, 1.8) | 0.6 (0.3, 1.5) | 0.12 |
| Vitamin B-6, mg | 0.1 (0.0, 0.1) | 0.1 (0.0, 0.1) | 0.1 (0.0, 0.1) | 0.69 |
| Folate, mcg_DFE | 8.6 (2.7, 21.6) | 7.0 (2.4, 18.9) | 18.3 (8.8, 40.2) | <0.001 |
| Vitamin B-12, mcg | 0.0 (0.0, 0.3) | 0.1 (0.0, 0.4) | 0.0 (0.0, 0.0) | 0.06 |
| Vitamin C, mg | 0.1 (0.0, 3.8) | 0.1 (0.0, 3.8) | 0.3 (0.0, 4.0) | 0.04 |
| Vitamin D, mcg | 0.0 (0.0, 0.1) | 0.0 (0.0, 0.1) | 0.0 (0.0, 0.0) | 0.19 |
| Vitamin E, mg | 0.3 (0.1, 0.8) | 0.2 (0.1, 0.7) | 0.7 (0.4, 1.2) | <0.001 |
| Vitamin K, mcg | 0.7 (0.1, 3.5) | 0.7 (0.1, 3.4) | 1.8 (0.1, 4.9) | 0.27 |
| Choline, mg | 14.2 (5.8, 27.8) | 14.8 (4.9, 30.2) | 11.5 (8.5, 25.4) | 0.53 |
| Ultra-Processed Foods (N) | 2169 | 2123 | 46 |  |
| Vitamin A, mcg_RAE, | 5.5 (0.0, 31.7) | 5.6 (0.0, 31.9) | 0.0 (0.0, 4.5) | 0.76 |
| Thiamin, mg | 0.1 (0.0, 0.1) | 0.1 (0.0, 0.1) | 0.1 (0.0, 0.2) | 0.02 |
| Riboflavin, mg | 0.1 (0.0, 0.1) | 0.1 (0.0, 0.1) | 0.0 (0.0, 0.2) | 0.15 |
| Niacin, mg | 0.9 (0.3, 1.7) | 0.9 (0.3, 1.6) | 1.1 (0.5, 2.0) | 0.05 |
| Vitamin B-6, mg | 0.0 (0.0, 0.1) | 0.0 (0.0, 0.1) | 0.1 (0.1, 0.2) | <0.001 |
| Folate, mcg_DFE | 14.5 (3.5, 32.4) | 14.4 (3.4, 32.5) | 15.3 (7.7, 25.4) | 0.25 |
| Vitamin B-12, mcg | 0.1 (0.0, 0.2) | 0.1 (0.0, 0.2) | 0.0 (0.0, 0.2) | <0.01 |
| Vitamin C, mg | 0.1 (0.0, 1.2) | 0.1 (0.0, 1.2) | 0.0 (0.0, 3.1) | 0.99 |
| Vitamin D, mcg | 0.0 (0.0, 0.1) | 0.0 (0.0, 0.1) | 0.0 (0.0, 0.0) | 0.21 |
| Vitamin E, mg | 0.2 (0.1, 0.5) | 0.2 (0.1, 0.5) | 0.3 (0.1, 1.0) | 0.03 |
| Vitamin K, mcg | 1.2 (0.3, 3.1) | 1.2 (0.3, 3.1) | 1.1 (0.4, 3.6) | 0.45 |
| Choline, mg | 7.3 (3.4, 14.4) | 7.3 (3.3, 14.4) | 7.8 (6.0, 13.3) | 0.05 |

1. Abbreviations: grams (g), milligrams (mg), microgram (mcg), interquartile range (IQR), retinol activity equivalent (RAE), dietary folate equivalent (DFE)
2. P-values from t-tests comparing mean log-transformed nutrient values between FDA-aligned and FDA-unaligned groups

**Supplemental Table 12.** Mean Vitamins per 100kcal of FDA-Aligned and FDA-Unaligned Foods and Beverages Consumed in the United States NHANES/FNDDS 2017-2018, Overall and Stratified by Food Category^1^

|  | **Overall** | **FDA Unaligned** | **FDA Aligned** | **p-value^2^** |
| --- | --- | --- | --- | --- |
| Beverages (N) | 422 | 324 | 98 |  |
| Vitamin A, mcg_RAE, | 0.0 (0.0, 60.9) | 0.0 (0.0, 67.0) | 3.9 (0.0, 39.6) | <0.001 |
| Thiamin, mg | 0.0 (0.0, 0.1) | 0.0 (0.0, 0.1) | 0.1 (0.0, 0.2) | <0.001 |
| Riboflavin, mg | 0.0 (0.0, 0.2) | 0.0 (0.0, 0.2) | 0.1 (0.0, 0.4) | <0.001 |
| Niacin, mg | 0.3 (0.0, 1.7) | 0.2 (0.0, 1.8) | 0.6 (0.2, 1.2) | 0.03 |
| Vitamin B-6, mg | 0.0 (0.0, 0.1) | 0.0 (0.0, 0.1) | 0.1 (0.0, 0.2) | <0.01 |
| Folate, mcg_DFE | 1.8 (0.0, 16.1) | 0.9 (0.0, 7.5) | 27.3 (5.3, 70.5) | <0.001 |
| Vitamin B-12, mcg | 0.0 (0.0, 0.4) | 0.0 (0.0, 0.5) | 0.0 (0.0, 0.0) | 0.46 |
| Vitamin C, mg | 0.2 (0.0, 19.1) | 0.0 (0.0, 10.6) | 16.9 (0.0, 56.4) | <0.001 |
| Vitamin D, mcg | 0.0 (0.0, 0.2) | 0.0 (0.0, 0.3) | 0.0 (0.0, 0.0) | <0.001 |
| Vitamin E, mg | 0.0 (0.0, 0.3) | 0.0 (0.0, 0.2) | 0.3 (0.0, 0.7) | 0.76 |
| Vitamin K, mcg | 0.0 (0.0, 0.7) | 0.0 (0.0, 0.5) | 0.4 (0.0, 8.1) | <0.001 |
| Choline, mg | 4.8 (0.2, 19.4) | 2.6 (0.0, 17.3) | 16.3 (6.2, 33.1) | <0.001 |
| Grains (N) | 437 | 416 | 21 |  |
| Vitamin A, mcg_RAE, | 7.7 (0.0, 110.4) | 8.7 (0.0, 114.9) | 0.0 (0.0, 0.0) | 0.64 |
| Thiamin, mg | 0.2 (0.1, 0.3) | 0.2 (0.1, 0.3) | 0.1 (0.1, 0.2) | 0.03 |
| Riboflavin, mg | 0.1 (0.1, 0.2) | 0.1 (0.1, 0.2) | 0.0 (0.0, 0.1) | 0.01 |
| Niacin, mg | 1.5 (0.9, 2.6) | 1.6 (1.0, 2.7) | 1.1 (0.3, 1.5) | <0.01 |
| Vitamin B-6, mg | 0.1 (0.0, 0.3) | 0.1 (0.0, 0.3) | 0.1 (0.0, 0.1) | 0.28 |
| Folate, mcg_DFE | 48.6 (21.7, 101.2) | 50.6 (26.3, 128.7)) | 12.2 (6.4, 24.1) | <0.001 |
| Vitamin B-12, mcg | 0.0 (0.0, 0.5) | 0.0 (0.0, 0.6) | 0.0 (0.0, 0.0) | 0.14 |
| Vitamin C, mg | 0.0 (0.0, 0.5) | 0.0 (0.0, 0.5) | 0.0 (0.0, 0.0) | 0.04 |
| Vitamin D, mcg | 0.0 (0.0, 0.6) | 0.0 (0.0, 0.7) | 0.0 (0.0, 0.0) | 0.64 |
| Vitamin E, mg | 0.2 (0.1, 0.3) | 0.2 (0.1, 0.3) | 0.1 (0.1, 0.2) | 0.52 |
| Vitamin K, mcg | 0.6 (0.3, 1.6) | 0.6 (0.3, 1.6) | 0.5 (0.5, 0.6) | 0.35 |
| Choline, mg | 5.4 (3.4, 7.8) | 5.3 (3.2, 7.5) | 8.9 (6.8, 10.7) | <0.001 |
| Vegetables (N) | 525 | 212 | 313 |  |
| Vitamin A, mcg_RAE, | 57.8 (14.4, 293.2) | 30.8 (3.1, 76.9) | 91.3 (23.6, 456.1) | <0.001 |
| Thiamin, mg | 0.1 (0.1, 0.2) | 0.1 (0.0, 0.1) | 0.1 (0.1, 0.2) | <0.001 |
| Riboflavin, mg | 0.1 (0.0, 0.2) | 0.0 (0.0, 0.1) | 0.1 (0.1, 0.3) | <0.001 |
| Niacin, mg | 1.1 (0.7, 1.6) | 0.8 (0.6, 1.1) | 1.3 (0.9, 1.9) | <0.001 |
| Vitamin B-6, mg | 0.2 (0.1, 0.3) | 0.1 (0.1, 0.2) | 0.2 (0.1, 0.4) | <0.001 |
| Folate, mcg_DFE | 37.4 (12.5, 93.1) | 11.2 (7.1, 32.1) | 59.3 (31.6, 121.3) | <0.001 |
| Vitamin B-12, mcg | 0.0 (0.0, 0.0) | 0.0 (0.0, 0.0) | 0.0 (0.0, 0.0) | <0.01 |
| Vitamin C, mg | 10.5 (5.4, 42.4) | 6.4 (3.4, 10.5) | 21.7 (8.6, 76.1) | <0.001 |
| Vitamin D, mcg | 0.0 (0.0, 0.0) | 0.0 (0.0, 0.0) | 0.0 (0.0, 0.0) | <0.001 |
| Vitamin E, mg | 0.8 (0.4, 1.6) | 0.6 (0.4, 0.8) | 1.0 (0.6, 2.0) | <0.001 |
| Vitamin K, mcg | 20.4 (4.5, 97.5) | 5.7 (2.5, 15.3) | 36.9 (12.4, 173.8) | <0.001 |
| Choline, mg | 19.9 (11.3, 35.7) | 11.5 (9.6, 15.5) | 28.3 (17.3, 41.4) | <0.001 |
| Fruit (N) | 110 | 43 | 67 |  |
| Vitamin A, mcg_RAE, | 6.6 (2.1, 45.2) | 4.2 (1.1, 34.0) | 6.7 (3.4, 53.8) | 0.18 |
| Thiamin, mg | 0.0 (0.0, 0.1) | 0.0 (0.0, 0.0) | 0.1 (0.0, 0.1) | 0.01 |
| Riboflavin, mg | 0.1 (0.0, 0.1) | 0.0 (0.0, 0.1) | 0.1 (0.0, 0.1) | <0.001 |
| Niacin, mg | 0.6 (0.3, 0.9) | 0.4 (0.1, 0.6) | 0.7 (0.3, 1.2) | <0.001 |
| Vitamin B-6, mg | 0.1 (0.1, 0.1) | 0.1 (0.0, 0.1) | 0.1 (0.1, 0.1) | <0.001 |
| Folate, mcg_DFE | 10.7 (3.8, 30.2) | 6.3 (2.2, 11.5) | 15.4 (6.7, 40.4) | <0.001 |
| Vitamin B-12, mcg | 0.0 (0.0, 0.0) | 0.0 (0.0, 0.0) | 0.0 (0.0, 0.0) | 0.54 |
| Vitamin C, mg | 10.6 (2.7, 50.0) | 5.7 (2.5, 14.6) | 13.1 (4.8, 60.7) | 0.01 |
| Vitamin D, mcg | 0.0 (0.0, 0.0) | 0.0 (0.0, 0.0) | 0.0 (0.0, 0.0) | - |
| Vitamin E, mg | 0.3 (0.1, 0.8) | 0.3 (0.2, 0.7) | 0.4 (0.1, 0.9) | 0.32 |
| Vitamin K, mcg | 3.1 (0.9, 7.0) | 1.8 (0.6, 4.1) | 5.1 (1.2, 7.9) | <0.01 |
| Choline, mg | 8.2 (5.5, 12.9) | 6.2 (4.4, 8.4) | 11.0 (6.3, 14.5) | <0.001 |
| Legumes (N) | 80 | 45 | 35 |  |
| Vitamin A, mcg_RAE, | 0.0 (0.0, 0.6) | 0.0 (0.0, 1.0) | 0.0 (0.0, 0.5) | <0.01 |
| Thiamin, mg | 0.1 (0.1, 0.1) | 0.1 (0.1, 0.1) | 0.1 (0.1, 0.1) | 0.46 |
| Riboflavin, mg | 0.0 (0.0, 0.1) | 0.0 (0.0, 0.1) | 0.0 (0.0, 0.0) | 0.11 |
| Niacin, mg | 0.3 (0.2, 0.6) | 0.4 (0.3, 0.7) | 0.3 (0.2, 0.4) | 0.01 |
| Vitamin B-6, mg | 0.1 (0.1, 0.1) | 0.1 (0.1, 0.1) | 0.1 (0.0, 0.1) | 0.08 |
| Folate, mcg_DFE | 56.1 (25.0, 74.4) | 33.7 (19.8, 64.3) | 73.3 (44.5, 97.0) | <0.001 |
| Vitamin B-12, mcg | 0.0 (0.0, 0.0) | 0.0 (0.0, 0.0) | 0.0 (0.0, 0.0) | - |
| Vitamin C, mg | 0.3 (0.0, 0.8) | 0.3 (0.0, 1.0) | 0.3 (0.0, 0.6) | 0.06 |
| Vitamin D, mcg | 0.0 (0.0, 0.0) | 0.0 (0.0, 0.0) | 0.0 (0.0, 0.0) | - |
| Vitamin E, mg | 0.6 (0.4, 0.9) | 0.7 (0.2, 0.9) | 0.6 (0.4, 0.9) | 0.37 |
| Vitamin K, mcg | 5.4 (2.5, 5.8) | 3.5 (0.9, 5.7) | 5.6 (2.5, 5.8) | 0.14 |
| Choline, mg | 18.9 (17.2, 25.4) | 21.3 (17.8, 26.0) | 18.1 (17.1, 24.7) | 0.76 |
| Nuts and Seeds (N) | 77 | 24 | 53 |  |
| Vitamin A, mcg_RAE, | 0.0 (0.0, 0.2) | 0.0 (0.0, 0.0) | 0.0 (0.0, 0.2) | 0.02 |
| Thiamin, mg | 0.0 (0.0, 0.1) | 0.0 (0.0, 0.0) | 0.0 (0.0, 0.1) | <0.01 |
| Riboflavin, mg | 0.0 (0.0, 0.1) | 0.0 (0.0, 0.0) | 0.0 (0.0, 0.1) | 0.07 |
| Niacin, mg | 0.6 (0.2, 1.3) | 1.1 (0.2, 1.9) | 0.6 (0.2, 1.3) | 0.35 |
| Vitamin B-6, mg | 0.1 (0.0, 0.1) | 0.0 (0.0, 0.1) | 0.1 (0.0, 0.1) | 0.28 |
| Folate, mcg_DFE | 11.5 (8.8, 16.5) | 10.6 (8.9, 17.3) | 11.6 (8.7, 16.5) | 0.82 |
| Vitamin B-12, mcg | 0.0 (0.0, 0.0) | 0.0 (0.0, 0.0) | 0.0 (0.0, 0.0) | - |
| Vitamin C, mg | 0.1 (0.0, 0.2) | 0.0 (0.0, 0.2) | 0.1 (0.0, 0.2) | 0.51 |
| Vitamin D, mcg | 0.0 (0.0, 0.0) | 0.0 (0.0, 0.0) | 0.0 (0.0, 0.0) | - |
| Vitamin E, mg | 1.1 (0.2, 1.6) | 1.1 (0.6, 2.3) | 0.9 (0.2, 1.6) | 0.14 |
| Vitamin K, mcg | 0.6 (0.1, 2.2) | 0.5 (0.1, 1.8) | 0.8 (0.0, 2.3) | <0.01 |
| Choline, mg | 9.2 (7.3, 10.4) | 8.2 (6.6, 10.4) | 9.2 (8.2, 10.6) | 0.45 |
| Meat (N) | 472 | 458 | 14 |  |
| Vitamin A, mcg_RAE, | 3.2 (0.4, 6.3) | 3.1 (0.5, 6.2) | 50.9 (0.0, 111.9) | <0.0001 |
| Thiamin, mg | 0.0 (0.0, 0.1) | 0.0 (0.0, 0.1) | 0.0 (0.0, 0.1) | 0.16 |
| Riboflavin, mg | 0.1 (0.1, 0.1) | 0.1 (0.1, 0.1) | 0.3 (0.3, 0.3) | <0.0001 |
| Niacin, mg | 2.6 (1.7, 3.9) | 2.6 (1.7, 3.9) | 0.1 (0.0, 3.9) | <0.0001 |
| Vitamin B-6, mg | 0.2 (0.1, 0.3) | 0.2 (0.1, 0.3) | 0.1 (0.1, 0.3) | 0.11 |
| Folate, mcg_DFE | 3.5 (1.8, 6.5) | 3.4 (1.8, 6.2) | 17.7 (5.8, 28.6) | <0.0001 |
| Vitamin B-12, mcg | 0.3 (0.2, 0.6) | 0.3 (0.2, 0.5) | 0.6 (0.5, 1.6) | <0.01 |
| Vitamin C, mg | 0.0 (0.0, 0.0) | 0.0 (0.0, 0.0) | 0.0 (0.0, 0.0) | - |
| Vitamin D, mcg | 0.1 (0.0, 0.2) | 0.1 (0.0, 0.2) | 0.5 (0.0, 1.4) | <0.0001 |
| Vitamin E, mg | 0.2 (0.1, 0.4) | 0.2 (0.1, 0.3) | 0.6 (0.4, 0.7) | <0.0001 |
| Vitamin K, mcg | 0.6 (0.0, 1.9) | 0.7 (0.0 2.0) | 0.2 (0.2, 0.8) | <0.01 |
| Choline, mg | 35.4 (25.2, 47.3) | 35.4 (25.1, 46.7) | 106.7 (70.2, 164.3) | <0.001 |
| Seafood (N) | 193 | 176 | 17 |  |
| Vitamin A, mcg_RAE, | 15.4 (8.7, 26.9) | 15.0 (7.7, 25.0) | 25.5 (16.5, 41.9) | 0.01 |
| Thiamin, mg | 0.1 (0.0, 0.1) | 0.1 (0.0, 0.1) | 0.0 (0.0, 0.1) | 0.06 |
| Riboflavin, mg | 0.1 (0.1, 0.1) | 0.1 (0.1, 0.1) | 0.1 (0.0, 0.1) | 0.50 |
| Niacin, mg | 1.8 (1.2, 3.3) | 1.8 (1.2, 3.3) | 1.7 (0.9, 2.8) | 0.39 |
| Vitamin B-6, mg | 0.1 (0.1, 0.2) | 0.1 (0.1, 0.2) | 0.1 (0.0, 0.2) | 0.03 |
| Folate, mcg_DFE | 9.9 (6.0, 15.1) | 10.2 (6.2, 15.6) | 8.3 (4.4, 13.7) | 0.56 |
| Vitamin B-12, mcg | 1.4 (0.8, 2.4) | 1.4 (0.8, 2.2) | 2.6 (0.9, 3.8) | 0.03 |
| Vitamin C, mg | 0.3 (0.0, 0.5) | 0.3 (0.0, 0.5) | 0.0 (0.0, 0.4) | 0.15 |
| Vitamin D, mcg | 1.2 (0.1, 3.4) | 1.2 (0.2, 3.9) | 0.1 (0.0, 0.2) | 0.04 |
| Vitamin E, mg | 0.7 (0.5, 1.0) | 0.7 (0.5, 1.0) | 0.9 (0.7, 1.3) | 0.17 |
| Vitamin K, mcg | 2.0 (0.3, 4.0) | 2.0 (0.3, 4.0) | 2.0 (0.2, 3.4) | 0.87 |
| Choline, mg | 47.4 (35.5, 68.2) | 46.2 (35.1, 67.6) | 58.9 (54.2, 74.5) | 0.03 |
| Dairy (N) | 196 | 174 | 22 |  |
| Vitamin A, mcg_RAE, | 62.1 (29.5, 87.9) | 59.7 (31.8, 82.1) | 123.3 (22.2, 180.4) | 0.33 |
| Thiamin, mg | 0.0 (0.0, 0.1) | 0.0 (0.0, 0.1) | 0.1 (0.1, 0.1) | <0.001 |
| Riboflavin, mg | 0.2 (0.1, 0.3) | 0.2 (0.1, 0.2) | 0.3 (0.3, 0.4) | <0.001 |
| Niacin, mg | 0.1 (0.0, 0.2) | 0.1 (0.0, 0.2) | 0.3 (0.2, 0.3) | <0.001 |
| Vitamin B-6, mg | 0.0 (0.0, 0.1) | 0.0 (0.0, 0.1) | 0.1 (0.1, 0.1) | <0.001 |
| Folate, mcg_DFE | 4.1 (2.2, 8.7) | 3.5 (2.0, 8.0) | 13.2 (5.9, 17.5) | <0.001 |
| Vitamin B-12, mcg | 0.5 (0.3, 0.7) | 0.5 (0.3, 0.7) | 1.1 (0.9, 1.1) | <0.001 |
| Vitamin C, mg | 0.0 (0.0, 1.1) | 0.0 (0.0, 0.7) | 1.3 (0.0, 1.6) | 0.10 |
| Vitamin D, mcg | 0.7 (0.1, 1.6) | 0.2 (0.1, 1.4) | 1.9 (0.1, 2.6) | <0.001 |
| Vitamin E, mg | 0.1 (0.0, 0.1) | 0.1 (0.0, 0.1) | 0.0 (0.0, 0.0) | <0.01 |
| Vitamin K, mcg | 0.4 (0.2, 0.6) | 0.4 (0.2, 0.7) | 0.2 (0.0, 0.3) | 0.02 |
| Choline, mg | 17.9 (4.7, 24.4) | 15.3 (4.4, 23.7) | 30.8 (24.1, 46.7) | <0.001 |
| Fats and Oils (N) | 68 | 60 | 8 |  |
| Vitamin A, mcg_RAE, | 3.2 (0.9, 93.2) | 4.0 (1.2, 93.4) | 0.0 (0.0, 0.0) | - |
| Thiamin, mg | 0.0 (0.0, 0.0) | 0.0 (0.0, 0.0) | 0.0 (0.0, 0.0) | - |
| Riboflavin, mg | 0.0 (0.0, 0.0) | 0.0 (0.0, 0.0) | 0.0 (0.0, 0.0) | - |
| Niacin, mg | 0.0 (0.0, 0.0) | 0.0 (0.0, 0.0) | 0.0 (0.0, 0.0) | - |
| Vitamin B-6, mg | 0.0 (0.0, 0.0) | 0.0 (0.0, 0.0) | 0.0 (0.0, 0.0) | - |
| Folate, mcg_DFE | 0.5 (0.0, 1.6) | 0.7 (0.2, 2.1) | 0.0 (0.0, 0.0) | - |
| Vitamin B-12, mcg | 0.0 (0.0, 0.0) | 0.0 (0.0, 0.0) | 0.0 (0.0, 0.0) | - |
| Vitamin C, mg | 0.0 (0.0, 0.1) | 0.0 (0.0, 0.1) | 0.0 (0.0, 0.0) | - |
| Vitamin D, mcg | 0.0 (0.0, 0.0) | 0.0 (0.0, 0.0) | 0.0 (0.0, 0.0) | - |
| Vitamin E, mg | 0.8 (0.4, 1.5) | 0.7 (0.4, 1.2) | 1.6 (0.7, 3.2) | 0.40 |
| Vitamin K, mcg | 8.0 (1.0, 18.2) | 12.3 (1.0, 19.0) | 1.6 (0.7, 7.4) | 0.08 |
| Choline, mg | 2.6 (1.8, 5.3) | 3.2 (1.9, 6.1) | 0.0 (0.0, 0.0) | <0.001 |
| Mixed Dishes (N) | 1510 | 1441 | 69 |  |
| Vitamin A, mcg_RAE, | 21.8 (6.9, 45.7) | 21.8 (7.2, 45.6) | 19.2 (5.0, 83.7) | 0.08 |
| Thiamin, mg | 0.1 (0.0, 0.1) | 0.1 (0.1, 0.1) | 0.1 (0.0, 0.1) | 0.70 |
| Riboflavin, mg | 0.1 (0.1, 0.1) | 0.1 (0.1, 0.1) | 0.1 (0.1, 0.1) | 0.68 |
| Niacin, mg | 1.2 (0.8, 1.8) | 1.2 (0.8, 1.7) | 1.4 (0.8, 1.9) | 0.97 |
| Vitamin B-6, mg | 0.1 (0.1, 0.1) | 0.1 (0.1, 0.1) | 0.1 (0.1, 0.1) | <0.001 |
| Folate, mcg_DFE | 21.5 (12.7, 35.1) | 21.8 (12.9, 35.6) | 17.3 (11.0, 21.4) | 0.21 |
| Vitamin B-12, mcg | 0.2 (0.1, 0.4) | 0.2 (0.1, 0.4) | 0.0 (0.0, 0.2) | 0.43 |
| Vitamin C, mg | 0.7 (0.1, 3.0) | 0.7 (0.1, 2.9) | 3.6 (0.6, 9.0) | <0.0001 |
| Vitamin D, mcg | 0.1 (0.0, 0.2) | 0.1 (0.0, 0.2) | 0.0 (0.0, 0.0) | <0.01 |
| Vitamin E, mg | 0.4 (0.2, 0.6) | 0.4 (0.2, 0.6) | 0.6 (0.4, 1.1) | <0.0001 |
| Vitamin K, mcg | 3.4 (1.6, 7.3) | 3.3 (1.5, 7.2) | 5.6 (3.6, 15.5) | <0.0001 |
| Choline, mg | 15.6 (9.0, 26.3) | 15.6 (9.0, 26.3) | 11.1 (8.8, 25.9) | 0.08 |
| Sauces and Condiments (N) | 178 | 169 | 9 |  |
| Vitamin A, mcg_RAE, | 7.0 (0.0, 38.3) | 7.1 (0.0, 40.9) | 4.8 (0.0, 8.0) | 0.56 |
| Thiamin, mg | 0.0 (0.0, 0.1) | 0.0 (0.0, 0.1) | 0.1 (0.1, 0.2) | <0.01 |
| Riboflavin, mg | 0.1 (0.0, 0.1) | 0.0 (0.0, 0.1) | 0.1 (0.1, 0.1) | 0.37 |
| Niacin, mg | 0.2 (0.0, 1.0) | 0.2 (0.0, 1.0) | 0.6 (0.5, 1.0) | 0.11 |
| Vitamin B-6, mg | 0.0 (0.0, 0.2) | 0.0 (0.0, 0.2) | 0.2 (0.2, 0.2) | <0.01 |
| Folate, mcg_DFE | 5.3 (1.1, 20.7) | 4.4 (1.1, 17.1) | 40.0 (38.1, 73.5) | 0.01 |
| Vitamin B-12, mcg | 0.0 (0.0, 0.0) | 0.0 (0.0, 0.0) | 0.0 (0.0, 0.0) | - |
| Vitamin C, mg | 0.5 (0.0, 7.2) | 0.4 (0.0, 6.2) | 120.0 (30.5, 175.9) | <0.001 |
| Vitamin D, mcg | 0.0 (0.0, 0.0) | 0.0 (0.0, 0.0) | 0.0 (0.0, 0.0) | - |
| Vitamin E, mg | 0.4 (0.0, 0.9) | 0.4 (0.0, 0.9) | 0.7 (0.6, 0.9) | 0.23 |
| Vitamin K, mcg | 1.5 (0.2, 14.5) | 1.4 (0.2, 14.5) | 2.4 (0.6, 2.4) | 0.92 |
| Choline, mg | 7.6 (1.9, 16.4) | 6.9 (1.7, 13.7) | 23.2 (20.4, 24.3) | 0.03 |
| Snacks and Desserts (N) | 680 | 671 | 9 |  |
| Vitamin A, mcg_RAE, | 1.7 (0.0, 13.2) | 1.7 (0.0, 13.5) | 0.0 (0.0, 1.0) | 0.53 |
| Thiamin, mg | 0.0 (0.0, 0.1) | 0.0 (0.0, 0.1) | 0.0 (0.0, 0.0) | 0.67 |
| Riboflavin, mg | 0.1 (0.0, 0.1) | 0.1 (0.0, 0.1) | 0.0 (0.0, 0.0) | 0.45 |
| Niacin, mg | 0.4 (0.2, 0.7) | 0.4 (0.2, 0.7) | 1.1 (0.6, 1.6) | 0.01 |
| Vitamin B-6, mg | 0.0 (0.0, 0.0) | 0.0 (0.0, 0.0) | 0.1 (0.0, 0.1) | <0.01 |
| Folate, mcg_DFE | 10.0 (3.5, 20.6) | 10.2 (3.5, 20.7) | 7.7 (5.5, 8.1) | 0.65 |
| Vitamin B-12, mcg | 0.0 (0.0, 0.1) | 0.0 (0.0, 0.1) | 0.0 (0.0, 0.0) | - |
| Vitamin C, mg | 0.0 (0.0, 0.3) | 0.0 (0.0, 0.3) | 0.0 (0.0, 0.0) | - |
| Vitamin D, mcg | 0.0 (0.0, 0.0) | 0.0 (0.0, 0.0) | 0.0 (0.0, 0.0) | - |
| Vitamin E, mg | 0.3 (0.1, 0.5) | 0.3 (0.1, 0.5) | 0.3 (0.2, 0.3) | 0.59 |
| Vitamin K, mcg | 1.1 (0.4, 2.4) | 1.1 (0.4, 2.4) | 1.7 (0.5, 3.0) | 0.52 |
| Choline, mg | 5.0 (2.5, 8.0) | 4.9 (2.5, 8.0) | 6.0 (5.5, 6.0) | 0.44 |

1. Abbreviations: grams (g), milligrams (mg), microgram (mcg), interquartile range (IQR), retinol activity equivalent (RAE), dietary folate equivalent (DFE)
2. P-values from t-tests comparing mean log-transformed nutrient values between FDA-aligned and FDA-unaligned groups

**Supplemental Table 13.** Mean Minerals per 100kcal of FDA-Aligned and FDA-Unaligned Foods and Beverages Consumed in the United States NHANES/FNDDS 2017-2018, Overall and Stratified by Nova^1^

|  | **Overall**  **Median (IQR)** | **FDA Unaligned**  **Median (IQR)** | **FDA Aligned**  **Median (IQR)** | **p-value^2^** |
| --- | --- | --- | --- | --- |
| Overall (N) | 4949 | 4214 | 735 |  |
| Calcium, mg | 23.1 (9.0, 59.0) | 20.9 (8.4, 55.4) | 37.8 (15.8, 84.2) | <0.001 |
| Phosphorus, mg | 65.7 (36.9, 99.6) | 63.4 (35.8, 97.0) | 78.2 (48.3, 111.4) | <0.001 |
| Magnesium, mg | 12.5 (7.6, 21.3) | 11.1 (7.1, 17.4) | 29.7 (21.3, 45.4) | <0.001 |
| Iron, mg | 0.6 (0.3, 1.0) | 0.6 (0.3, 0.9) | 0.9 (0.5, 1.7) | <0.001 |
| Zinc, mg | 0.5 (0.2, 0.8) | 0.4 (0.2, 0.8) | 0.6 (0.4, 0.9) | <0.001 |
| Copper, mg | 0.0 (0.0, 0.1) | 0.0 (0.0, 0.1) | 0.1 (0.1, 0.2) | <0.001 |
| Selenium, mcg | 4.4 (1.3, 9.2) | 5.0 (1.7, 9.7) | 1.5 (0.6, 4.3) | <0.001 |
| Potassium, mg | 107.0 (58.9, 207.9) | 93.6 (51.7, 166.7) | 306.1 (168.2, 496.9) | <0.001 |
| Sodium, mg | 172.9 (92.2, 256.0) | 176.5 (103.6, 256.2) | 132.9 (22.7, 252.2) | <0.001 |
| Minimally Processed Foods (N) | 2257 | 1628 | 629 |  |
| Calcium, mg | 21.2 (9.7, 57.0) | 16.7 (8.5, 43.7) | 40.3 (17.4, 94.6) | <0.001 |
| Phosphorus, mg | 80.9 (50.4, 115.7) | 81.1 (50.9, 115.3) | 79.7 (50.0, 120.2) | 0.16 |
| Magnesium, mg | 15.5 (10.2, 26.6) | 12.6 (9.1, 18.6) | 30.4 (22.1, 46.8) | <0.001 |
| Iron, mg | 0.7 (0.4, 1.0) | 0.6 (0.4, 0.9) | 1.0 (0.5, 1.8) | <0.001 |
| Zinc, mg | 0.6 (0.3, 0.9) | 0.6 (0.3, 0.9) | 0.6 (0.4, 1.0) | 0.81 |
| Copper, mg | 0.1 (0.0, 0.1) | 0.0 (0.0,0.1) | 0.1 (0.1, 0.2) | <0.001 |
| Selenium, mcg | 5.3 (2.0, 12.5) | 7.7 (3.7, 13.8) | 1.5 (0.6, 4.1) | <0.001 |
| Potassium, mg | 159.8 (95.1, 287.8) | 133.2 (79.3, 205.0) | 340.0 (203.4, 539.6) | <0.001 |
| Sodium, mg | 181.7 (123.5, 258.8) | 192.5 (143.9, 258.0) | 137.8 (23.3, 261.4) | <0.001 |
| Processed Culinary Ingredients (N) | 52 | 43 | 9 |  |
| Calcium, mg | 3.3 (0.3, 40.3) | 6.1 (1.6, 46.7) | 0.0 (0.0, 0.0) | 0.97 |
| Phosphorus, mg | 3.3 (0.0, 38.6) | 3.4 (1.3, 43.5) | 0.0 (0.0, 0.0) | - |
| Magnesium, mg | 0.6 (0.0, 7.6) | 2.1 (0.3, 7.6) | 0.0 (0.0, 0.0) | - |
| Iron, mg | 0.0 (0.0, 0.2) | 0.0 (0.0, 0.2) | 0.0 (0.0, 0.0) | 0.88 |
| Zinc, mg | 0.0 (0.0, 0.2) | 0.0 (0.0, 0.2) | 0.0 (0.0, 0.0) | - |
| Copper, mg | 0.0 (0.0, 0.0) | 0.0 (0.0, 0.0) | 0.0 (0.0, 0.0) | - |
| Selenium, mcg | 0.3 (0.1, 2.4) | 1.0 (0.2, 2.4) | 0.0 (0.0, 0.0) | - |
| Potassium, mg | 10.1 (0.5, 82.0) | 24.0 (3.3, 83.1) | 0.0 (0.0, 0.0) | 0.51 |
| Sodium, mg | 23.8 (0.5, 79.1) | 35.1 (6.5, 79.1) | 0.0 (0.0, 0.0) | 0.46 |
| Processed Foods (N) | 325 | 277 | 48 |  |
| Calcium, mg | 28.1 (7.7, 76.9) | 29.7 (7.2, 86.7) | 23.7 (9.9, 42.9) | 0.15 |
| Phosphorus, mg | 81.5 (41.3, 120.0) | 85.2 (38.1, 127.3) | 67.5 (52.5, 82.7) | 0.79 |
| Magnesium, mg | 14.8 (8.0, 28.3) | 13.0 (7.5, 24.2) | 28.2 (18.6, 32.6) | <0.001 |
| Iron, mg | 0.6 (0.2, 1.0) | 0.5 (0.2, 1.0) | 0.6 (0.4, 1.4) | 0.02 |
| Zinc, mg | 0.5 (0.3, 0.9) | 0.6 (0.2, 1.0) | 0.5 (0.4, 0.7) | 0.23 |
| Copper, mg | 0.1 (0.0, 0.1) | 0.1 (0.0, 0.1) | 0.1 (0.1, 0.2) | <0.001 |
| Selenium, mcg | 3.5 (0.8, 9.4) | 3.9 (0.9, 9.9) | 1.2 (0.6, 1.9) | <0.001 |
| Potassium, mg | 120.4 (67.5, 225.4) | 115.7 (56.6, 225.4) | 123.8 (108.0, 223.3) | 0.05 |
| Sodium, mg | 229.9 (48.4, 411.1) | 237.5 (62.9, 425.9) | 67.0 (33.2, 280.3) | <0.01 |
| Ultra-Processed Foods (N) | 2169 | 2123 | 46 |  |
| Calcium, mg | 23.9 (8.2, 58.7) | 23.7 (8.0, 58.6) | 35.5 (16.6, 66.5) | 0.09 |
| Phosphorus, mg | 49.7 (29.0, 79.0) | 49.5 (28.8, 78.2) | 80.2 (41.9, 96.6) | <0.001 |
| Magnesium, mg | 9.5 (6.1, 16.6) | 9.4 (6.0, 15.9) | 29.6 (21.3, 36.2) | <0.001 |
| Iron, mg | 0.6 (0.3, 1.0) | 0.6 (0.3, 1.0) | 0.7 (0.4, 1.0) | 0.08 |
| Zinc, mg | 0.4 (0.2, 0.6) | 0.4 (0.2, 0.6) | 0.6 (0.3, 0.9) | <0.001 |
| Copper, mg | 0.0 (0.0, 0.1) | 0.0 (0.0, 0.1) | 0.1 (0.1, 0.1) | <0.001 |
| Selenium, mcg | 3.7 (1.0, 7.5) | 3.7 (1.0, 7.5) | 5.8 (0.8, 10.2) | 0.07 |
| Potassium, mg | 73.2 (42.7, 125.7) | 71.3 (42.2, 123.7) | 142.0 (87.2, 212.5) | <0.001 |
| Sodium, mg | 153.4 (76.1, 240.0) | 155.4 (77.2, 241.9) | 96.7 (44.3, 138.5) | <0.01 |

1. Abbreviations: milligrams (mg), microgram (mcg), interquartile range (IQR)
2. P-values from t-tests comparing mean log-transformed nutrient values between FDA-aligned and FDA-unaligned groups

**Supplemental Table 14.** Mean Minerals per 100kcal of FDA-Aligned and FDA-Unaligned Foods and Beverages Consumed in the United States NHANES/FNDDS 2017-2018, Overall and Stratified by Food Category^1^

|  | **Overall** | **FDA Unaligned** | **FDA Aligned** | **p-value^2^** |
| --- | --- | --- | --- | --- |
| Beverages (N) | 422 | 324 | 98 |  |
| Calcium, mg | 26.1 (5.9, 132.1) | 22.2 (4.7, 125.7) | 49.0 (20.4, 133.2) | <0.001 |
| Phosphorus, mg | 30.8 (5.2, 100.0) | 26.1 (4.1, 86.4) | 41.6 (21.6, 107.9) | <0.001 |
| Magnesium, mg | 16.9 (4.3, 47.8) | 13.5 (1.9, 41.6) | 31.8 (19.6, 86.3) | <0.001 |
| Iron, mg | 0.2 (0.0, 0.7) | 0.1 (0.0, 0.6) | 0.5 (0.2, 1.0) | <0.001 |
| Zinc, mg | 0.2 (0.0, 0.5) | 0.1 (0.0, 0.5) | 0.3 (0.1, 1.0) | <0.001 |
| Copper, mg | 0.0 (0.0, 0.1) | 0.0 (0.0, 0.1) | 0.1 (0.0, 0.2) | <0.001 |
| Selenium, mcg | 0.3 (0.0, 1.9) | 0.4 (0.0, 2.0) | 0.2 (0.0, 1.5) | 0.54 |
| Potassium, mg | 162.5 (40.6, 363.9) | 112.3 (18.0, 253.8) | 394.2 (265.3, 877.3) | <0.001 |
| Sodium, mg | 43.8 (9.8, 114.2) | 46.4 (10.0, 114.0) | 34.2 (8.1, 126.3) | 0.61 |
| Grains (N) | 437 | 416 | 21 |  |
| Calcium, mg | 28.2 (10.1, 70.7) | 28.2 (9.6, 70.8) | 27.5 (16.6, 64.2) | 0.23 |
| Phosphorus, mg | 48.5 (36.2, 78.4) | 48.0 (36.1, 75.3) | 98.2 (80.2, 106.4) | <0.001 |
| Magnesium, mg | 13.9 (7.9, 23.8) | 13.5 (7.8, 22.0) | 33.7 (27.6, 37.5) | <0.001 |
| Iron, mg | 1.1 (0.8, 3.9) | 1.2 (0.8, 3.9) | 1.1 (0.9, 1.3) | 0.60 |
| Zinc, mg | 0.4 (0.3, 0.8) | 0.4 (0.3, 0.8) | 0.8 (0.6, 1.0) | 0.16 |
| Copper, mg | 0.1 (0.0, 0.1) | 0.1 (0.0, 0.1) | 0.1 (0.1, 0.1) | <0.001 |
| Selenium, mcg | 5.1 (3.5, 9.4) | 5.0 (3.4, 9.1) | 7.5 (5.4, 11.1) | 0.14 |
| Potassium, mg | 59.1 (40.1, 90.0) | 55.6 (39.4, 84.4) | 91.3 (86.9, 108.5) | 0.01 |
| Sodium, mg | 151.1 (115.9, 181.5) | 153.3 (119.9, 183.4) | 109.4 (66.2, 144.0) | <0.001 |
| Vegetables (N) | 525 | 212 | 313 |  |
| Calcium, mg | 41.5 (15.7, 90.0) | 19.9 (7.8, 45.4) | 57.1 (29.4, 121.6) | <0.001 |
| Phosphorus, mg | 66.3 (44.7, 100.9) | 50.4 (35.9, 69.1) | 85.4 (57.5, 121.9) | <0.001 |
| Magnesium, mg | 26.5 (17.0, 47.5) | 15.9 (11.2, 22.2) | 35.1 (23.4, 60.0) | <0.001 |
| Iron, mg | 0.9 (0.4, 1.9) | 0.4 (0.3, 0.8) | 1.5 (0.9, 2.3) | <0.001 |
| Zinc, mg | 0.5 (0.3, 0.9) | 0.3 (0.2, 0.5) | 0.6 (0.4, 1.0) | <0.001 |
| Copper, mg | 0.1 (0.1 0.2) | 0.1 (0.0, 0.1) | 0.1 (0.1, 0.2) | <0.001 |
| Selenium, mcg | 1.2 (0.5, 2.4) | 0.8 (0.2, 2.5) | 1.3 (0.7, 2.4) | <0.001 |
| Potassium, mg | 349.0 (221.2, 575.9) | 235.2 (154.7, 370.9) | 446.0 (303.1, 742.1) | <0.001 |
| Sodium, mg | 205.6 (140.2, 308.7) | 178.4 (145.5, 252.5) | 241.8 (135.7, 326.7) | 0.14 |
| Fruits (N) | 110 | 43 | 67 |  |
| Calcium, mg | 15.5 (9.5, 27.8) | 11.4 (6.4, 20.4) | 19.4 (12.0, 32.5) | <0.001 |
| Phosphorus, mg | 24.1 (16.0, 36.7) | 17.0 (11.4, 22.2) | 32.8 (22.5, 47.3) | <0.001 |
| Magnesium, mg | 17.2 (9.8, 25.0) | 10.0 (6.3, 18.5) | 20.0 (13.3, 27.6) | <0.001 |
| Iron, mg | 0.5 (0.3, 0.8) | 0.3 (0.2, 0.6) | 0.5 (0.3, 0.8) | <0.001 |
| Zinc, mg | 0.2 (0.1, 0.3) | 0.1 (0.1, 0.2) | 0.2 (0.1, 0.3) | 0.01 |
| Copper, mg | 0.1 (0.1, 0.2) | 0.1 (0.1, 0.1) | 0.1 (0.1, 0.2) | <0.001 |
| Selenium, mcg | 0.5 (0.2, 0.9) | 0.5 (0.2, 0.8) | 0.5 (0.2, 1.1) | 0.53 |
| Potassium, mg | 241.7 (147.1, 337.1) | 149.7 (87.5, 233.3) | 290.5 (218.0, 360.4) | <0.001 |
| Sodium, mg | 3.6 (1.9, 9.8) | 5.6 (2.6, 13.5) | 2.9 (1.7, 7.7) | 0.14 |
| Legumes (N) | 80 | 45 | 35 |  |
| Calcium, mg | 25.4 (17.7, 37.8) | 31.7 (21.7, 40.0) | 21.3 (14.8, 29.5) | 0.01 |
| Phosphorus, mg | 84.7 (71.8, 103.5) | 88.2 (72.7, 105.1) | 78.1 (71.8, 101.2) | 0.44 |
| Magnesium, mg | 28.5 (23.7, 36.1) | 28.3 (21.9, 36.1) | 29.4 (24.1, 36.1) | 0.49 |
| Iron, mg | 1.4 (1.1, 1.6) | 1.3 (1.1, 1.6) | 1.4 (1.1, 1.8) | 0.26 |
| Zinc, mg | 0.6 (0.5, 0.8) | 0.6 (0.5, 0.8) | 0.7 (0.5, 0.7) | 0.25 |
| Copper, mg | 0.1 (0.1, 0.2) | 0.1 (0.1, 0.2) | 0.1 (0.1, 0.2) | 0.29 |
| Selenium, mcg | 2.4 (1.0, 3.7) | 3.1 (1.6, 4.5) | 1.5 (0.7, 2.6) | 0.02 |
| Potassium, mg | 212.3 (163.4, 278.6) | 212.4 (164.4, 251.8) | 212.0 (159.4, 287.8) | 0.18 |
| Sodium, mg | 158.8 (116.8, 225.5) | 197.0 (159.0, 317.4) | 123.9 (111.2, 140.6) | <0.001 |
| Nuts, Seeds (N) | 77 | 24 | 53 |  |
| Calcium, mg | 12.0 (9.2, 18.0) | 9.4 (7.0, 12.7) | 14.7 (9.9, 22.0) | 0.01 |
| Phosphorus, mg | 66.3 (56.0, 82.7) | 56.6 (47.3, 66.1) | 75.3 (64.3, 85.4) | 0.04 |
| Magnesium, mg | 30.3 (22.2, 43.3) | 26.6 (21.0, 32.4) | 32.6 (24.2, 44.6) | 0.06 |
| Iron, mg | 0.6 (0.4, 0.7) | 0.4 (0.3, 0.6) | 0.6 (0.4, 0.8) | 0.08 |
| Zinc, mg | 0.5 (0.5, 0.7) | 0.5 (0.4, 0.6) | 0.5 (0.5, 0.8) | 0.09 |
| Copper, mg | 0.2 (0.1, 0.2) | 0.1 (0.1, 0.2) | 0.2 (0.2, 0.3) | <0.01 |
| Selenium, mcg | 1.6 (0.6, 5.8) | 1.4 (0.6, 3.2) | 1.7 (0.6, 8.0) | 0.33 |
| Potassium, mg | 101.5 (88.7, 121.8) | 93.8 (72.5, 111.6) | 108.0 (98.4, 125.8) | 0.02 |
| Sodium, mg | 29.6 (1.2, 61.6) | 65.0 (50.8, 81.9) | 3.2 (1.0, 42.0) | <0.001 |
| Meat (N) | 472 | 458 | 14 |  |
| Calcium, mg | 7.0 (4.7, 10.7) | 6.9 (4.7, 10.6) | 25.1 (5.6, 39.2) | <0.001 |
| Phosphorus, mg | 97.9 (72.4, 128.1) | 97.7 (70.8, 127.3) | 136.8 (111.0, 138.5) | 0.30 |
| Magnesium, mg | 10.1 (6.8, 13.4) | 10.1 (6.8, 13.4) | 10.9 (8.2, 14.7) | 0.54 |
| Iron, mg | 0.5 (0.4, 0.7) | 0.5 (0.4, 0.7) | 1.2 (0.8, 2.4) | <0.0001 |
| Zinc, mg | 1.0 (0.6, 1.5) | 1.0 (0.6, 1.5) | 0.9 (0.7, 2.6) | 0.88 |
| Copper, mg | 0.0 (0.0, 0.0) | 0.0 (0.0, 0.0) | 0.1 (0.0, 0.1) | 0.02 |
| Selenium, mcg | 12.7 (9.1, 16.8) | 12.6 (8.9, 16.6) | 20.3 (9.3, 21.5) | <0.01 |
| Potassium, mg | 132.7 (96.5, 170.1) | 132.7 (96.6, 170.1) | 121.4 (91.3, 163.7) | 0.94 |
| Sodium, mg | 220.0 (173.6, 298.6) | 223.9 (175.0, 300.0) | 147.4 (99.3, 179.7) | <0.01 |
| Seafood (N) | 193 | 176 | 17 |  |
| Calcium, mg | 21.8 (10.7, 43.1) | 20.9 (10.7, 42.5) | 34.8 (22.4, 45.3) | 0.20 |
| Phosphorus, mg | 154.0 (117.3, 203.6) | 150.6 (112.8, 198.8) | 183.9 (152.2, 230.2) | 0.14 |
| Magnesium, mg | 19.3 (14.3, 25.7) | 18.9 (14.1, 25.6) | 22.0 (17.0, 27.3) | 0.33 |
| Iron, mg | 0.5 (0.3, 0.7) | 0.5 (0.3, 0.7) | 1.0 (0.5, 1.4) | 0.01 |
| Zinc, mg | 0.4 (0.3, 0.7) | 0.4 (0.3, 0.6) | 0.6 (0.5, 1.3) | 0.06 |
| Copper, mg | 0.0 (0.0, 0.1) | 0.0 (0.0, 0.1) | 0.1 (0.0, 0.2) | <0.01 |
| Selenium, mcg | 23.4 (14.5, 33.8) | 22.2 (14.1, 33.1,) | 27.0 (21.5, 41.5) | 0.04 |
| Potassium, mg | 194.3 (128.5, 252.6) | 193.2 (130.6, 245.8) | 195.6 (79.8, 284.4) | 0.03 |
| Sodium, mg | 243.3 (181.6, 320.7) | 245.7 (186.3, 333.2) | 184.6 (65.1, 275.6) | <0.001 |
| Dairy (N) | 196 | 174 | 22 |  |
| Calcium, mg | 183.5 (112.2, 235.5) | 176.6 (103.1, 213.3) | 293.0 (285.7, 356.3) | <0.01 |
| Phosphorus, mg | 146.5 (108.8, 191.8) | 140.2 (105.4, 168.7) | 239.5 (223.0, 268.8) | <0.001 |
| Magnesium, mg | 13.7 (7.8, 21.9) | 11.2 (7.5, 20.2) | 27.9 (23.1, 34.4) | <0.001 |
| Iron, mg | 0.1 (0.0, 0.2) | 0.1 (0.0, 0.2) | 0.1 (0.1, 0.1) | 0.70 |
| Zinc, mg | 0.7 (0.5, 1.0) | 0.7 (0.5, 0.9) | 1.2 (1.0, 1.4) | <0.001 |
| Copper, mg | 0.0 (0.0, 0.0) | 0.0 (0.0, 0.0) | 0.0 (0.0, 0.0) | 0.82 |
| Selenium, mcg | 3.9 (2.5, 5.9) | 3.6 (2.4, 5.3) | 5.9 (5.2, 7.5) | <0.001 |
| Potassium, mg | 164.7 (49.9, 265.9) | 142.9 (44.6, 247.7) | 371.4 (305.8, 474.3) | <0.001 |
| Sodium, mg | 93.2 (57.7, 175.7) | 86.8 (56.3, 207.1) | 111.1 (90.7, 137.5) | 0.94 |
| Fats and Oils (N) | 68 | 60 | 8 |  |
| Calcium, mg | 3.7 (1.6, 9.1) | 3.9 (2.7, 9.6) | 0.0 (0.0, 0.0) | - |
| Phosphorus, mg | 4.6 (2.4, 9.4) | 5.8 (3.1, 11.8) | 0.0 (0.0, 0.0) | - |
| Magnesium, mg | 0.6 (0.2, 2.2) | 1.0 (0.3, 2.8) | 0.0 (0.0, 0.0) | - |
| Iron, mg | 0.1 (0.0, 0.2) | 0.1 (0.0, 0.2) | 0.0 (0.0, 0.0) | - |
| Zinc, mg | 0.0 (0.0, 0.1) | 0.0 (0.0, 0.1) | 0.0 (0.0, 0.0) | - |
| Copper, mg | 0.0 (0.0, 0.0) | 0.0 (0.0, 0.0) | 0.0 (0.0, 0.0) | - |
| Selenium, mcg | 0.3 (0.0, 1.0) | 0.4 (0.0, 1.1) | 0.0 (0.0, 0.0) | - |
| Potassium, mg | 11.0 (4.1, 27.4) | 13.9 (5.6, 33.6) | 0.0 (0.0, 0.0) | - |
| Sodium, mg | 186.2 (89.7, 362.5) | 212.3 (106.2, 451.7) | 0.0 (0.0, 0.0) | - |
| Mixed Dishes (N) | 1510 | 1441 | 69 |  |
| Calcium, mg | 32.4 (15.7, 63.5) | 33.0 (16.1, 64.4) | 17.1 (9.3, 37.9) | <0.0001 |
| Phosphorus, mg | 73.9 (54.3, 94.0) | 73.3 (53.8, 94.0) | 75.9 (67.1, 95.7) | 0.59 |
| Magnesium, mg | 11.2 (8.4, 16.2) | 11.1 (8.3, 15.3) | 27.1 (20.7, 31.2) | <0.0001 |
| Iron, mg | 0.8 (0.6, 0.9) | 0.8 (0.6, 0.9) | 0.6 (0.5, 1.0) | 0.01 |
| Zinc, mg | 0.5 (0.4, 0.8) | 0.5 (0.4, 0.8) | 0.5 (0.5, 0.7) | <0.01 |
| Copper, mg | 0.0 (0.0, 0.1) | 0.0 (0.0, 0.1) | 0.1 (0.1, 0.1) | <0.0001 |
| Selenium, mcg | 7.4 (5.0, 10.8) | 7.4 (5.2, 10.6) | 5.2 (3.9, 15.6) | 0.12 |
| Potassium, mg | 100.0 (71.8, 157.3) | 98.0 (70.4, 151.7) | 160.2 (112.5, 222.2) | <0.0001 |
| Sodium, mg | 225.4 (172.9, 288.9) | 227.5 (175.6, 291.0) | 161.9 (137.0, 243.2) | <0.0001 |
| Sauces and Condiments (N) | 178 | 169 | 9 |  |
| Calcium, mg | 22.7 (8.0, 57.3) | 21.7 (7.7, 56.1) | 56.0 (38.2, 58.8) | 0.06 |
| Phosphorus, mg | 32.7 (7.2, 73.3) | 28.2 (5.6, 71.5) | 56.0 (47.6, 100.0) | 0.07 |
| Magnesium, mg | 9.3 (2.3, 26.7) | 7.7 (2.3, 23.8) | 32.0 (27.3, 33.3) | 0.02 |
| Iron, mg | 0.4 (0.1, 1.3) | 0.3 (0.1, 1.3) | 0.4 (0.4, 1.1) | 0.28 |
| Zinc, mg | 0.2 (0.1, 0.5) | 0.2 (0.1, 0.5) | 0.3 (0.3, 0.7) | 0.08 |
| Copper, mg | 0.1 (0.0, 0.1) | 0.0 (0.0, 0.1) | 0.1 (0.1, 0.2) | 0.03 |
| Selenium, mcg | 0.8 (0.4, 2.3) | 0.8 (0.3, 2.4) | 0.5 (0.5, 1.2) | 0.77 |
| Potassium, mg | 87.5 (24.3, 364.5) | 79.3 (22.8, 328.4) | 468.0 (357.1, 468.2) | 0.01 |
| Sodium, mg | 301.9 (55.9, 1117.0) | 330.7 (75.6, 1194.3) | 11.4 (8.0, 76.2) | <0.001 |
| Snacks and Desserts (N) | 680 | 671 | 9 |  |
| Calcium, mg | 12.0 (5.3, 27.4) | 12.0 (5.4, 27.4) | 8.5 (2.8, 52.5) | 0.39 |
| Phosphorus, mg | 31.6 (21.3, 47.9) | 31.3 (21.1, 47.6) | 82.6 (73.5, 91.8) | <0.001 |
| Magnesium, mg | 7.6 (4.4, 12.3) | 7.5 (4.4, 12.1) | 36.2 (33.4, 36.2) | <0.001 |
| Iron, mg | 0.4 (0.2, 0.6) | 0.4 (0.2, 0.6) | 0.6 (0.4, 0.6) | 0.29 |
| Zinc, mg | 0.2 (0.1, 0.3) | 0.2 (0.1, 0.3) | 0.7 (0.6, 0.8) | <0.001 |
| Copper, mg | 0.0 (0.0, 0.1) | 0.0 (0.0, 0.1) | 0.1 (0.1, 0.1) | <0.001 |
| Selenium, mcg | 1.6 (0.8, 3.0) | 1.5 (0.8, 3.0) | 3.8 (2.6, 6.3) | 0.02 |
| Potassium, mg | 47.2 (28.4, 71.5) | 46.4 (28.2, 71.2) | 85.2 (84.1, 87.2) | 0.01 |
| Sodium, mg | 76.9 (44.9, 112.0) | 76.7 (44.7, 114.3) | 84.9 (66.5, 96.7) | 0.80 |

1. Abbreviations: milligrams (mg), microgram (mcg), interquartile range (IQR)
2. P-values from t-tests comparing mean log-transformed nutrient values between FDA-aligned and FDA-unaligned groups

**Supplemental Table 15.** Mean Macronutrients per RACC of FDA-Aligned and FDA-Unaligned Foods and Beverages Consumed in the United States NHANES/FNDDS 2017-2018, Overall and Stratified by Nova^1^

|  | **Overall**  **Median (IQR)** | **FDA Unaligned**  **Median (IQR)** | **FDA Aligned**  **Median (IQR)** | **p-value^2^** |
| --- | --- | --- | --- | --- |
| Overall (N) |  |  |  |  |
| Protein, g | 5.5 (1.9, 14.8) | 6.8 (2.4, 16.3) | 1.8 (0.8, 5.0) | <0.001 |
| Carbohydrate, g | 16.7 (4.9, 31.8) | 19.2 (6.1, 33.4) | 6.8 (3.2, 17.4) | <0.001 |
| Sugars, added, g | 0.0 (0.0, 3.6) | 0.2 (0.0, 5.4) | 0.0 (0.0, 0.0) | <0.001 |
| Fiber, total dietary, g | 1.2 (0.2, 2.4) | 1.0 (0.1, 2.3) | 1.8 (0.6, 2.8) | <0.001 |
| Saturated fat, g | 1.6 (0.4, 3.7) | 2.0 (0.6, 4.3) | 0.4 (0.0, 0.9) | <0.001 |
| Monounsaturated fat, g | 2.0 (0.5, 4.6) | 2.4 (0.8, 5.1) | 0.5 (0.0, 1.1) | <0.001 |
| Polyunsaturated fat, g | 1.1 (0.3, 2.9) | 1.3 (0.4, 3.1) | 0.4 (0.1, 1.1) | <0.001 |
| Minimally Processed Foods (N) |  |  |  |  |
| Protein, g | 8.1 (2.7, 18.0) | 12.9 (5.3, 21.0) | 1.7 (0.8, 4.3) | <0.001 |
| Carbohydrate, g | 12.0 (2.9, 28.3) | 14.9 (2.6, 31.4) | 6.7 (3.2, 17.4) | <0.001 |
| Sugars, added, g | 0.0 (0.0, 0.0) | 0.0 (0.0, 0.5) | 0.0 (0.0, 0.0) | <0.01 |
| Fiber, total dietary, g | 1.2 (0.2, 2.4) | 1.0 (0.0, 2.2) | 1.8 (0.6, 2.8) | <0.01 |
| Saturated fat, g | 1.6 (0.5, 3.2) | 2.4 (1.2, 4.1) | 0.4 (0.0, 0.8) | <0.001 |
| Monounsaturated fat, g | 2.0 (0.7, 4.4) | 3.0 (1.3, 5.4) | 0.5 (0.0, 1.0) | <0.001 |
| Polyunsaturated fat, g | 1.2 (0.4, 2.8) | 1.7 (0.6, 3.5) | 0.3 (0.1, 1.0) | <0.001 |
| Processed Culinary Ingredients (N) |  |  |  |  |
| Protein, g | 0.1 (0.0, 1.1) | 0.3 (0.0, 1.1) | 0.0 (0.0, 0.0) | - |
| Carbohydrate, g | 1.5 (0.0, 10.4) | 2.9 (0.6, 14.9) | 0.0 (0.0, 0.0) | - |
| Sugars, added, g | 0.0 (0.0, 8.0) | 0.0 (0.0, 12.0) | 0.0 (0.0, 0.0) | - |
| Fiber, total dietary, g | 0.0 (0.0, 0.0) | 0.0 (0.0, 0.0) | 0.0 (0.0, 0.0) | - |
| Saturated fat, g | 1.4 (0.0, 4.7) | 1.4 (0.0, 7.2) | 1.4 (1.1, 1.9) | 0.52 |
| Monounsaturated fat, g | 0.9 (0.0, 3.5) | 0.7 (0.0, 2.8) | 5.6 (3.2, 8.9) | 0.18 |
| Polyunsaturated fat, g | 0.2 (0.0, 0.9) | 0.1 (0.0, 0.5) | 5.8 (2.4, 7.7) | 0.01 |
| Processed Foods (N) |  |  |  |  |
| Protein, g | 4.5 (0.9, 8.3) | 4.7 (0.9, 9.2) | 3.9 (1.9, 6.6) | 0.97 |
| Carbohydrate, g | 5.4 (0.7, 13.6) | 3.8 (0.6, 14.3) | 6.1 (4.4, 10.5) | 0.12 |
| Sugars, added, g | 0.0 (0.0, 0.5) | 0.0 (0.0, 1.3) | 0.0 (0.0, 0.0) | 0.01 |
| Fiber, total dietary, g | 0.5 (0.0, 2.0) | 0.2 (0.0, 1.6) | 2.3 (1.5, 2.9) | <0.001 |
| Saturated fat, g | 1.0 (0.1, 2.4) | 1.0 (0.0, 2.8) | 0.9 (0.6, 2.1) | 0.57 |
| Monounsaturated fat, g | 1.2 (0.1, 2.6) | 1.1 (0.0, 2.5) | 1.7 (0.4, 8.2) | 0.01 |
| Polyunsaturated fat, g | 0.3 (0.1, 1.6) | 0.3 (0.1, 1.3) | 1.1 (0.3, 3.9) | <0.001 |
| Ultra-Processed Foods (N) |  |  |  |  |
| Protein, g | 4.0 (1.6, 11.4) | 4.0 (1.6, 11.4) | 2.5 (1.2, 7.4) | 0.93 |
| Carbohydrate, g | 23.4 (12.6, 36.0) | 23.5 (12.6, 36.0) | 15.7 (3.0, 38.0) | 0.24 |
| Sugars, added, g | 2.7 (0.0, 9.8) | 2.8 (0.0, 10.0) | 0.0 (0.0, 1.6) | 0.01 |
| Fiber, total dietary, g | 1.1 (0.3, 2.4) | 1.1 (0.3, 2.4) | 1.8 (0.5, 3.2) | <0.01 |
| Saturated fat, g | 1.6 (0.3, 4.3) | 1.7 (0.4, 4.3) | 0.2 (0.1, 0.5) | <0.001 |
| Monounsaturated fat, g | 1.9 (0.4, 4.7) | 1.9 (0.5, 4.8) | 0.2 (0.0, 0.9) | <0.001 |
| Polyunsaturated fat, g | 1.1 (0.3, 2.9) | 1.1 (0.3, 3.0) | 0.4 (0.1, 1.2) | 0.04 |

1. Abbreviations: Reference amount customarily consumed (RACC), grams (g), interquartile range (IQR)
2. P-values from t-tests comparing mean log-transformed nutrient values between FDA-aligned and FDA-unaligned groups

**Supplemental Table 16.** Mean Macronutrients per RACC of FDA-Aligned and FDA-Unaligned Foods and Beverages Consumed in the United States NHANES/FNDDS 2017-2018, Overall and Stratified by Food Category^1^

|  | **Overall**  **Median (IQR)** | **FDA Unaligned**  **Median (IQR)** | **FDA Aligned**  **Median (IQR)** | **p-value^2^** |
| --- | --- | --- | --- | --- |
| Beverages (N) | 422 | 324 | 98 |  |
| Protein, g | 0.4 (0.0, 2.3) | 0.4 (0.0, 3.1) | 0.8 (0.2, 1.7) | 0.28 |
| Carbohydrate, g | 18.6 (3.4, 32.5) | 18.6 (6.0, 32.7) | 18.8 (0.6, 30.9) | 0.52 |
| Sugars, added, g | 0.9 (0.0, 19.0) | 12.3 (0.0, 23.7) | 0.0 (0.0, 0.0) | <0.001 |
| Fiber, total dietary, g | 0.0 (0.0, 0.5) | 0.0 (0.0, 0.4) | 0.5 (0.0, 1.5) | 0.02 |
| Saturated fat, g | 0.0 (0.0, 0.2) | 0.0 (0.0, 0.4) | 0.0 (0.0, 0.1) | <0.001 |
| Monounsaturated fat, g | 0.0 (0.0, 0.2) | 0.0 (0.0,0.7) | 0.0 (0.0, 0.1) | <0.001 |
| Polyunsaturated fat, g | 0.0 (0.0, 0.2) | 0.0 (0.0, 0.2) | 0.1 (0.0, 0.1) | 0.04 |
| Grains (N) | 437 | 416 | 21 |  |
| Protein, g | 3.9 (2.7, 5.6) | 3.9 (2.7, 5.6) | 5.0 (4.0, 5.4) | 0.87 |
| Carbohydrate, g | 29.8 (22.1, 37.0) | 30.0 (22.1, 36.8) | 27.3 (15.7, 39.3) | 0.23 |
| Sugars, added, g | 1.7 (0.0, 9.0) | 1.9 (0.1, 9.3) | 0.0 (0.0, 1.6) | 0.18 |
| Fiber, total dietary, g | 2.0 (1.1, 3.8) | 1.9 (1.1, 3.6) | 4.1 (2.6, 4.8) | <0.01 |
| Saturated fat, g | 0.4 (0.2, 1.4) | 0.4 (0.2, 1.5) | 0.3 (0.2, 0.4) | 0.02 |
| Monounsaturated fat, g | 0.6 (0.2, 1.5) | 0.6 (0.2, 1.5) | 0.3 (0.2, 0.8) | 0.08 |
| Polyunsaturated fat, g | 0.6 (0.3, 1.1) | 0.6 (0.3, 1.1) | 0.7 (0.5, 0.9) | 0.54 |
| Vegetables (N) | 525 | 212 | 313 |  |
| Protein, g | 1.9 (1.1, 2.9) | 2.7 (1.9, 4.1) | 1.3 (0.8, 2.3) | <0.001 |
| Carbohydrate, g | 7.9 (3.7, 17.7) | 17.4 (10.6, 29.6) | 4.8 (3.1, 9.9) | <0.001 |
| Sugars, added, g | 0.0 (0.0, 0.0) | 0.0 (0.0, 0.0) | 0.0 (0.0, 0.0) | 0.16 |
| Fiber, total dietary, g | 2.0 (1.3, 2.7) | 2.1 (1.4, 3.1) | 1.9 (1.2, 2.6) | <0.001 |
| Saturated fat, g | 0.7 (0.1, 1.2) | 1.5 (0.9, 2.8) | 0.4 (0.0, 0.7) | <0.001 |
| Monounsaturated fat, g | 0.8 (0.0, 1.7) | 2.3 (0.9, 4.0) | 0.6 (0.0, 0.9) | <0.001 |
| Polyunsaturated fat, g | 0.7 (0.2, 1.3) | 1.4 (0.6, 3.4) | 0.5 (0.1, 0.8) | <0.001 |
| Fruits (N) | 110 | 43 | 67 |  |
| Protein, g | 0.7 (0.5, 1.0) | 0.7 (0.5, 1.0) | 0.7 (0.5, 1.0) | 0.32 |
| Carbohydrate, g | 14.9 (9.9, 25.6) | 17.6 (14.6, 28.3) | 12.0 (8.4, 25.6) | <0.01 |
| Sugars, added, g | 0.0 (0.0, 2.9) | 4.7 (1.5, 9.5) | 0.0 (0.0, 0.0) | 0.12 |
| Fiber, total dietary, g | 1.6 (1.1, 2.5) | 1.5 (1.0, 1.9) | 1.8 (1.3, 3.2) | 0.13 |
| Saturated fat, g | 0.0 (0.0, 0.1) | 0.0 (0.0, 0.7) | 0.0 (0.0, 0.0) | 0.03 |
| Monounsaturated fat, g | 0.0 (0.0, 0.1) | 0.0 (0.0, 0.2) | 0.0 (0.0, 0.1) | 0.05 |
| Polyunsaturated fat, g | 0.1 (0.0, 0.1) | 0.0 (0.0, 0.2) | 0.1 (0.0, 0.1) | 0.52 |
| Legumes (N) | 80 | 45 | 35 |  |
| Protein, g | 7.7 (6.7, 8.7) | 7.9 (6.8, 10.4) | 7.7 (6.7, 7.9) | 0.30 |
| Carbohydrate, g | 21.6 (17.6, 23.6) | 22.2 (15.3, 26.7) | 21.2 (17.7, 22.4) | 0.10 |
| Sugars, added, g | 0.0 (0.0, 0.0) | 0.0 (0.0, 0.0) | 0.0 (0.0, 0.0) | - |
| Fiber, total dietary, g | 6.6 (4.8, 7.7) | 6.6 (4.0, 7.9) | 6.5 (5.5, 7.6) | 0.06 |
| Saturated fat, g | 0.9 (0.3, 1.0) | 0.9 (0.3, 1.5) | 0.9 (0.2, 0.9) | 0.08 |
| Monounsaturated fat, g | 2.5 (0.5, 2.6) | 1.7 (0.5, 3.0) | 2.5 (0.5, 2.6) | 0.74 |
| Polyunsaturated fat, g | 2.6 (0.7, 2.7) | 2.3 (0.7, 3.3) | 2.6 (0.7, 2.7) | 0.70 |
| Nuts and Seeds (N) | 77 | 24 | 53 |  |
| Protein, g | 5.4 (3.8, 5.9) | 3.9 (3.1, 6.5) | 5.5 (4.2, 5.9) | 0.12 |
| Carbohydrate, g | 6.0 (4.7, 8.4) | 8.5 (6.3, 11.8) | 5.9 (4.3, 6.5) | <0.001 |
| Sugars, added, g | 0.0 (0.0, 0.8) | 1.9 (1.0, 6.1) | 0.0 (0.0, 0.0) | <0.01 |
| Fiber, total dietary, g | 2.2 (1.8, 2.7) | 1.8 (1.4, 2.3) | 2.4 (2.1, 2.8) | 0.01 |
| Saturated fat, g | 2.0 (1.5, 2.4) | 1.9 (1.4, 3.2) | 2.0 (1.6, 2.2) | 0.33 |
| Monounsaturated fat, g | 7.3 (4.4, 8.3) | 5.3 (3.0, 8.0) | 7.6 (6.7, 8.6) | 0.05 |
| Polyunsaturated fat, g | 3.8 (2.6, 5.2) | 3.5 (2.4, 4.5) | 3.9 (2.7, 5.6) | 0.07 |
| Meat (N) | 472 | 458 | 14 |  |
| Protein, g | 18.9 (11.2, 23.5) | 18.9 (11.8, 23.5) | 6.3 (3.6, 24.1) | <0.001 |
| Carbohydrate, g | 0.1 (0.0, 2.9) | 0.1 (0.0, 3.0) | 0.3 (0.0, 0.5) | 0.03 |
| Sugars, added, g | 0.0 (0.0, 0.0) | 0.0 (0.0, 0.0) | 0.0 (0.0, 0.0) | - |
| Fiber, total dietary, g | 0.0 (0.0, 0.0) | 0.0 (0.0, 0.0) | 0.0 (0.0, 0.0) | - |
| Saturated fat, g | 2.6 (1.5, 4.2) | 2.7 (1.5, 4.3) | 1.6 (0.8, 1.7) | 0.02 |
| Monounsaturated fat, g | 3.6 (2.1, 5.8) | 3.8 (2.2, 6.0) | 1.3 (0.8, 2.0) | <0.0001 |
| Polyunsaturated fat, g | 1.3 (0.6, 2.5) | 1.4 (0.6, 2.5) | 0.5 (0.2, 1.0) | <0.01 |
| Seafood (N) | 193 | 176 | 17 |  |
| Protein, g | 21.1 (11.6, 25.5) | 21.7 (15.3, 26.0) | 10.0 (9.5, 11.7) | <0.001 |
| Carbohydrate, g | 0.6 (0.1, 11.1) | 0.2 (0.1, 11.1) | 1.8 (0.1, 2.5) | 0.97 |
| Sugars, added, g | 0.0 (0.0, 0.5) | 0.0 (0.0, 0.5) | 0.0 (0.0, 0.0) | 0.03 |
| Fiber, total dietary, g | 0.0 (0.0, 0.7) | 0.0 (0.0, 0.7) | 0.0 (0.0, 0.0) | <0.001 |
| Saturated fat, g | 1.2 (0.5, 2.1) | 1.3 (0.7, 2.4) | 0.4 (0.2, 0.5) | <0.001 |
| Monounsaturated fat, g | 2.4 (0.7, 3.7) | 2.5 (0.8, 3.8) | 0.8 (0.1, 1.0) | <0.001 |
| Polyunsaturated fat, g | 1.6 (0.6, 3.2) | 1.9 (0.7, 3.3) | 0.5 (0.2, 1.0) | <0.001 |
| Dairy (N) | 196 | 174 | 22 |  |
| Protein, g | 7.0 (3.4, 8.5) | 5.8 (3.0, 8.4) | 8.6 (8.2, 9.0) | <0.01 |
| Carbohydrate, g | 7.0 (1.6, 18.4) | 5.8 (1.4, 20.1) | 11.9 (5.9, 12.7) | 0.06 |
| Sugars, added, g | 0.0 (0.0, 7.7) | 0.0 (0.0, 8.3) | 0.0 (0.0, 0.0) | - |
| Fiber, total dietary, g | 0.0 (0.0, 0.0) | 0.0 (0.0, 0.0) | 0.0 (0.0, 0.0) | - |
| Saturated fat, g | 2.1 (0.8, 3.7) | 2.4 (0.9, 3.8) | 1.4 (0.1, 1.7) | <0.001 |
| Monounsaturated fat, g | 0.9 (0.4, 1.5) | 1.0 (0.5, 1.6) | 0.5 (0.0, 0.7) | <0.001 |
| Polyunsaturated fat, g | 0.1 (0.1, 0.2) | 0.1 (0.1, 0.2) | 0.1 (0.0, 0.1) | <0.001 |
| Fats and Oils (N) | 68 | 60 | 8 |  |
| Protein, g | 0.1 (0.0, 0.3) | 0.1 (0.1, 0.4) | 0.0 (0.0, 0.0) | - |
| Carbohydrate, g | 1.2 (0.0, 3.7) | 1.7 (0.1, 5.1) | 0.0 (0.0, 0.0) | - |
| Sugars, added, g | 0.6 (0.0, 2.4) | 0.9 (0.0, 2.6) | 0.0 (0.0, 0.0) | - |
| Fiber, total dietary, g | 0.0 (0.0, 0.0) | 0.0 (0.0, 0.1) | 0.0 (0.0, 0.0) | - |
| Saturated fat, g | 1.6 (0.4, 2.1) | 1.6 (0.3, 2.6) | 1.6 (1.2, 1.9) | 0.47 |
| Monounsaturated fat, g | 2.3 (0.9, 3.0) | 1.9 (0.6, 2.8) | 5.7 (3.5, 9.3) | <0.01 |
| Polyunsaturated fat, g | 1.9 (0.5, 4.3) | 1.3 (0.5, 3.7) | 5.8 (3.2, 8.3) | 0.01 |
| Mixed Dishes (N) | 1510 | 1441 | 69 |  |
| Protein, g | 14.9 (9.2, 21.1) | 15.3 (9.6, 21.5) | 7.1 (3.9, 10.4) | <0.0001 |
| Carbohydrate, g | 29.1 (14.9, 40.0) | 29.2 (15.5, 40.4) | 22.6 (7.3, 32.1) | <0.001 |
| Sugars, added, g | 0.0 (0.0, 2.4) | 0.0 (0.0, 2.6) | 0.0 (0.0, 0.0) | 0.14 |
| Fiber, total dietary, g | 2.1 (1.1, 3.3) | 2.1 (1.1, 3.3) | 2.5 (1.5, 3.2) | <0.01 |
| Saturated fat, g | 3.6 (1.7, 7.5) | 3.7 (1.9, 7.7) | 0.8 (0.6, 1.3) | <0.0001 |
| Monounsaturated fat, g | 4.5 (2.4, 7.0) | 4.7 (2.5, 7.2) | 1.5 (1.0, 3.1) | <0.0001 |
| Polyunsaturated fat, g | 2.6 (1.3, 4.3) | 2.6 (1.3, 4.4) | 1.5 (0.8, 2.4) | <0.0001 |
| Sauce and Condiments (N) | 178 | 169 | 9 |  |
| Protein, g | 0.3 (0.1, 1.3) | 0.3 (0.1, 1.3) | 0.0 (0.0, 0.2) | <0.01 |
| Carbohydrate, g | 3.2 (1.4, 7.9) | 3.3 (1.7, 8.0) | 0.7 (0.6, 1.1) | 0.01 |
| Sugars, added, g | 0.0 (0.0, 4.9) | 0.1 (0.0, 5.2) | 0.0 (0.0, 0.0) | - |
| Fiber, total dietary, g | 0.2 (0.0, 0.5) | 0.2 (0.0, 0.5) | 0.0 (0.0, 0.1) | 0.01 |
| Saturated fat, g | 0.0 (0.0, 1.1) | 0.0 (0.0, 1.1) | 0.0 (0.0, 0.0) | <0.001 |
| Monounsaturated fat, g | 0.0 (0.0, 1.6) | 0.0 (0.0, 1.7) | 0.0 (0.0, 0.0) | <0.001 |
| Polyunsaturated fat, g | 0.0 (0.0, 0.4) | 0.0 (0.0, 0.4) | 0.0 (0.0, 0.0) | <0.001 |
| Snack and Desserts (N) | 680 | 671 | 9 |  |
| Protein, g | 2.5 (1.6, 3.7) | 2.5 (1.6, 3.7) | 1.7 (1.4, 2.0) | 0.27 |
| Carbohydrate, g | 22.4 (14.0, 37.8) | 22.5 (14.0, 38.0) | 14.6 (11.9, 15.5) | 0.02 |
| Sugars, added, g | 9.4 (1.1, 16.1) | 9.5 (1.4, 16.2) | 0.0 (0.0, 0.0) | - |
| Fiber, total dietary, g | 1.0 (0.5, 1.7) | 1.0 (0.5, 1.7) | 1.8 (1.2, 2.3) | 0.15 |
| Saturated fat, g | 2.0 (0.8, 3.8) | 2.0 (0.8, 3.9) | 0.2 (0.1, 0.4) | <0.001 |
| Monounsaturated fat, g | 2.1 (1.0, 4.1) | 2.1 (1.0, 4.1) | 0.3 (0.2, 1.3) | <0.01 |
| Polyunsaturated fat, g | 1.2 (0.5, 2.7) | 1.2 (0.5, 2.7) | 0.5 (0.3, 1.1) | 0.13 |

1. Abbreviations: Reference amount customarily consumed (RACC), grams (g), interquartile range (IQR)
2. P-values from t-tests comparing mean log-transformed nutrient values between FDA-aligned and FDA-unaligned groups

**Supplemental Table 17.** Mean Vitamins per RACC of FDA-Aligned and FDA-Unaligned Foods and Beverages Consumed in the United States NHANES/FNDDS 2017-2018, Overall and Stratified by Nova ^1^

|  | **Overall**  **Median (IQR)** | **FDA Unaligned**  **Median (IQR)** | **FDA Aligned**  **Median (IQR)** | **p-value^2^** |
| --- | --- | --- | --- | --- |
| Overall (N) | 4949 | 4214 | 735 |  |
| Vitamin A, mcg_RAE, | 17.6 (0.8, 86.4) | 19.5 (1.0, 93.7) | 11.1 (0.2, 52.3) | <0.01 |
| Thiamin, mg | 0.1 (0.0, 0.2) | 0.1 (0.0, 0.3) | 0.0 (0.0, 0.1) | <0.001 |
| Riboflavin, mg | 0.1 (0.0, 0.3) | 0.1 (0.1, 0.3) | 0.1 (0.0, 0.1) | <0.001 |
| Niacin, mg | 1.5 (0.4, 4.0) | 1.8 (0.6, 4.5) | 0.5 (0.3, 1.0) | <0.001 |
| Vitamin B-6, mg | 0.1 (0.0, 0.3) | 0.1 (0.0, 0.3) | 0.1 (0.0, 0.1) | <0.001 |
| Folate, mcg_DFE | 22.4 (6.0, 64.4) | 24.3 (5.5, 70.0) | 18.0 (7.2, 32.6) | <0.001 |
| Vitamin B-12, mcg | 0.1 (0.0, 0.7) | 0.2 (0.0, 0.8) | 0.0 (0.0, 0.0) | 0.14 |
| Vitamin C, mg | 0.6 (0.0, 5.9) | 0.3 (0.0, 4.2) | 3.5 (0.3, 16.8) | <0.001 |
| Vitamin D, mcg | 0.0 (0.0, 0.3) | 0.0 (0.0, 0.4) | 0.0 (0.0, 0.0) | 0.02 |
| Vitamin E, mg | 0.5 (0.2, 1.2) | 0.6 (0.2, 1.2) | 0.4 (0.1, 0.9) | <0.001 |
| Vitamin K, mcg | 3.3 (0.5, 11.3) | 3.2 (0.5, 10.4) | 4.6 (0.7, 18.1) | <0.001 |
| Choline, mg | 20.9 (6.3, 49.5) | 24.8 (6.4, 54.7) | 12.0 (5.5, 21.7) | <0.001 |
| Minimally Processed Foods (N) | 2257 | 1628 | 629 |  |
| Vitamin A, mcg_RAE, | 25.0 (4.3, 90.6) | 30.0 (6.7, 103.2) | 15.8 (0.9, 62.3) | <0.001 |
| Thiamin, mg | 0.1 (0.0, 0.2) | 0.1 (0.1, 0.2) | 0.0 (0.0, 0.1) | <0.001 |
| Riboflavin, mg | 0.1 (0.1, 0.3) | 0.2 (0.1, 0.3) | 0.1 (0.0, 0.1) | <0.001 |
| Niacin, mg | 1.6 (0.5, 4.1) | 2.6 (1.2, 5.1) | 0.5 (0.3, 0.9) | <0.001 |
| Vitamin B-6, mg | 0.2 (0.1, 0.3) | 0.2 (0.1, 0.4) | 0.1 (0.0, 0.1) | <0.001 |
| Folate, mcg_DFE | 22.4 (9.0, 52.0) | 26.0 (9.0, 63.3) | 18.0 (8.3, 37.4) | <0.001 |
| Vitamin B-12, mcg | 0.2 (0.0, 0.9) | 0.4 (0.1, 1.1) | 0.0 (0.0, 0.0) | 0.39 |
| Vitamin C, mg | 1.6 (0.0, 9.2) | 0.6 (0.0, 6.8) | 5.2 (1.0, 19.0) | <0.001 |
| Vitamin D, mcg | 0.0 (0.0, 0.4) | 0.2 (0.0, 0.7) | 0.0 (0.0, 0.0) | 0.18 |
| Vitamin E, mg | 0.6 (0.2, 1.2) | 0.7 (0.3, 1.3) | 0.4 (0.1, 0.8) | <0.001 |
| Vitamin K, mcg | 5.1 (1.0, 15.0) | 4.8 (1.0, 13.5) | 6.3 (1.0, 22.7) | <0.001 |
| Choline, mg | 34.5 (11.5, 67.2) | 47.8 (19.3, 77.6) | 11.9 (5.7, 21.9) | <0.001 |
| Processed Culinary Ingredients (N) | 52 | 43 | 9 |  |
| Vitamin A, mcg_RAE, | 0.0 (0.0, 38.3) | 1.6 (0.0, 47.2) | 0.0 (0.0, 0.0) | - |
| Thiamin, mg | 0.0 (0.0, 0.0) | 0.0 (0.0, 0.0) | 0.0 (0.0, 0.0) | - |
| Riboflavin, mg | 0.0 (0.0, 0.0) | 0.0 (0.0, 0.0) | 0.0 (0.0, 0.0) | - |
| Niacin, mg | 0.0 (0.0,0.0) | 0.0 (0.0, 0.1) | 0.0 (0.0, 0.0) | - |
| Vitamin B-6, mg | 0.0 (0.0, 0.0) | 0.0 (0.0, 0.0) | 0.0 (0.0, 0.0) | - |
| Folate, mcg_DFE | 0.4 (0.0, 1.5) | 0.4 (0.0, 1.8) | 0.0 (0.0, 0.0) | - |
| Vitamin B-12, mcg | 0.0 (0.0, 0.0) | 0.0 (0.0, 0.0) | 0.0 (0.0, 0.0) | - |
| Vitamin C, mg | 0.0 (0.0, 0.2) | 0.0 (0.0, 0.3) | 0.0 (0.0, 0.0) | - |
| Vitamin D, mcg | 0.0 (0.0, 0.0) | 0.0 (0.0, 0.0) | 0.0 (0.0, 0.0) | - |
| Vitamin E, mg | 0.1 (0.0, 0.4) | 0.1 (0.0, 0.3) | 2.0 (0.2, 2.4) | <0.001 |
| Vitamin K, mcg | 0.3 (0.0, 1.1) | 0.1 (0.0, 0.6) | 1.9 (0.8, 8.4) | <0.01 |
| Choline, mg | 2.4 (0.0, 5.1) | 2.7 (0.1, 5.8) | 0.0 (0.0, 0.1) | <0.001 |
| Processed Foods (N) | 325 | 277 | 48 |  |
| Vitamin A, mcg_RAE, | 4.3 (0.0, 36.5) | 5.4 (0.0, 38.4) | 1.3 (0.0, 17.9) | 0.14 |
| Thiamin, mg | 0.0 (0.0, 0.1) | 0.0 (0.0, 0.1) | 0.0 (0.0, 0.1) | 0.37 |
| Riboflavin, mg | 0.1 (0.0, 0.1) | 0.1 (0.0,0.1) | 0.1 (0.0, 0.1) | 0.57 |
| Niacin, mg | 0.5 (0.1, 1.7) | 0.5 (0.1, 1.8) | 0.8 (0.4, 1.0) | 0.07 |
| Vitamin B-6, mg | 0.1 (0.0, 0.1) | 0.1 (0.0, 0.2) | 0.1 (0.0, 0.1) | 0.26 |
| Folate, mcg_DFE | 7.0 (2.2, 21.6) | 5.7 (1.8, 18.6) | 20.0 (11.3, 28.9) | <0.001 |
| Vitamin B-12, mcg | 0.0 (0.0, 0.3) | 0.1 (0.0, 0.3) | 0.0 (0.0, 0.0) | 0.52 |
| Vitamin C, mg | 0.2 (0.0, 2.9) | 0.1 (0.0, 3.3) | 0.6 (0.0, 2.1) | 0.02 |
| Vitamin D, mcg | 0.0 (0.0, 0.1) | 0.0 (0.0, 0.1) | 0.0 (0.0, 0.0) | 0.86 |
| Vitamin E, mg | 0.2 (0.1, 0.9) | 0.2 (0.1, 0.8) | 0.6 (0.3, 1.9) | <0.001 |
| Vitamin K, mcg | 0.6 (0.1, 4.3) | 0.5 (0.1, 4.2) | 2.0 (0.1, 4.6) | 0.24 |
| Choline, mg | 14.6 (4.1, 30.6) | 13.3 (3.2, 34.2) | 14.9 (11.7, 18.0) | 0.25 |
| Ultra-Processed Foods (N) | 2169 | 2123 | 46 |  |
| Vitamin A, mcg_RAE, | 9.2 (0.0, 92.5) | 9.9 (0.0, 96.3) | 0.0 (0.0, 6.5) | 0.41 |
| Thiamin, mg | 0.1 (0.0, 0.3) | 0.1 (0.0, 0.3) | 0.1 (0.0, 0.2) | 0.86 |
| Riboflavin, mg | 0.1 (0.0, 0.3) | 0.1 (0.0, 0.3) | 0.1 (0.0, 0.2) | 0.62 |
| Niacin, mg | 1.5 (0.4, 4.2) | 1.5 (0.4, 4.3) | 1.0 (0.4, 2.9) | 0.49 |
| Vitamin B-6, mg | 0.1 (0.0, 0.2) | 0.1 (0.0, 0.2) | 0.1 (0.0, 0.2) | 0.05 |
| Folate, mcg_DFE | 27.0 (3.9, 81.9) | 27.3 (3.9, 83.4) | 15.4 (4.2, 37.2) | 0.78 |
| Vitamin B-12, mcg | 0.1 (0.0, 0.6) | 0.1 (0.0, 0.6) | 0.0 (0.0, 0.6) | 0.01 |
| Vitamin C, mg | 0.2 (0.0, 2.8) | 0.2 (0.0, 2.8) | 0.0 (0.0, 5.4) | 0.07 |
| Vitamin D, mcg | 0.0 (0.0, 0.3) | 0.0 (0.0, 0.3) | 0.0 (0.0, 0.0) | 0.29 |
| Vitamin E, mg | 0.4 (0.1, 1.2) | 0.5 (0.1, 1.2) | 0.2 (0.1, 1.7) | 0.51 |
| Vitamin K, mcg | 2.2 (0.4, 7.5) | 2.2 (0.4, 7.5) | 1.0 (0.2, 4.5) | 0.51 |
| Choline, mg | 12.8 (4.1, 35.7) | 13.1 (4.1, 35.9) | 7.9 (3.6, 24.8) | 0.90 |

1. Abbreviations: Reference amount customarily consumed (RACC), grams (g), milligrams (mg), microgram (mcg), interquartile range (IQR), retinol activity equivalent (RAE), dietary folate equivalent (DFE)
2. P-values from t-tests comparing mean log-transformed nutrient values between FDA-aligned and FDA-unaligned groups

**Supplemental Table 18.** Mean Vitamins per RACC of FDA-Aligned and FDA-Unaligned Foods and Beverages Consumed in the United States NHANES/FNDDS 2017-2018, Overall and Stratified by Food Category ^1^

|  | **Overall** | **FDA Unaligned** | **FDA Aligned** | **p-value^2^** |
| --- | --- | --- | --- | --- |
| Beverages (N) | 422 | 324 | 98 |  |
| Vitamin A, mcg_RAE, | 0.0 (0.0, 86.8) | 0.0 (0.0, 125.9) | 0.0 (0.0, 26.0) | <0.001 |
| Thiamin, mg | 0.0 (0.0, 0.1) | 0.0 (0.0, 0.1) | 0.0 (0.0, 0.1) | <0.01 |
| Riboflavin, mg | 0.0 (0.0, 0.2) | 0.0 (0.0, 0.3) | 0.1 (0.0, 0.2) | 0.03 |
| Niacin, mg | 0.3 (0.0, 1.5) | 0.3 (0.0, 2.3) | 0.5 (0.1, 1.0) | 0.45 |
| Vitamin B-6, mg | 0.0 (0.0, 0.2) | 0.0 (0.0, 0.1) | 0.1 (0.0, 0.2) | 0.54 |
| Folate, mcg_DFE | 2.3 (0.0, 17.4) | 0.6 (0.0, 11.6) | 8.0 (0.0, 42.5) | 0.01 |
| Vitamin B-12, mcg | 0.0 (0.0, 0.8) | 0.0 (0.0, 0.9) | 0.0 (0.0, 0.0) | 0.73 |
| Vitamin C, mg | 0.1 (0.0, 23.1) | 0.0 (0.0, 10.7) | 13.8 (0.0, 68.7) | <0.001 |
| Vitamin D, mcg | 0.0 (0.0, 0.0) | 0.0 (0.0, 0.3) | 0.0 (0.0, 0.0) | <0.001 |
| Vitamin E, mg | 0.0 (0.0, 0.4) | 0.0 (0.0, 0.3) | 0.0 (0.0, 0.5) | 0.41 |
| Vitamin K, mcg | 0.0 (0.0, 0.7) | 0.0 (0.0, 0.6) | 0.2 (0.0, 4.5) | <0.001 |
| Choline, mg | 3.2 (0.0, 18.8) | 2.0 (0.0, 19.5) | 8.8 (1.4, 17.9) | 0.01 |
| Grains (N) | 437 | 416 | 21 |  |
| Vitamin A, mcg_RAE, | 13.0 (0.0, 165.1) | 14.0 (0.0, 169.2) | 0.0 (0.0, 0.0) | 0.48 |
| Thiamin, mg | 0.3 (0.1, 0.4) | 0.3 (0.1, 0.4) | 0.1 (0.1, 0.2) | <0.01 |
| Riboflavin, mg | 0.2 (0.1, 0.4) | 0.2 (0.1, 0.4) | 0.1 (0.0, 0.1) | <0.01 |
| Niacin, mg | 2.0 (1.4, 5.0) | 2.2 (1.4, 5.0) | 1.3 (0.4, 1.6) | <0.001 |
| Vitamin B-6, mg | 0.1 (0.0, 0.5) | 0.1 (0.1, 0.5) | 0.1 (0.0, 0.1) | 0.08 |
| Folate, mcg_DFE | 71.4 (32.0, 176.1) | 76.3 (37.4, 195.6) | 16.8 (9.6, 24.5) | <0.001 |
| Vitamin B-12, mcg | 0.0 (0.0, 1.2) | 0.0 (0.0, 1.3) | 0.0 (0.0, 0.0) | 0.08 |
| Vitamin C, mg | 0.0 (0.0, 1.0) | 0.0 (0.0, 1.1) | 0.0 (0.0, 0.0) | 0.03 |
| Vitamin D, mcg | 0.0 (0.0, 0.9) | 0.0 (0.0, 1.0) | 0.0 (0.0, 0.0) | 0.27 |
| Vitamin E, mg | 0.2 (0.1, 0.5) | 0.2 (0.1, 0.6) | 0.2 (0.2, 0.3) | 0.91 |
| Vitamin K, mcg | 1.0 (0.4, 2.6) | 1.0 (0.4, 2.8) | 0.7 (0.5, 1.1) | 0.15 |
| Choline, mg | 7.5 (4.1, 14.2) | 7.3 (4.1, 14.1) | 11.6 (7.7, 16.3) | 0.03 |
| Vegetables (N) | 525 | 212 | 313 |  |
| Vitamin A, mcg_RAE, | 35.1 (9.7, 127.5) | 43.1 (5.0, 133.9) | 29.6 (10.6, 120.7) | 0.04 |
| Thiamin, mg | 0.1 (0.0, 0.1) | 0.1 (0.1, 0.1) | 0.0 (0.0, 0.1) | <0.001 |
| Riboflavin, mg | 0.1 (0.0, 0.1) | 0.1 (0.0, 0.1) | 0.0 (0.0, 0.1) | <0.001 |
| Niacin, mg | 0.6 (0.3, 1.2) | 1.2 (0.7, 2.1) | 0.5 (0.3, 0.8) | <0.001 |
| Vitamin B-6, mg | 0.1 (0.1, 0.2) | 0.2 (0.1, 0.3) | 0.1 (0.1, 0.1) | <0.001 |
| Folate, mcg_DFE | 19.5 (12.4, 39.6) | 18.7 (11.6, 43.0) | 20.0 (12.8, 39.2) | 0.17 |
| Vitamin B-12, mcg | 0.0 (0.0, 0.0) | 0.0 (0.0, 0.1) | 0.0 (0.0, 0.0) | 0.20 |
| Vitamin C, mg | 9.0 (3.7, 19.0) | 10.3 (4.7, 17.7) | 8.0 (3.0, 19.5) | 0.50 |
| Vitamin D, mcg | 0.0 (0.0, 0.0) | 0.0 (0.0, 0.1) | 0.0 (0.0, 0.0) | 0.59 |
| Vitamin E, mg | 0.5 (0.3, 1.0) | 0.9 (0.5, 1.4) | 0.4 (0.2, 0.7) | <0.001 |
| Vitamin K, mcg | 13.4 (4.4, 32.0) | 9.7 (4.3, 22.5) | 15.8 (4.6, 38.8) | 0.15 |
| Choline, mg | 14.5 (8.0, 20.3) | 17.5 (13.0, 24.5) | 10.7 (5.7, 15.9) | <0.001 |
| Fruit (N) | 110 | 43 | 67 |  |
| Vitamin A, mcg_RAE, | 4.0 (1.5, 28.9) | 6.0 (1.3, 31.3) | 3.0 (1.5, 24.8) | 0.54 |
| Thiamin, mg | 0.0 (0.0, 0.0) | 0.0 (0.0, 0.1) | 0.0 (0.0, 0.0) | 0.39 |
| Riboflavin, mg | 0.0 (0.0, 0.0) | 0.0 (0.0, 0.0) | 0.0 (0.0, 0.0) | 0.61 |
| Niacin, mg | 0.3 (0.2, 0.5) | 0.3 (0.2, 0.5) | 0.3 (0.2, 0.5) | 0.16 |
| Vitamin B-6, mg | 0.1 (0.0, 0.1) | 0.1 (0.0, 0.1) | 0.1 (0.0, 0.1) | 0.66 |
| Folate, mcg_DFE | 6.3 (3.2, 15.8) | 4.8 (2.4, 14.6) | 9.8 (3.6, 16.5) | 0.08 |
| Vitamin B-12, mcg | 0.0 (0.0, 0.0) | 0.0 (0.0, 0.0) | 0.0 (0.0, 0.0) | 0.75 |
| Vitamin C, mg | 8.5 (2.4, 19.5) | 7.7 (2.2, 15.7) | 9.0 (2.4, 21.1) | 0.36 |
| Vitamin D, mcg | 0.0 (0.0, 0.0) | 0.0 (0.0, 0.0) | 0.0 (0.0, 0.0) | - |
| Vitamin E, mg | 0.2 (0.1, 0.5) | 0.3 (0.1, 0.6) | 0.2 (0.1, 0.4) | 0.28 |
| Vitamin K, mcg | 2.0 (0.7, 4.5) | 1.8 (0.4, 3.0) | 2.3 (0.8, 5.5) | 0.41 |
| Choline, mg | 5.1 (4.2, 8.1) | 5.0 (4.1, 9.5) | 5.1 (4.2, 7.7) | 0.60 |
| Legumes (N) | 80 | 45 | 35 |  |
| Vitamin A, mcg_RAE, | 0.0 (0.0, 1.2) | 0.0 (0.0, 1.3) | 0.0 (0.0, 0.3) | 0.01 |
| Thiamin, mg | 0.1 (0.1, 0.2) | 0.2 (0.1, 0.2) | 0.1 (0.1, 0.2) | 0.53 |
| Riboflavin, mg | 0.1 (0.0, 0.1) | 0.1 (0.1, 0.1) | 0.1 (0.0, 0.1) | 0.15 |
| Niacin, mg | 0.5 (0.4, 0.8) | 0.6 (0.4, 1.2) | 0.4 (0.3, 0.5) | 0.01 |
| Vitamin B-6, mg | 0.1 (0.1, 0.2) | 0.1 (0.1, 0.2) | 0.1 (0.1, 0.1) | 0.12 |
| Folate, mcg_DFE | 75.4 (35.0, 138.5) | 44.2 (27.3, 101.7) | 121.4 (67.2, 139.8) | <0.001 |
| Vitamin B-12, mcg | 0.0 (0.0, 0.0) | 0.0 (0.0, 0.1) | 0.0 (0.0, 0.0) | - |
| Vitamin C, mg | 0.6 (0.0, 1.2) | 0.9 (0.0, 1.6) | 0.5 (0.0, 1.0) | 0.08 |
| Vitamin D, mcg | 0.0 (0.0, 0.0) | 0.0 (0.0, 0.0) | 0.0 (0.0, 0.0) | - |
| Vitamin E, mg | 0.9 (0.7, 1.5) | 1.0 (0.2, 1.5) | 0.9 (0.7, 1.4) | 0.39 |
| Vitamin K, mcg | 8.5 (2.8, 10.1) | 4.1 (1.2, 12.4) | 8.7 (3.2, 10.0) | 0.24 |
| Choline, mg | 30.4 (27.8, 39.0) | 34.6 (29.0, 41.1) | 29.3 (27.5, 31.0) | 0.94 |
| Nuts and Seeds (N) | 77 | 24 | 53 |  |
| Vitamin A, mcg_RAE, | 0.0 (0.0, 0.3) | 0.0 (0.0, 0.0) | 0.0 (0.0, 0.3) | 0.02 |
| Thiamin, mg | 0.1 (0.0, 0.1) | 0.0 (0.0, 0.0) | 0.1 (0.0, 0.2) | <0.01 |
| Riboflavin, mg | 0.1 (0.0, 0.1) | 0.0 (0.0, 0.1) | 0.1 (0.0, 0.1) | 0.05 |
| Niacin, mg | 1.0 (0.4, 2.1) | 1.5 (0.3, 2.8) | 1.0 (0.4, 2.1) | 0.52 |
| Vitamin B-6, mg | 0.1 (0.0, 0.1) | 0.1 (0.0, 0.1) | 0.1 (0.0, 0.1) | 0.18 |
| Folate, mcg_DFE | 17.6 (14.1, 27.2) | 17.1 (13.5, 27.7) | 18.5 (14.3, 27.2) | 0.92 |
| Vitamin B-12, mcg | 0.0 (0.0, 0.0) | 0.0 (0.0, 0.0) | 0.0 (0.0, 0.0) | - |
| Vitamin C, mg | 0.1 (0.0, 0.3) | 0.0 (0.0, 0.2) | 0.1 (0.0,0.3) | 0.33 |
| Vitamin D, mcg | 0.0 (0.0, 0.0) | 0.0 (0.0, 0.0) | 0.0 (0.0, 0.0) | - |
| Vitamin E, mg | 1.6 (0.5, 2.8) | 1.7 (0.9, 2.9) | 1.5 (0.4, 2.7) | 0.23 |
| Vitamin K, mcg | 1.0 (0.1, 3.3) | 0.7 (0.1, 2.7) | 1.0 (0.0, 3.7) | <0.01 |
| Choline, mg | 15.2 (11.2, 16.7) | 11.9 (9.5, 16.5) | 15.5 (12.8, 16.7) | 0.29 |
| Meat (N) | 472 | 458 | 14 |  |
| Vitamin A, mcg_RAE, | 4.3 (0.7, 13.0) | 4.3 (0.7, 12.8) | 19.6 (0.0, 79.0) | <0.001 |
| Thiamin, mg | 0.1 (0.0, 0.2) | 0.1 (0.0, 0.2) | 0.0 (0.0, 0.1) | <0.0001 |
| Riboflavin, mg | 0.2 (0.1, 0.3) | 0.2 (0.1, 0.2) | 0.2 (0.1, 0.4) | 0.04 |
| Niacin, mg | 4.6 (2.5, 6.8) | 4.6 (2.7, 6.9) | 0.0 (0.0, 6.3) | <0.0001 |
| Vitamin B-6, mg | 0.2 (0.1, 0.4) | 0.3 (0.1, 0.4) | 0.1 (0.1, 0.4) | <0.0001 |
| Folate, mcg_DFE | 5.4 (2.5, 11.2) | 5.3 (2.4, 10.7) | 13.4 (8.8, 22.0) | 0.19 |
| Vitamin B-12, mcg | 0.4 (0.3, 0.8) | 0.4 (0.3, 0.8) | 0.4 (0.3, 2.4) | 0.92 |
| Vitamin C, mg | 0.0 (0.0, 0.0) | 0.0 (0.0, 0.0) | 0.0 (0.0, 0.0) | - |
| Vitamin D, mcg | 0.2 (0.1, 0.3) | 0.2 (0.1, 0.3) | 0.2 (0.0, 1.0) | <0.001 |
| Vitamin E, mg | 0.3 (0.2, 0.6) | 0.3 (0.2, 0.6) | 0.5 (0.3, 0.6) | 0.08 |
| Vitamin K, mcg | 1.0 (0.0, 3.6) | 1.0 (0.0 3.6) | 0.2 (0.1, 1.3) | <0.0001 |
| Choline, mg | 62.3 (40.7, 83.2) | 61.5 (40.7, 82.2) | 113.4 (87.5, 117.5) | 0.38 |
| Seafood (N) | 193 | 176 | 17 |  |
| Vitamin A, mcg_RAE, | 27.1 (11.1, 45.2) | 27.2 (11.3, 44.6) | 18.2 (10.3, 47.3) | 0.82 |
| Thiamin, mg | 0.1 (0.0, 0.2) | 0.1 (0.0, 0.2) | 0.0 (0.0, 0.1) | <0.001 |
| Riboflavin, mg | 0.1 (0.1, 0.2) | 0.1 (0.1, 0.2) | 0.1 (0.0, 0.1) | 0.01 |
| Niacin, mg | 2.9 (1.4, 5.6) | 3.4 (1.5, 5.7) | 1.2 (0.7, 1.4) | <0.001 |
| Vitamin B-6, mg | 0.2 (0.1, 0.4) | 0.2 (0.1, 0.4) | 0.1 (0.0, 0.1) | <0.001 |
| Folate, mcg_DFE | 13.6 (6.8, 29.1) | 15.8 (6.9, 30.5) | 5.1 (3.4, 12.5) | <0.001 |
| Vitamin B-12, mcg | 2.1 (1.3, 3.7) | 2.1 (1.4, 3.7) | 1.2 (0.7, 4.9) | 0.31 |
| Vitamin C, mg | 0.3 (0.0, 0.6) | 0.3 (0.0, 0.6) | 0.0 (0.0, 0.3) | 0.56 |
| Vitamin D, mcg | 1.7 (0.1, 6.1) | 2.0 (0.3, 6.4) | 0.1 (0.0, 0.2) | <0.01 |
| Vitamin E, mg | 1.1 (0.7, 1.5) | 1.1 (0.7, 1.6) | 0.7 (0.6, 1.0) | <0.01 |
| Vitamin K, mcg | 2.9 (0.3, 6.8) | 3.3 (0.4, 7.0) | 0.9 (0.2, 2.6) | 0.06 |
| Choline, mg | 75.9 (55.3, 91.4) | 85.7 (58.1, 92.3) | 44.2 (43.9, 53.9) | <0.001 |
| Dairy (N) | 196 | 174 | 22 |  |
| Vitamin A, mcg_RAE, | 54.1 (22.4, 133.0) | 52.2 (21.0, 114.7) | 135.0 (23.8, 141.5) | 0.53 |
| Thiamin, mg | 0.0 (0.0, 0.1) | 0.0 (0.0, 0.1) | 0.1 (0.1, 0.1) | <0.01 |
| Riboflavin, mg | 0.2 (0.1, 0.3) | 0.2 (0.1, 0.3) | 0.3 (0.3, 0.4) | <0.01 |
| Niacin, mg | 0.2 (0.0, 0.3) | 0.1 (0.0, 0.3) | 0.2 (0.2, 0.3) | 0.01 |
| Vitamin B-6, mg | 0.1 (0.0, 0.1) | 0.0 (0.0, 0.1) | 0.1 (0.1, 0.1) | 0.01 |
| Folate, mcg_DFE | 4.68 (1.7, 10.4) | 4.2 (1.4, 7.5) | 11.0 (4.9, 18.7) | <0.01 |
| Vitamin B-12, mcg | 0.5 (0.2, 1.0) | 0.4 (0.2, 1.0) | 1.0 (0.8, 1.4) | <0.01 |
| Vitamin C, mg | 0.0 (0.0, 1.0) | 0.0 (0.0, 0.5) | 1.3 (0.0, 1.5) | 0.40 |
| Vitamin D, mcg | 0.3 (0.0, 2.5) | 0.2 (0.0, 2.2) | 2.0 (0.0, 2.7) | 0.02 |
| Vitamin E, mg | 0.1 (0.0, 0.1) | 0.1 (0.0, 0.1) | 0.0 (0.0, 0.1) | 0.05 |
| Vitamin K, mcg | 0.3 (0.2, 0.5) | 0.4 (0.2, 0.5) | 0.2 (0.0, 0.3) | 0.13 |
| Choline, mg | 19.0 (3.2, 39.0) | 15.1 (3.2, 39.0) | 31.2 (22.8, 42.5) | 0.01 |
| Fats and Oils (N) | 68 | 60 | 8 |  |
| Vitamin A, mcg_RAE, | 2.8 (0.3, 64.8) | 3.6 (0.5, 80.4) | 0.0 (0.0, 0.0) | - |
| Thiamin, mg | 0.0 (0.0, 0.0) | 0.0 (0.0, 0.0) | 0.0 (0.0, 0.0) | - |
| Riboflavin, mg | 0.0 (0.0, 0.0) | 0.0 (0.0, 0.0) | 0.0 (0.0, 0.0) | - |
| Niacin, mg | 0.0 (0.0, 0.0) | 0.0 (0.0, 0.0) | 0.0 (0.0, 0.0) | - |
| Vitamin B-6, mg | 0.0 (0.0, 0.0) | 0.0 (0.0, 0.0) | 0.0 (0.0, 0.0) | - |
| Folate, mcg_DFE | 0.4 (0.0, 0.9) | 0.6 (0.1, 1.2) | 0.0 (0.0, 0.0) | - |
| Vitamin B-12, mcg | 0.0 (0.0, 0.0) | 0.0 (0.0, 0.0) | 0.0 (0.0, 0.0) | - |
| Vitamin C, mg | 0.0 (0.0, 0.1) | 0.0 (0.0, 0.1) | 0.0 (0.0, 0.0) | - |
| Vitamin D, mcg | 0.0 (0.0, 0.0) | 0.0 (0.0, 0.0) | 0.0 (0.0, 0.0) | - |
| Vitamin E, mg | 0.4 (0.2, 1.4) | 0.4 (0.2, 1.2) | 2.0 (0.9, 4.0) | 0.03 |
| Vitamin K, mcg | 3.9 (0.7, 14.2) | 4.1 (0.7,14.2) | 2.0 (0.9, 9.2) | 0.50 |
| Choline, mg | 1.5 (1.1, 3.1) | 1.7 (1.4, 4.5) | 0.0 (0.0, 0.0) | <0.001 |
| Mixed Dishes (N) | 1510 | 1441 | 69 |  |
| Vitamin A, mcg_RAE, | 65.0 (18.1, 142.6) | 67.1 (18.6, 143.4) | 32.4 (8.5, 129.2) | 0.03 |
| Thiamin, mg | 0.2 (0.1, 0.4) | 0.2 (0.1, 0.4) | 0.2 (0.1, 0.2) | <0.0001 |
| Riboflavin, mg | 0.3 (0.1, 0.4) | 0.3 (0.2, 0.4) | 0.1 (0.1, 0.2) | <0.0001 |
| Niacin, mg | 3.5 (1.9, 5.8) | 3.6 (2.0, 5.8) | 2.3 (0.9, 3.2) | <0.0001 |
| Vitamin B-6, mg | 0.2 (0.1, 0.3) | 0.2 (0.1, 0.3) | 0.2 (0.1, 0.2) | <0.0001 |
| Folate, mcg_DFE | 63.2 (27.7, 114.8) | 66.4 (30.0, 115.9) | 25.6 (17.5, 45.0) | <0.0001 |
| Vitamin B-12, mcg | 0.5 (0.2, 1.1) | 0.6 (0.2, 1.1) | 0.0 (0.0, 0.3) | <0.001 |
| Vitamin C, mg | 2.2 (0.3, 8.7) | 2.2 (0.3, 8.5) | 4.7 (1.0, 12.2) | <0.01 |
| Vitamin D, mcg | 0.2 (0.0, 0.5) | 0.2 (0.0, 0.6) | 0.0 (0.0, 0.0) | 0.35 |
| Vitamin E, mg | 1.1 (0.6, 1.8) | 1.1 (0.6, 1.8) | 0.8 (0.5, 1.8) | 0.99 |
| Vitamin K, mcg | 10.5 (4.8, 21.8) | 10.5 (4.8, 21.8) | 10.1 (4.7, 20.3) | 0.12 |
| Choline, mg | 44.9 (27.6, 72.2) | 45.8 (28.5, 72.8) | 24.5 (14.2, 39.8) | <0.0001 |
| Sauces and Condiments (N) | 178 | 169 | 9 |  |
| Vitamin A, mcg_RAE, | 1.7 (0.0, 13.2) | 2.6 (0.0, 15.9) | 0.2 (0.0, 0.2) | <0.001 |
| Thiamin, mg | 0.0 (0.0, 0.0) | 0.0 (0.0, 0.0) | 0.0 (0.0, 0.0) | 0.27 |
| Riboflavin, mg | 0.0 (0.0, 0.0) | 0.0 (0.0, 0.0) | 0.0 (0.0, 0.0) | <0.001 |
| Niacin, mg | 0.1 (0.0, 0.2) | 0.1 (0.0, 0.2) | 0.0 (0.0, 0.0) | 0.05 |
| Vitamin B-6, mg | 0.0 (0.0, 0.0) | 0.0 (0.0, 0.0) | 0.0 (0.0, 0.1) | 0.20 |
| Folate, mcg_DFE | 1.6 (0.4, 5.8) | 1.8 (0.4, 6.0) | 0.8 (0.7, 1.6) | 0.20 |
| Vitamin B-12, mcg | 0.0 (0.0, 0.0) | 0.0 (0.0, 0.0) | 0.0 (0.0, 0.0) | - |
| Vitamin C, mg | 0.3 (0.0, 1.6) | 0.2 (0.0, 1.2) | 2.4 (1.6, 3.1) | 0.03 |
| Vitamin D, mcg | 0.0 (0.0, 0.0) | 0.0 (0.0, 0.0) | 0.0 (0.0, 0.0) | - |
| Vitamin E, mg | 0.1 (0.0, 0.6) | 0.1 (0.0, 0.6) | 0.0 (0.0, 0. | 0.02 |
| Vitamin K, mcg | 0.4 (0.0, 5.0) | 0.6 (0.1, 5.0) | 0.0 (0.0, 0.1) | 0.01 |
| Choline, mg | 2.3 (0.5, 5.6) | 2.5 (0.6, 5.9) | 0.4 (0.4, 1.2) | 0.01 |
| Snacks and Desserts (N) | 680 | 671 | 9 |  |
| Vitamin A, mcg_RAE, | 3.0 (0.0, 33.3) | 3.1 (0.0, 34.9) | 0.0 (0.0, 0.7) | 0.25 |
| Thiamin, mg | 0.1 (0.0, 0.1) | 0.1 (0.0, 0.1) | 0.0 (0.0, 0.0) | 0.04 |
| Riboflavin, mg | 0.1 (0.0, 0.2) | 0.1 (0.0, 0.2) | 0.0 (0.0, 0.0) | 0.02 |
| Niacin, mg | 0.8 (0.3, 1.3) | 0.8 (0.3, 1.3) | 0.9 (0.6, 1.1) | 0.51 |
| Vitamin B-6, mg | 0.0 (0.0, 0.1) | 0.0 (0.0, 0.1)) | 0.0 (0.0, 0.1) | 0.31 |
| Folate, mcg_DFE | 20.6 (4.8, 43.3) | 21.4 (4.8, 43.4) | 6.3 (4.2, 6.3) | 0.05 |
| Vitamin B-12, mcg | 0.0 (0.0, 0.1) | 0.0 (0.0, 0.1) | 0.0 (0.0, 0.0) | - |
| Vitamin C, mg | 0.1 (0.0, 0.7) | 0.1 (0.0, 0.8) | 0.0 (0.0, 0.0) | - |
| Vitamin D, mcg | 0.0 (0.0, 0.1) | 0.0 (0.0, 0.1) | 0.0 (0.0, 0.0) | - |
| Vitamin E, mg | 0.5 (0.2, 1.0) | 0.5 (0.2, 1.0) | 0.2 (0.1, 0.3) | 0.20 |
| Vitamin K, mcg | 1.8 (0.6, 4.6) | 1.9 (0.6, 4.7) | 1.6 (0.3, 2.5) | 0.27 |
| Choline, mg | 7.1 (4.0, 18.0) | 7.2 (4.1, 18.5) | 4.2 (3.6, 4.9) | 0.22 |

1. Abbreviations: Reference amount customarily consumed (RACC), grams (g), milligrams (mg), microgram (mcg), interquartile range (IQR), retinol activity equivalent (RAE), dietary folate equivalent (DFE)
2. P-values from t-tests comparing mean log-transformed nutrient values between FDA-aligned and FDA-unaligned groups

**Supplemental Table 19.** Mean Minerals per RACC of FDA-Aligned and FDA-Unaligned Foods and Beverages Consumed in the United States NHANES/FNDDS 2017-2018, Overall and Stratified by Nova ^1^

|  | **Overall**  **Median (IQR)** | **FDA Unaligned**  **Median (IQR)** | **FDA Aligned**  **Median (IQR)** | **p-value^2^** |
| --- | --- | --- | --- | --- |
| Overall (N) | 4949 | 4214 | 735 |  |
| Calcium, mg | 31.5 (12.6, 98.5) | 36.6 (12.8, 107.3) | 22.2 (11.2, 38.0) | <0.001 |
| Phosphorus, mg | 105.3 (39.6, 205.7) | 121.5 (48.2, 221.3) | 42.2 (19.4, 108.6) | <0.001 |
| Magnesium, mg | 21.3 (10.8, 36.2) | 21.6 (11.2, 36.3) | 17.7 (9.5, 34.0) | 0.26 |
| Iron, mg | 1.0 (0.4, 2.1) | 1.1 (0.4, 2.3) | 0.5 (0.3, 1.0) | <0.001 |
| Zinc, mg | 0.7 (0.3, 1.6) | 0.8 (0.3, 1.7) | 0.3 (0.1, 0.8) | <0.001 |
| Copper, mg | 0.1 (0.0, 0.2) | 0.1 (0.0, 0.2) | 0.1 (0.0, 0.1) | 0.01 |
| Selenium, mcg | 7.4 (1.1, 20.8) | 9.6 (2.3, 23.5) | 0.7 (0.2, 3.9) | <0.001 |
| Potassium, mg | 192.6 (80.9, 344.7) | 196.0 (77.2, 358.8) | 177.5 (112.8, 272.2) | 0.03 |
| Sodium, mg | 268.8 (117.6, 513.0) | 323.7 (152.6, 578.3) | 98.8 (7.2, 159.4) | <0.001 |
| Minimally Processed Foods (N) | 2257 | 1628 | 629 |  |
| Calcium, mg | 28.8 (14.5, 71.4) | 35.0 (15.4, 85.2) | 22.2 (11.2, 36.0) | <0.001 |
| Phosphorus, mg | 136.0 (51.2, 226.0) | 171.7 (93.4, 251.6) | 37.2 (18.4, 99.3) | <0.001 |
| Magnesium, mg | 24.7 (15.0, 37.2) | 26.9 (17.0, 38.1) | 17.4 (9.5, 30.6) | <0.001 |
| Iron, mg | 1.0 (0.5, 1.9) | 1.3 (0.7, 2.1) | 0.5 (0.2, 1.0) | <0.001 |
| Zinc, mg | 0.9 (0.4, 1.6) | 1.2 (0.6, 2.0) | 0.3 (0.1, 0.7) | <0.001 |
| Copper, mg | 0.1 (0.1, 0.2) | 0.1 (0.1, 0.2) | 0.1 (0.0, 0.1) | <0.001 |
| Selenium, mcg | 10.3 (1.3, 25.5) | 16.4 (7.0, 32.4) | 0.6 (0.2, 3.3) | <0.001 |
| Potassium, mg | 242.5 (142.5, 386.3) | 274.1 (159.6, 409.1) | 183.0 (119.5, 279.8) | <0.001 |
| Sodium, mg | 290.1 (128.0, 489.3) | 409.1 (256.9, 561.9) | 100.5 (7.2, 155.5) | <0.001 |
| Processed Culinary Ingredients (N) | 52 | 43 | 9 |  |
| Calcium, mg | 3.1 (0.1, 19.8) | 3.6 (0.6, 21.4) | 0.0 (0.0, 0.0) | 0.72 |
| Phosphorus, mg | 2.3 (0.0, 29.5) | 3.4 (0.3, 32.5) | 0.0 (0.0, 0.0) | - |
| Magnesium, mg | 0.5 (0.0, 3.8) | 1.0 (0.1, 4.3) | 0.0 (0.0, 0.0) | - |
| Iron, mg | 0.0 (0.0, 0.1) | 0.0 (0.0, 0.1) | 0.0 (0.0, 0.0) | 0.97 |
| Zinc, mg | 0.0 (0.0, 0.1) | 0.0 (0.0, 0.1) | 0.0 (0.0, 0.0) | - |
| Copper, mg | 0.0 (0.0, 0.0) | 0.0 (0.0, 0.0) | 0.0 (0.0, 0.0) | - |
| Selenium, mcg | 0.1 (0.0, 0.9) | 0.3 (0.1, 1.7) | 0.0 (0.0, 0.0) | - |
| Potassium, mg | 8.0 (0.2, 43.4) | 18.2 (2.5, 48.5) | 0.0 (0.0, 0.0) | 0.34 |
| Sodium, mg | 8.4 (0.1, 54.9) | 14.4 (1.1, 88.8) | 0.0 (0.0, 0.0) | 0.40 |
| Processed Foods (N) | 325 | 277 | 48 |  |
| Calcium, mg | 19.2 (7.6, 70.2) | 19.5 (6.7, 90.3) | 19.1 (14.3, 37.6) | 0.46 |
| Phosphorus, mg | 72.7 (27.0, 127.1) | 70.8 (25.5, 137.1) | 80.4 (37.5, 124.0) | 0.39 |
| Magnesium, mg | 12.1 (5.7, 32.0) | 11.3 (5.5, 27.3) | 25.3 (11.1, 51.2) | <0.001 |
| Iron, mg | 0.5 (0.2, 1.1) | 0.4 (0.1, 1.0) | 0.7 (0.4, 1.1) | 0.01 |
| Zinc, mg | 0.5 (0.2, 0.9) | 0.5 (0.1, 0.9) | 0.6 (0.3, 0.9) | 0.10 |
| Copper, mg | 0.1 (0.0, 0.1) | 0.0 (0.0, 0.1) | 0.1 (0.1, 0.3) | <0.001 |
| Selenium, mcg | 2.7 (0.5, 9.8) | 3.0 (0.5, 10.8) | 0.9 (0.5, 2.7) | 0.02 |
| Potassium, mg | 123.5 (56.3, 216.0) | 111.6 (45.0, 223.2) | 161.1 (112.5, 198.5) | 0.01 |
| Sodium, mg | 179.4 (55.0, 378.9) | 239.2 (64.8, 447.4) | 103.0 (43.3, 177.7) | <0.01 |
| Ultra-Processed Foods (N) | 2169 | 2123 | 46 |  |
| Calcium, mg | 37.8 (10.5, 116.8) | 38.0 (10.4, 118.0) | 37.2 (14.0, 79.2) | 0.84 |
| Phosphorus, mg | 82.8 (33.5, 188.5) | 83.8 (33.5, 187.0) | 67.3 (37.2, 191.3) | 0.41 |
| Magnesium, mg | 18.2 (8.4, 34.7) | 18.0 (8.2, 34.5) | 28.5 (19.7, 57.0) | <0.01 |
| Iron, mg | 1.0 (0.4, 2.4) | 1.1 (0.4, 2.4) | 0.5 (0.3, 1.7) | 0.70 |
| Zinc, mg | 0.6 (0.2, 1.5) | 0.6 (0.2, 1.5) | 0.6 (0.2, 1.5) | 0.63 |
| Copper, mg | 0.1 (0.0, 0.1) | 0.1 (0.0, 0.1) | 0.1 (0.0, 0.1) | 0.10 |
| Selenium, mcg | 5.9 (1.1, 17.9) | 5.9 (1.2, 17.9) | 4.9 (0.4, 11.9) | 0.81 |
| Potassium, mg | 133.2 (55.9, 293.5) | 133.2 (55.7, 293.5) | 142.5 (68.6, 327.5) | 0.28 |
| Sodium, mg | 249.0 (113.2, 579.0) | 252.7 (116.3, 590.5) | 81.5 (40.6, 162.0) | <0.001 |

1. Abbreviations: Reference amount customarily consumed (RACC), milligrams (mg), microgram (mcg), interquartile range (IQR)
2. P-values from t-tests comparing mean log-transformed nutrient values between FDA-aligned and FDA-unaligned groups

**Supplemental Table 20.** Mean Minerals per RACC of FDA-Aligned and FDA-Unaligned Foods and Beverages Consumed in the United States NHANES/FNDDS 2017-2018, Overall and Stratified by Food Category^1^

|  | **Overall** | **FDA Unaligned** | **FDA Aligned** | **p-value^2^** |
| --- | --- | --- | --- | --- |
| Beverages (N) | 422 | 324 | 98 |  |
| Calcium, mg | 18.9 (7.2, 106.6) | 15.4 (5.0, 149.1) | 27.3 (7.2, 97.2) | 0.24 |
| Phosphorus, mg | 23.7 (3.6, 84.3) | 22.1 (3.7, 93.0) | 28.6 (3.6, 69.4) | 0.36 |
| Magnesium, mg | 14.4 (3.6, 34.7) | 12.4 (2.4, 34.7) | 17.4 (9.9, 34.7) | 0.08 |
| Iron, mg | 0.2 (0.1, 0.6) | 0.1 (0.1, 0.5) | 0.3 (0.1, 0.6) | 0.07 |
| Zinc, mg | 0.1 (0.0, 0.5) | 0.1 (0.0, 0.5) | 0.1 (0.1, 0.4) | 0.63 |
| Copper, mg | 0.0 (0.0, 0.1) | 0.0 (0.0, 0.1) | 0.0 (0.0, 0.1) | 0.16 |
| Selenium, mcg | 0.3 (0.0, 2.2) | 0.4 (0.0, 2.6) | 0.2 (0.0, 1.0) | 0.23 |
| Potassium, mg | 110.3 (28.8, 340.9) | 76.9 (18.6, 291.2) | 250.5 (124.0, 446.6) | <0.001 |
| Sodium, mg | 25.2 (10.8, 108.0) | 30.5 (12.6, 130.2) | 12.4 (6.5, 47.1) | <0.001 |
| Grains (N) | 437 | 416 | 21 |  |
| Calcium, mg | 39.4 (15.1, 106.4) | 42.0 (14.5, 107.4) | 35.6 (28.5, 58.7) | 0.67 |
| Phosphorus, mg | 76.3 (44.7, 149.1) | 72.9 (43.5, 148.8) | 120.0 (76.3, 190.0) | 0.17 |
| Magnesium, mg | 20.7 (11.3, 41.6) | 19.4 (11.0, 39.3) | 43.2 (27.7, 57.6) | 0.02 |
| Iron, mg | 1.8 (1.1, 6.1) | 1.9 (1.1, 6.1) | 1.3 (0.9, 2.4) | 0.17 |
| Zinc, mg | 0.7 (0.4, 1.5) | 0.7 (0.4, 1.5) | 1.2 (0.6, 1.5) | 0.62 |
| Copper, mg | 0.1 (0.1, 0.2) | 0.1 (0.1, 0.1) | 0.2 (0.1, 0.2) | 0.02 |
| Selenium, mcg | 8.8 (5.2, 13.0) | 8.5 (5.1, 13.0) | 11.5 (7.1, 12.7) | 0.74 |
| Potassium, mg | 85.5 (52.5, 167.7) | 83.1 (51.8, 167.1) | 122.4 (83.5, 192.0) | 0.41 |
| Sodium, mg | 218.4 (165.6, 312.3) | 224.4 (168.3, 316.2) | 162.0 (123.6, 170.4) | <0.001 |
| Vegetables (N) | 525 | 212 | 313 |  |
| Calcium, mg | 23.3 (13.2, 37.0) | 26.3 (13.3, 50.0) | 20.8 (13.2, 30.8) | <0.001 |
| Phosphorus, mg | 44.0 (23.7, 63.8) | 61.9 (44.3, 102.4) | 30.4 (18.6, 50.2) | <0.001 |
| Magnesium, mg | 17.0 (10.4, 26.1) | 22.4 (14.9, 40.8) | 14.0 (8.8, 20.5) | <0.001 |
| Iron, mg | 0.6 (0.4, 0.9) | 0.6 (0.4, 1.1) | 0.5 (0.3, 0.7) | <0.001 |
| Zinc, mg | 0.3 (0.2, 0.5) | 0.4 (0.3, 0.6) | 0.2 (0.1, 0.4) | <0.001 |
| Copper, mg | 0.1 (0.0, 0.1) | 0.1 (0.1, 0.2) | 0.1 (0.0, 0.1) | <0.001 |
| Selenium, mcg | 0.6 (0.3, 1.3) | 0.9 (0.5, 3.9) | 0.5 (0.2, 0.8) | <0.001 |
| Potassium, mg | 207.1 (129.3, 339.5) | 326.7 (179.1, 478.5) | 166.4 (113.4, 244.2) | <0.001 |
| Sodium, mg | 146.1 (104.3, 265.0) | 283.9 (194.5, 371.6) | 115.6 (41.6, 148.1) | <0.001 |
| Fruits (N) | 110 | 43 | 67 |  |
| Calcium, mg | 10.4 (6.0, 17.5) | 8.4 (5.0, 18.1) | 11.0 (6.0, 17.5) | 0.51 |
| Phosphorus, mg | 15.0 (9.8, 25.5) | 13.8 (10.0, 20.0) | 16.5 (9.0, 26.8) | 0.29 |
| Magnesium, mg | 10.0 (7.2, 15.4) | 10.0 (6.3, 18.9) | 10.2 (8.3, 15.3) | 0.52 |
| Iron, mg | 0.3 (0.2, 0.4) | 0.4 (0.3, 0.4) | 0.3 (0.2, 0.4) | 0.47 |
| Zinc, mg | 0.1 (0.1, 0.2) | 0.1 (0.1, 0.2) | 0.1 (0.1, 0.2) | 0.36 |
| Copper, mg | 0.1 (0.0, 0.1) | 0.1 (0.0, 0.1) | 0.1 (0.0, 0.1) | 0.91 |
| Selenium, mcg | 0.4 (0.2, 0.6) | 0.5 (0.2, 0.6) | 0.3 (0.1, 0.5) | 0.02 |
| Potassium, mg | 147.4 (105.0, 193.6) | 145.3 (93.8, 168.3) | 151.1 (105.8, 208.8) | 0.17 |
| Sodium, mg | 2.5 (0.8, 6.3) | 5 (2.5, 8.4) | 1.3 (0.8, 4.0) | <0.01 |
| Legumes (N) | 80 | 45 | 35 |  |
| Calcium, mg | 41.9 (31.0, 51.9) | 47.2 (35.9, 64.8) | 35.9 (23.0, 43.2) | 0.02 |
| Phosphorus, mg | 133.4 (119.6, 156.4) | 139.5 (119.7, 166.4) | 126.0 (114.6, 143.5) | 0.65 |
| Magnesium, mg | 45.0 (38.2, 53.8) | 45.5 (32.5, 53.3) | 45.0 (38.6, 54.3) | 0.39 |
| Iron, mg | 2.1 (1.8, 2.5) | 2.1 (1.8, 2.5) | 2.1 (1.8, 2.6) | 0.19 |
| Zinc, mg | 0.9 (0.8, 1.1) | 0.9 (0.8, 1.1) | 1.0 (0.8, 1.1) | 0.21 |
| Copper, mg | 0.2 (0.2, 0.3) | 0.2 (0.2, 0.3) | 0.2 (0.2, 0.3) | 0.17 |
| Selenium, mcg | 3.2 (1.5, 5.5) | 4.6 (1.7, 6.2) | 2.2 (1.1, 4.2) | 0.03 |
| Potassium, mg | 358.0 (250.2, 415.4) | 389.7 (233.1, 440.7) | 345.0 (260.1, 401.1) | 0.19 |
| Sodium, mg | 209.7 (200.6, 354.3) | 313.5 (279.9, 455.0) | 200.6 (199.6, 204.2) | <0.01 |
| Nuts, Seeds (N) | 77 | 24 | 53 |  |
| Calcium, mg | 19.6 (15.4, 27.4) | 15.7 (12.3, 19.6) | 23.0 (16.8, 30.0) | <0.01 |
| Phosphorus, mg | 111.2 (86.5, 132.4) | 90.0 (60.3, 117.9) | 126.0 (101.6, 137.2) | 0.02 |
| Magnesium, mg | 49.8 (33.3, 70.0) | 38.9 (29.1, 53.9) | 55.2 (35.3, 70.6) | 0.03 |
| Iron, mg | 0.9 (0.6, 1.1) | 0.6 (0.5, 1.0) | 1.0 (0.7, 1.2) | 0.04 |
| Zinc, mg | 0.9 (0.7, 1.3) | 0.8 (0.5, 1.0) | 0.9 (0.8, 1.3) | 0.07 |
| Copper, mg | 0.3 (0.2, 0.4) | 0.2 (0.1, 0.3) | 0.3 (0.3, 0.4) | <0.01 |
| Selenium, mcg | 2.6 (1.0, 9.6) | 2.2 (0.9, 3.7) | 2.7 (1.1, 10.1) | 0.25 |
| Potassium, mg | 172.2 (132.2, 203.3) | 135.9 (100.4, 202.3) | 177.5 (153.4, 203.3) | <0.01 |
| Sodium, mg | 49.3 (2.0, 107.2) | 107.8 (76.7, 134.0) | 5.0 (1.7, 72.6) | <0.001 |
| Meat (N) | 472 | 458 | 14 |  |
| Calcium, mg | 11.9 (6.8, 21.4) | 11.9 (6.8, 21.4) | 16.7 (5.1, 28.0) | 0.76 |
| Phosphorus, mg | 160.3 (111.0, 220.4) | 162.4 (113.4, 221.3) | 98.8 (66.3, 205.7) | <0.001 |
| Magnesium, mg | 17.2 (10.6, 24.1) | 17.6 (10.8, 24.7) | 6.0 (3.6, 23.8) | <0.0001 |
| Iron, mg | 0.8 (0.6, 1.4) | 0.8 (0.6, 1.4) | 0.9 (0.6, 3.3) | 0.70 |
| Zinc, mg | 1.4 (1.1, 2.6) | 1.5 (1.1, 2.6) | 0.6 (0.4, 3.1) | <0.001 |
| Copper, mg | 0.1 (0.0, 0.1) | 0.1 (0.0, 0.1) | 0.0 (0.0, 0.2) | 0.03 |
| Selenium, mcg | 19.6 (14.1, 29.9) | 19.9 (14.2, 30.5) | 15.0 (9.5, 15.3) | 0.01 |
| Potassium, mg | 217.7 (163.9, 329.1) | 218.9 (167.6, 332.9) | 69.0 (53.8, 264.4) | <0.0001 |
| Sodium, mg | 410.4 (287.0, 515.5) | 416.5 (300.1, 518.6) | 128.0 (71.0, 196.0) | <0.0001 |
| Seafood (N) | 193 | 176 | 17 |  |
| Calcium, mg | 41.8 (15.8, 54.7) | 42.9 (15.3, 55.4) | 27.4 (18.2, 39.3) | 0.05 |
| Phosphorus, mg | 255.4 (163.6, 311.9) | 262.2 (190.4, 314.1) | 134.5 (124.3, 149.9) | <0.001 |
| Magnesium, mg | 30.5 (22.2, 37.3) | 31.6 (25.0, 37.3) | 15.4 (13.7, 18.8) | <0.001 |
| Iron, mg | 0.8 (0.4, 1.3) | 0.8 (0.4, 1.3) | 0.5 (0.4, 1.3) | 0.36 |
| Zinc, mg | 0.7 (0.5, 1.0) | 0.7 (0.5, 1.0) | 0.6 (0.3, 0.8) | 0.29 |
| Copper, mg | 0.1 (0.1, 0.1) | 0.1 (0.1, 0.1) | 0.1 (0.0, 0.2) | 0.44 |
| Selenium, mcg | 34.7 (20.1, 46.2) | 36.7 (20.7, 47.5) | 20.8 (17.9, 25.5) | <0.01 |
| Potassium, mg | 327.7 (159.6, 419.2) | 346.3 (198.9, 424.9) | 140.2 (57.6, 167.6) | <0.001 |
| Sodium, mg | 437.3 (341.7, 467.8) | 443.0 (405.1, 471.8) | 197.8 (50.2, 212.0) | <0.001 |
| Dairy (N) | 196 | 174 | 22 |  |
| Calcium, mg | 166.1 (99.7, 300.5) | 150.2 (82.5, 283.0) | 307.4 (201.8, 311.1) | 0.01 |
| Phosphorus, mg | 165.2 (90.3, 244.8) | 155.9 (74.6, 240.6) | 244.8 (205.5, 251.3) | <0.01 |
| Magnesium, mg | 15.0 (5.4, 29.3) | 10.2 (4.8, 29.8) | 28.9 (16.5, 29.3) | 0.01 |
| Iron, mg | 0.1 (0.0, 0.2) | 0.1 (0.0, 0.2) | 0.1 (0.0, 0.1) | 0.97 |
| Zinc, mg | 0.8 (0.5, 1.1) | 0.7 (0.4, 1.1) | 1.0 (0.9, 1.5) | 0.01 |
| Copper, mg | 0.0 (0.0, 0.0) | 0.0 (0.0, 0.1) | 0.0 (0.0, 0.0) | 0.76 |
| Selenium, mcg | 4.5 (2.7, 5.7) | 4.3 (2.6, 5.4) | 5.6 (5.1, 7.4) | 0.01 |
| Potassium, mg | 151.3 (30.5, 388.0) | 100.0 (28.1, 384.0) | 388.0 (211.5, 405.0) | <0.001 |
| Sodium, mg | 119.0 (56.9, 161.2) | 125.7 (58.3, 164.0) | 100.0 (54.0, 123.1) | 0.92 |
| Fats and Oils (N) | 68 | 60 | 8 |  |
| Calcium, mg | 3.1 (0.6, 7.2) | 3.4 (1.6, 7.9) | 0.0 (0.0, 0.0) | - |
| Phosphorus, mg | 3.4 (1.3, 7.3) | 3.4 (1.9, 8.4) | 0.0 (0.0, 0.0) | - |
| Magnesium, mg | 0.3 (0.1, 1.5) | 0.6 (0.2, 1.6) | 0.0 (0.0, 0.0) | - |
| Iron, mg | 0.0 (0.0, 0.1) | 0.0 (0.0, 0.1) | 0.0 (0.0, 0.0) | 0.92 |
| Zinc, mg | 0.0 (0.0, 0.1) | 0.0 (0.0, 0.1) | 0.0 (0.0, 0.0) | - |
| Copper, mg | 0.0 (0.0, 0.0) | 0.0 (0.0, 0.0) | 0.0 (0.0, 0.0) | - |
| Selenium, mcg | 0.2 (0.0, 0.5) | 0.3 (0.0, 0.5) | 0.0 (0.0, 0.0) | - |
| Potassium, mg | 6.0 (2.9, 19.4) | 8.0 (3.6, 23.2) | 0.0 (0.0, 0.0) | - |
| Sodium, mg | 94.4 (64.2, 268.5) | 131.9 (81.8, 269.8) | 0.0 (0.0, 0.0) | - |
| Mixed Dishes (N) | 1510 | 1441 | 69 |  |
| Calcium, mg | 82.6 (37.5, 207.6) | 87.0 (39.0, 213.5) | 28.9 (12.8, 50.0) | <0.0001 |
| Phosphorus, mg | 205.2 (133.5, 316.8) | 208.3 (138.5, 323.3) | 127.8 (99.6, 202.3) | <0.0001 |
| Magnesium, mg | 34.1 (22.1, 47.4) | 33.8 (22.0, 46.8) | 45.4 (23.7, 51.5) | 0.06 |
| Iron, mg | 2.2 (1.4, 3.0) | 2.2 (1.5, 3.0) | 0.8 (0.7, 2.1) | <0.0001 |
| Zinc, mg | 1.6 (1.0, 2.6) | 1.7 (1.0, 2.7) | 0.9 (0.6, 1.2) | <0.0001 |
| Copper, mg | 0.1 (0.1, 0.2) | 0.1 (0.1, 0.2) | 0.1 (0.1, 0.2) | 0.06 |
| Selenium, mcg | 22.2 (12.5, 37.5) | 23.1 (13.4, 37.8) | 7.8 (6.5, 18.1) | <0.0001 |
| Potassium, mg | 310.5 (208.6, 462.8) | 315.2 (212.3, 465.4) | 226.8 (163.3, 341.9) | <0.01 |
| Sodium, mg | 687.1 (458.2, 960.8) | 707.5 (486.4, 970.0) | 256.7 (221.7, 358.7) | <0.0001 |
| Sauces and Condiments (N) | 178 | 169 | 9 |  |
| Calcium, mg | 6.1 (2.2, 17.6) | 6.2 (2.7, 18.3) | 1.1 (0.8, 2.0) | 0.01 |
| Phosphorus, mg | 7.2 (1.6, 29.6) | 7.5 (2.0, 30.0) | 1.1 (0.7, 5.1) | 0.01 |
| Magnesium, mg | 2.5 (0.8, 7.2) | 2.9 (0.8, 7.2) | 0.6 (0.6, 1.3) | 0.03 |
| Iron, mg | 0.1 (0.0, 0.3) | 0.1 (0.0, 0.3) | 0.0 (0.0, 0.1) | <0.01 |
| Zinc, mg | 0.1 (0.0, 0.2) | 0.1 (0.0, 0.2) | 0.0 (0.0, 0.0) | 0.02 |
| Copper, mg | 0.0 (0.0, 0.0) | 0.0 (0.0, 0.0) | 0.0 (0.0, 0.0) | 0.01 |
| Selenium, mcg | 0.2 (0.1, 1.1) | 0.3 (0.1, 1.1) | 0.0 (0.0, 0.1) | <0.001 |
| Potassium, mg | 35.1 (10.2, 70.6) | 36.0 (11.7, 72.0) | 9.4 (8.2, 20.1) | 0.57 |
| Sodium, mg | 187.9 (12.4, 289.9) | 201.0 (24.4, 290.7) | 0.9 (0.2, 1.3) | <0.001 |
| Snacks and Desserts (N) | 680 | 671 | 9 |  |
| Calcium, mg | 21.6 (7.6, 51.6) | 21.8 (7.8, 52.9) | 5.6 (2.0, 28.6) | 0.02 |
| Phosphorus, mg | 54.9 (33.1, 86.4) | 54.6 (32.6, 86.9) | 64.8 (49.2, 67.3) | 0.53 |
| Magnesium, mg | 14.0 (7.8, 22.4) | 14.0 (7.8, 21.8) | 28.6 (21.0, 29.5) | 0.03 |
| Iron, mg | 0.7 (0.4, 1.3) | 0.7 (0.4, 1.3) | 0.4 (0.3, 0.5) | 0.28 |
| Zinc, mg | 0.3 (0.2, 0.5) | 0.3 (0.2, 0.5) | 0.5 (0.4, 0.6) | 0.17 |
| Copper, mg | 0.1 (0.0, 0.1) | 0.1 (0.0, 0.1) | 0.1 (0.1, 0.1) | 0.23 |
| Selenium, mcg | 2.8 (1.2, 6.1) | 2.8 (1.2, 6.1) | 3.1 (1.8, 4.4) | 0.74 |
| Potassium, mg | 78.8 (50.9, 129.4) | 79.1 (50.7, 130.9) | 68.6 (58.9, 77.0) | 0.85 |
| Sodium, mg | 144.1 (66.6, 231.2) | 144.9 (66.6, 234.4) | 73.8 (51.8, 78.8) | 0.18 |

1. Abbreviations: Reference amount customarily consumed (RACC), milligrams (mg), microgram (mcg), interquartile range (IQR)
2. P-values from t-tests comparing mean log-transformed nutrient values between FDA-aligned and FDA-unaligned groups
